# Genomewide meta‐analysis identifies loci associated with IGF‐I and IGFBP‐3 levels with impact on age‐related traits

**DOI:** 10.1111/acel.12490

**Published:** 2016-06-21

**Authors:** Alexander Teumer, Qibin Qi, Maria Nethander, Hugues Aschard, Stefania Bandinelli, Marian Beekman, Sonja I. Berndt, Martin Bidlingmaier, Linda Broer, CHARGE Longevity Working Group, Anne Cappola, Gian Paolo Ceda, Stephen Chanock, Ming‐Huei Chen, Tai C. Chen, Yii‐Der Ida Chen, Jonathan Chung, Fabiola Del Greco Miglianico, Joel Eriksson, Luigi Ferrucci, Nele Friedrich, Carsten Gnewuch, Mark O. Goodarzi, Niels Grarup, Tingwei Guo, Elke Hammer, Richard B. Hayes, Andrew A. Hicks, Albert Hofman, Jeanine J. Houwing‐Duistermaat, Frank Hu, David J. Hunter, Lise L. Husemoen, Aaron Isaacs, Kevin B. Jacobs, Joop A. M. J. L. Janssen, John‐Olov Jansson, Nico Jehmlich, Simon Johnson, Anders Juul, Magnus Karlsson, Tuomas O. Kilpelainen, Peter Kovacs, Peter Kraft, Chao Li, Allan Linneberg, Yongmei Liu, Ruth J. F. Loos, Body Composition Genetics Consortium, Mattias Lorentzon, Yingchang Lu, Marcello Maggio, Reedik Magi, James Meigs, Dan Mellström, Matthias Nauck, Anne B. Newman, Michael N. Pollak, Peter P. Pramstaller, Inga Prokopenko, Bruce M. Psaty, Martin Reincke, Eric B. Rimm, Jerome I. Rotter, Aude Saint Pierre, Claudia Schurmann, Sudha Seshadri, Klara Sjögren, P. Eline Slagboom, Howard D. Strickler, Michael Stumvoll, Yousin Suh, Qi Sun, Cuilin Zhang, Johan Svensson, Toshiko Tanaka, Archana Tare, Anke Tönjes, Hae‐Won Uh, Cornelia M. van Duijn, Diana van Heemst, Liesbeth Vandenput, Ramachandran S. Vasan, Uwe Völker, Sara M. Willems, Claes Ohlsson, Henri Wallaschofski, Robert C. Kaplan

**Affiliations:** ^1^ Institute for Community MedicineUniversity Medicine Greifswald17475GreifswaldGermany; ^2^ Interfaculty Institute for Genetics and Functional GenomicsUniversity Medicine Greifswald17475GreifswaldGermany; ^3^ Department of Epidemiology and Population HealthAlbert Einstein College of MedicineBronxNY10461USA; ^4^ Bioinformatics Core FacilityThe Sahlgrenska AcademyUniversity of GothenburgGothenburg40530Sweden; ^5^ Program in Genetic Epidemiology and Statistical GeneticsDepartment of EpidemiologyHarvard School of Public HealthBostonMA02115USA; ^6^ Geriatric UnitAzienda Sanitaria di Firenze (ASF)FlorenceItaly; ^7^ Molecular EpidemiologyLeiden University Medical CenterLeiden2300 RCThe Netherlands; ^8^ Division of Cancer Epidemiology and GeneticsNational Cancer InstituteBethesdaMD20892USA; ^9^ Medizinische Klinik und Poliklinik IVKlinikum der Universitaet Muenchen80336MunichGermany; ^10^ Department of EpidemiologySubdivision Genetic EpidemiologyErasmus Medical CenterPostbus 20403000 CARotterdamThe Netherlands; ^11^ Department of Internal MedicineErasmus Medical CenterPostbus 20403000 CARotterdamThe Netherlands; ^12^ Division of Endocrinology, Diabetes, and MetabolismDepartment of MedicineCenter for Clinical Epidemiology and BiostatisticsUniversity of Pennsylvania School of MedicinePhiladelphiaPA19104USA; ^13^ Section of GeriatricsDepartment of Clinical and Experimental MedicineUniversity of ParmaParmaItaly; ^14^ Geriatric Rehabilitation DepartmentUniversity‐Hospital of ParmaParmaItaly; ^15^ Framingham Heart StudyFraminghamMA01702USA; ^16^ Department of NeurologyBoston University School of MedicineBostonMA02118USA; ^17^ Section of Endocrinology, Diabetes & NutritionBoston University School of MedicineBostonMA02118USA; ^18^ Institute for Translational Genomics and Population SciencesLos Angeles BioMedical Research Institute at Harbor‐UCLA Medical CenterTorranceCA90502USA; ^19^ Department of GeneticsAlbert Einstein College of MedicineBronxNY10461USA; ^20^ Center for BiomedicineEuropean Academy Bozen/Bolzano (EURAC)Bolzano39100Italy; ^21^ Affiliated Institute of the University of Lübeck23562LübeckGermany; ^22^ Department of Internal Medicine and Clinical NutritionInstitute of MedicineSahlgrenska AcademyUniversity of Gothenburg41345GothenburgSweden; ^23^ Translational Gerontology BranchNational Institute on AgingBaltimoreMD21225USA; ^24^ Institute of Clinical Chemistry and Laboratory MedicineUniversity Medicine Greifswald17475GreifswaldGermany; ^25^ Institute for Clinical Chemistry and Laboratory MedicineRegensburg University Medical CenterD‐93053RegensburgGermany; ^26^ Division of Endocrinology, Diabetes and MetabolismDepartment of MedicineCedars‐Sinai Medical CenterLos AngelesCA90048USA; ^27^ The Novo Nordisk Foundation Center for Basic Metabolic ResearchSection of Metabolic GeneticsFaculty of Health and Medical SciencesUniversity of Copenhagen2100CopenhagenDenmark; ^28^ Division of EpidemiologyDepartment of Population HealthNew York University School of MedicineNew YorkNY10016USA; ^29^ Department of EpidemiologyErasmus University Medical CenterRotterdam3000 CAThe Netherlands; ^30^ Leiden University Medical CenterMedical Statistics and BioinformaticsLeiden2300 RCThe Netherlands; ^31^ Department of NutritionHarvard School of Public HealthBostonMA02115USA; ^32^ Channing Division of Network MedicineDepartment of MedicineBrigham and Women's Hospital and Harvard Medical SchoolBostonMA02115USA; ^33^ Department of Epidemiology and Statistical GeneticsHarvard School of Public HealthBostonMA02115USA; ^34^ Research Centre for Prevention and HealthCapital Region of DenmarkCopenhagenDK‐2600Denmark; ^35^ Genetic Epidemiology UnitDepartment of EpidemiologyErasmus University Medical CenterRotterdam3000 CAThe Netherland; ^36^ Centre for Medical Systems BiologyLeiden2300 RCThe Netherlands; ^37^ Department of Internal MedicineErasmus University Medical CenterRotterdam3000 CAThe Netherlands; ^38^ Department of PhysiologyInstitute of Neuroscience and PhysiologySahlgrenska AcademyUniversity of Gothenburg41345GothenburgSweden; ^39^ Department of ProteomicsHelmholtz – Centre for Environmental Research – UFZ04318LeipzigGermany; ^40^ Department of Growth and ReproductionRigshospitaletUniversity of CopenhagenCopenhagenDK‐2100Denmark; ^41^ Clinical and Molecular Osteoporosis Research UnitDepartment of Clinical SciencesLund University20502MalmöSweden; ^42^ IFB Adiposity DiseasesUniversity of Leipzig04103LeipzigGermany; ^43^ Department of BiostatisticsHarvard School of Public HealthBostonMA02115USA; ^44^ Department of Clinical Experimental ResearchGlostrup University HospitalCopenhagenDK‐2600Denmark; ^45^ Department of Clinical MedicineFaculty of Health and Medical SciencesUniversity of CopenhagenCopenhagenDK‐2200Denmark; ^46^ Division of Public Health SciencesDepartment of Epidemiology and PreventionWake Forest School of MedicineWinston‐SalemNC27157USA; ^47^ The Genetics of Obesity and Related Metabolic Traits ProgramThe Charles Bronfman Institute for Personalized MedicineThe Icahn School of Medicine at Mount SinaiNew YorkNY10029USA; ^48^ Estonian Genome CenterUniversity of TartuTartuEstonia; ^49^ Wellcome Trust Centre for Human GeneticsUniversity of OxfordOxfordUK; ^50^ General Medicine DivisionDepartment of MedicineMassachusetts General HospitalBostonMA02114USA; ^51^ Division of General MedicineHarvard Medical SchoolBostonMA02115USA; ^52^ Department of EpidemiologySchool of Public HealthUniversity of PittsburghPittsburghPA15261USA; ^53^ Departments of Experimental Medicine and OncologyMcGill UniversityMontréalQuébecCanadaH3A 0G4; ^54^ Department of Genomics of Common DiseaseSchool of Public HealthImperial College LondonLondonW12ONNUK; ^55^ Departments of Epidemiology, Medicine and Health ServicesUniversity of WashingtonSeattleWA98101USA; ^56^ INSERM U1078BrestFrance; ^57^ The Charles Bronfman Institute for Personalized MedicineIcahn School of Medicine at Mount SinaiNew YorkNY10029USA; ^58^ The Genetics of Obesity and Related Metabolic Traits ProgramIcahn School of Medicine at Mount SinaiNew YorkNY10029USA; ^59^ Department of MedicineAlbert Einstein College of MedicineBronxNY10461USA; ^60^ Institute of Aging ResearchGuangdong Medical CollegeDongguanChina; ^61^ Epidemiology BranchDivision of Epidemiology, Statistics and Prevention ResearchEunice Kennedy Shriver National Institute of Child Health and Human DevelopmentNational Institutes of Health6100 Executive BlvdRockvilleMD20852USA; ^62^ Medical DepartmentUniversity of Leipzig04103LeipzigGermany; ^63^ Gerontology and GeriatricsLeiden University Medical CenterLeiden2300 RCThe Netherlands; ^64^ Boston University and National HeartLung & Blood Institute's Framingham Heart StudyFraminghamMA01702USA; ^65^ Preventive Medicine & Epidemiology SectionBoston University School of MedicineBostonMA02118USA

**Keywords:** aging, genomewide association study, growth hormone axis, IGF‐I, IGFBP‐3, longevity

## Abstract

The growth hormone/insulin‐like growth factor (IGF) axis can be manipulated in animal models to promote longevity, and IGF‐related proteins including IGF‐I and IGF‐binding protein‐3 (IGFBP‐3) have also been implicated in risk of human diseases including cardiovascular diseases, diabetes, and cancer. Through genomewide association study of up to 30 884 adults of European ancestry from 21 studies, we confirmed and extended the list of previously identified loci associated with circulating IGF‐I and IGFBP‐3 concentrations (*IGF1, IGFBP3*,*GCKR*,*TNS3, GHSR, FOXO3, ASXL2, NUBP2/IGFALS, SORCS2*,* and CELSR2*). Significant sex interactions, which were characterized by different genotype–phenotype associations between men and women, were found only for associations of IGFBP‐3 concentrations with SNPs at the loci *IGFBP3* and *SORCS2*. Analyses of SNPs, gene expression, and protein levels suggested that interplay between *IGFBP3* and genes within the *NUBP2* locus (*IGFALS* and *HAGH)* may affect circulating IGF‐I and IGFBP‐3 concentrations. The IGF‐I‐decreasing allele of SNP rs934073, which is an eQTL of *ASXL2*, was associated with lower adiposity and higher likelihood of survival beyond 90 years. The known longevity‐associated variant rs2153960 (*FOXO3)* was observed to be a genomewide significant *SNP* for IGF‐I concentrations. Bioinformatics analysis suggested enrichment of putative regulatory elements among these IGF‐I‐ and IGFBP‐3‐associated loci, particularly of rs646776 at *CELSR2*. In conclusion, this study identified several loci associated with circulating IGF‐I and IGFBP‐3 concentrations and provides clues to the potential role of the IGF axis in mediating effects of known (*FOXO3*) and novel (*ASXL2*) longevity‐associated loci.

## Introduction

The insulin‐like growth factor (IGF) axis is an evolutionarily conserved system that plays important biologic roles in embryonic development, growth, and adulthood (Le Roith, 1997). IGF‐I mediates most of the activity of growth hormone (GH). The GH/IGF system consists of two ligands (IGF‐I and IGF‐II), six IGF‐binding proteins (IGFBP‐1‐6), and three IGF receptor subtypes (IGF‐I receptor, IGF‐II receptor, and insulin receptor) (Jones & Clemmons, 1995). IGF‐I promotes mitosis and cell cycle progression and is involved in human postnatal growth and development. Circulating IGF‐I is mainly bound to IGF‐binding proteins (IGFBPs) (Fowlkes, 1997), which affect activity (Lee *et al*., 1997) and half‐life of IGF‐I (Guler *et al*., 1989). From the clinical point of view, the measurement of IGF‐I and IGFBP‐3 blood concentrations is an important tool in establishing the diagnosis as well as monitoring treatment of GH‐related diseases (Ho & Participants, 2007; Cohen *et al*., 2008; Melmed *et al*., 2009).

Circulating concentrations of IGF‐I and IGFBP‐3 have been associated with risk of type 2 diabetes, cardiovascular diseases, cancer, and mortality in epidemiological studies (Juul *et al*., 2002; Vasan *et al*., 2003; Renehan *et al*., 2004; Kaplan *et al*., 2007; Friedrich *et al*., 2009; Burgers *et al*., 2011; Rajpathak *et al*., 2012). In animal models, diminished IGF‐I/insulin signaling has been associated with extended lifespans (Ziv & Hu, 2011), although the role of the IGF axis in human longevity remains inconclusive. Human genetic studies have suggested an association between polymorphisms in IGF‐I signaling pathway genes and longevity (Willcox *et al*., 2008; Ziv & Hu, 2011; Di Bona *et al*., 2014). Heritability studies have provided evidence for a substantial genetic contribution to circulating concentrations of IGF‐I (with heritability estimates ~40–60%) and IGFBP‐3 (80%) (Harrela *et al*., 1996; Hong *et al*., 1996; Souren *et al*., 2007). Identifying genetic determinants of circulating IGF‐I and IGFBP‐3 could lead to a better understanding of the biological basis of these factors in relation to human health and might identify pathways or susceptibility biomarkers that may assist with the development of interventions that target IGF‐I, its receptors, and binding proteins.

Our prior genomewide association study (GWAS) (*N* = 10 280) identified four loci with genomewide significant (*P* < 5 × 10^−8^) associations with circulating IGF‐I and IGFBP‐3 concentrations, including SNPs in or near *IGFBP3*,* TNS3*,* SORCS2*, and *NUBP2/IGFALS*, as well as three additional loci with suggestive associations (*P* < 1 × 10^−6^) in or near *RPA3*,* SPOCK2*, and *FOXO3* (Kaplan *et al*., 2011). Some of these genes are involved in well‐described IGF regulatory or signaling pathways (such as *IGFBP3* and *IGFALS*) (Deal *et al*., 2001; Gu *et al*., 2010; Schumacher *et al*., 2010) and are believed to influence traits that are also associated with concentrations or bioactivity of IGFs (e.g., *FOXO3* locus associated with longevity (Willcox *et al*., 2008) and *IGFBP3* locus associated with hip osteoarthritis (Evans *et al*., 2015)). To identify additional genetic variants with smaller effect sizes and enable sex specific analyses, we expanded our GWAS meta‐analysis to include up to a total of 30 884 individuals of European ancestry from 21 studies with measured circulating concentrations of IGF‐I and IGFBP‐3. In addition, using published GWAS data, we also performed lookups of associations of identified IGF‐I and IGFBP‐3 loci with survival beyond 90 years and other age‐related clinical traits.

## Results

### Characteristics of study samples

An overview of the study samples and data collection methods can be found in Tables S1 and S2 (Supporting information). Analyses of IGF‐I included up to 30 884 individuals (14 424 men and 16 460 women) from 21 studies and analyses of IGFBP‐3 included up to 18 995 individuals (8053 men and 10 942 women) from 13 studies.

### Loci associated with circulating IGF‐I and IGFBP‐3 concentrations

**Figure 1:**
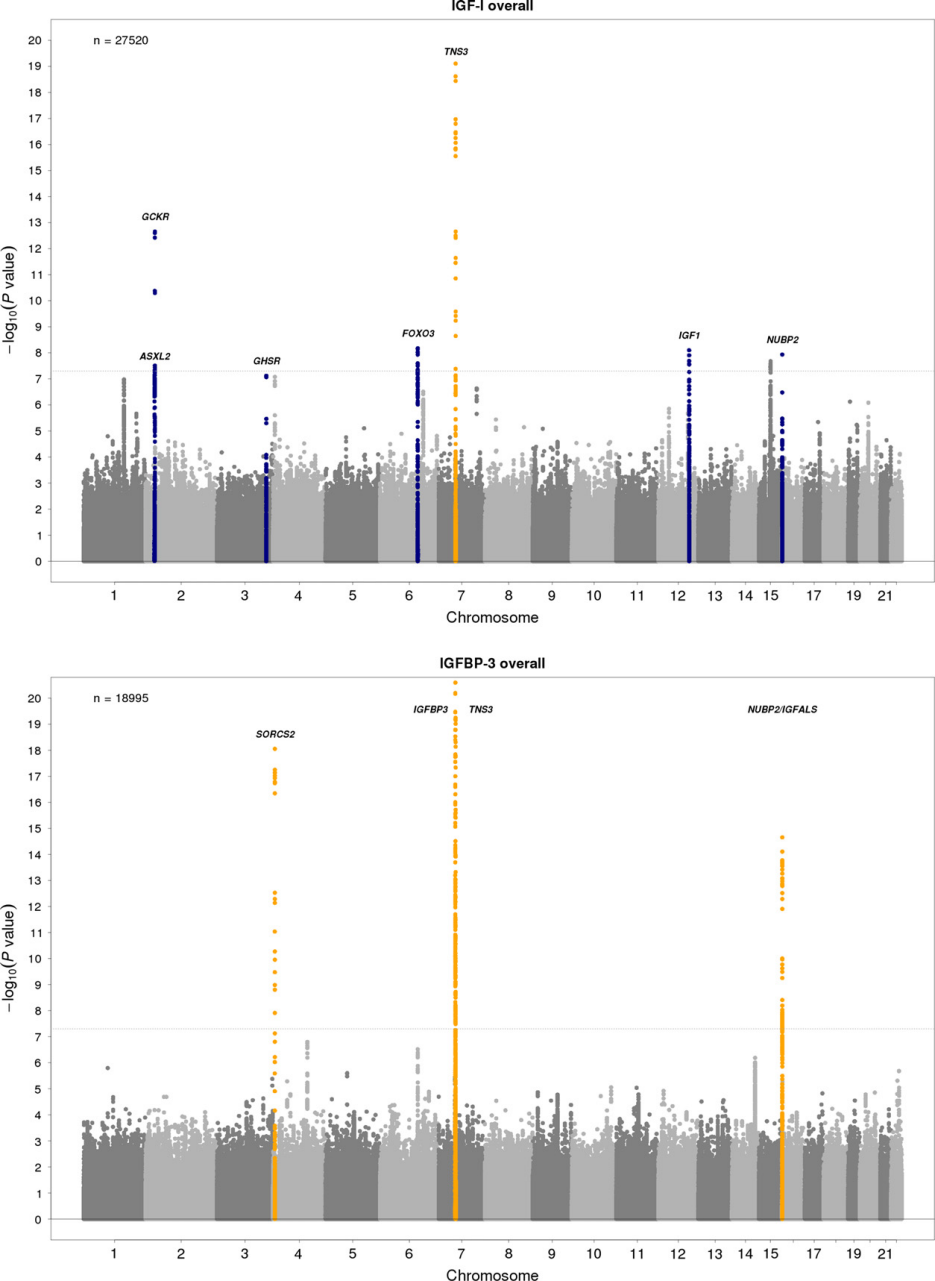
Manhattan plots of the combined stage 1 and 2 meta‐analysis results of IGF‐I and IGFBP‐3 traits in the men and women combined sample. SNPs are plotted on the *x*‐axis according to their position on each chromosome with the ‐log10 association *P*‐value on the *y*‐axis. The upper solid horizontal line indicates the threshold for genomewide significance. Known hits are colored in orange and new findings in blue. Plots are truncated on the *y*‐axis to 20.

An overview of the GWAS meta‐analysis results is given by the Manhattan plots in Fig. 1 and in Fig. S1 (Supporting information). There was no indication of inflated test statistics (i.e., due to unaccounted population stratification) as seen by the quantile–quantile (QQ) plots, and genomic control lambda ranged from 1.02 to 1.08 for the meta‐analysis results (Fig. S2, Supporting information) and from 0.98 to 1.08 (median 1.01) for the individual GWAS results. All lead SNPs (independent SNPs with the smallest *P*‐value within a locus, see Methods) had a good imputation quality across the studies (median imputation quality >0.9).

After the final stage, which combines results of stages 1 and 2 plus *de novo* genotyping in stage 3, we found seven genomewide significant loci (*P* < 5.0 × 10^−8^) associated with circulating IGF‐I concentration (Table 1). In addition to the known locus near *TNS3*, we identified new loci in or near *GCKR*,* IGF1*,* FOXO3*,* ASXL2*,* NUBP2,* and *GHSR* associated with IGF‐I concentrations.

**Table 1:** Loci associated with IGF‐I and IGFBP‐3 concentrations in men and women combined samples at genomewide significance (*P* < 5 × 10^−8^) after final stage

Trait	SNP	A1	A2	F1	*P*	I²	Chr	Position	Nearest gene	Gene distance	Direction effect	
											IGF‐I	IGFBP‐3
IGF‐I^a^	rs700753	C	G	0.35	1.60E‐23	4.2	7	46,720,209	*TNS3*	561067	*–*	*–*
IGF‐I	rs780093	T	C	0.41	2.19E‐13	24.5	2	27,596,107	*GCKR*	0	*–*	+
IGF‐I	rs978458	T	C	0.26	1.56E‐10	0.0	12	101,326,369	*IGF1*	0	*+*	–
IGF‐I	rs2153960	A	G	0.69	5.16E‐09	22.5	6	109,082,339	*FOXO3*	0	*+*	+
IGF‐I	rs934073	C	G	0.71	6.48E‐09	21.8	2	25,790,669	*ASXL2*	25087	*–*	–
IGF‐I	rs1065656	C	G	0.31	1.17E‐08	47.9	16	1,778,837	*NUBP2*	0	*–*	*–*
IGF‐I	rs509035	A	G	0.31	2.09E‐08	0.0	3	173,646,143	*GHSR*	0	*+*	+
IGFBP‐3^a^	rs11977526	A	G	0.41	4.16E‐161	51.5	7	45,974,635	*IGFBP3*	47239		
IGFBP‐3^a^	rs700753	C	G	0.35	1.11E‐46	26.7	7	46,720,209	*TNS3*	561067	–	*+*
IGFBP‐3^a^	rs1065656	C	G	0.31	8.55E‐23	24.1	16	1,778,837	*NUBP2*	0	–	*–*
IGFBP‐3^a^	rs4234798	T	G	0.39	8.86E‐19	0.0	4	7,270,834	*SORCS2*	0	–	*–*
Bivariate analysis	rs646776	T	C	0.78	6.87E‐9	26.1/43.1	1	109,620,053	*CELSR2*	152	–	*–*

‘−’ = coding allele associated with lower IGF‐1 and IGFBP‐3 levels (indicated by bold italicized text were genomewide significant); ‘+’ = coding allele associated with higher IGF‐1 and IGFBP‐3 levels (indicated by bold italicized text were genomewide significant); Chr = chromosome; A1 = coding allele; A2 = other allele; F1 = frequency of coding allele.

a Known association.

We found genomewide significant associations with IGFBP‐3 concentration for SNPs in or near *IGFBP3*,* TNS3*,* NUBP2,* and *SORCS2,* thus confirming all four previously known loci (Table 1). The SNPs at *TNS3* and *NUBP2* were genomewide significantly associated with both IGF‐I and IGFBP‐3 concentrations and had the same direction of effect for each circulating protein.

For six of ten genomewide significant SNPs, effects were in the same direction of association for IGF‐I concentrations and IGFBP‐3 concentrations (Table 1).

Detailed results of the significant associations after the final stage can be found in Table 1. Results of the individual analysis stages appear in Table S3 (Supporting information). Regional association plots are shown in Figs S3 and S4 (Supporting information).

### Bivariate analysis of IGF‐I and IGFBP‐3

We performed a bivariate analysis of IGF‐I and IGFBP‐3. By leveraging shared variance between the two outcomes, this analysis can have improved power to identify SNPs associated with both IGF‐I and IGFBP‐3 concentrations, especially in the case of SNPs that have opposite effects on positively correlated traits (Aschard *et al*., 2014). The bivariate analysis identified a new locus at *CELSR2* (Table 1), which had nominal association with IGFBP‐3 (*P *=* *2.08 × 10^−05^) and IGF‐1 (*P *=* *0.0096) in the univariate analysis. SNP rs646776 at *CELSR2* had opposite effects on the two traits, being negatively associated with IGF‐1 and positively associated with IGFBP‐3 (Table 1, Table S4 and Fig. S5, Supporting information). In addition, SNPs at *IGFBP3*,* TNS3*,* NUBP2*,* SORCS2*,* GCKR*,* IGF1*, and *FOXO3,* identified in the univariate analysis, also showed genomewide significant associations in the bivariate analysis.

### Interaction by sex

The sex‐stratified analyses revealed no additional discoveries that were not detected in the overall population. Although the direction of effect was similar for *IGFBP3* and *SORCS2* SNPs within sex subgroups, these two SNPs were found to have significantly different association effect sizes between men and women for IGFBP‐3, consistent with stronger associations in women. These findings of sex interaction maintained statistical significance after Bonferroni correction for the 12 tested genomewide significant lead SNPs (*P* < 0.004) (Table S5, Supporting information).

### Gene‐based analysis (vegas)

Gene‐based analyses showed several significant IGF‐I‐associated genes within or close to the *GCKR* GWAS locus: *EIF2B4*,* FNDC4*,* GCKR*,* IFT172*,* PPM1G*,* SNX17*,* ZNF513*,* GTF3C2*, K*RTCAP3*,* MPV17*, and *NRBP1* (associated with IGF‐I). New gene‐based associations that were not covered by a single SNP GWAS association were found for *C6orf173* (chromosome 6) on IGF‐I concentration. The following genes of the *NUBP2* GWAS locus were associated with circulating IGFBP‐3 concentration: *EME2*,* IGFALS*,* MAPK8IP3*,* MRPS34*,* NME3*,* NUBP2*,* HS3ST6*,* RPL3L*,* SEPX1,* and *SPSB*. Two genes, *IGFBP1* and *IGFBP3*, at the *IGFBP3* locus were associated IGFBP‐3 concentration.

### Lookup for expression quantitative trait loci associations

A lookup of the lead SNPs for *cis* expression quantitative trait loci (eQTL) was performed in the publicly available database of whole blood eQTL associations (Westra *et al*., 2013). For the following SNPs, one or more *cis* eQTL associations were found: rs1065656 (*NUBP2*), rs11977526 (*IGFBP3*), rs2153960 (*FOXO3*), rs509035 (*GHSR*), rs780093 (*GCKR*), rs934073 (*ASXL2*), and rs978458 (*IGF1*) (Table 2).

**Table 2:** Results of significant whole blood eQTL associations of the genomewide significant lead SNPs

SNP	GWAS locus	eQTL p‐value	Chr	Probe center position	Probe name	SNP alleles	Effect allele	Effect direction	EQTL gene
rs1065656	*NUBP2*	3.66E‐04	16	1,829,861	1710332	C/G	C	+	*FAHD1*
rs1065656	*NUBP2*	4.28E‐12	16	1,799,259	**4900333**	C/G	C	−	***HAGH***
rs1065656	*NUBP2*	3.78E‐04	16	1,809,162	**1780356**	C/G	C	−	***HAGH***
rs1065656	*NUBP2*	9.35E‐04	16	1,760,172	5270575	C/G	C	+	*MAPK8IP3*
rs1065656	*NUBP2*	3.34E‐05	16	1,762,997	6110307	C/G	C	+	*MRPS34*
rs1065656	*NUBP2*	7.75E‐34	16	1,760,510	6450424	C/G	C	+	*NME3*
rs1065656	*NUBP2*	1.09E‐14	16	1,778,738	6960730	C/G	C	−	*NUBP2*
rs1065656	*NUBP2*	9.87E‐05	16	1,766,816	2850433	C/G	C	−	*SPSB3*
rs2153960	*FOXO3*	2.95E‐06	6	109,129,272	7200189	G/A	G	−	*HS.133419*
rs509035	*GHSR*	5.97E‐05	3	173,706,575	870202	G/A	A	+	*TNFSF10*
rs780093	*GCKR*	2.46E‐04	2	27,440,911	5960546	T/C	T	−	*EIF2B4*
rs780093	*GCKR*	5.70E‐04	2	27,440,904	6370494	T/C	T	−	*EIF2B4*
rs780093	*GCKR*	2.69E‐05	2	27,518,384	430239	T/C	T	+	*NRBP1*
rs780093	*GCKR*	1.00E‐10	2	27,453,289	3360468	T/C	T	+	*SNX17*
rs934073	*ASXL2*	5.96E‐04	2	25,816,038	650075	G/C	G	+	*ASXL2*
rs978458	*IGF1*	1.71E‐03	12	101,115,257	990136	T/C	T	+	*C12ORF48*
rs11977526	*IGFBP3*	1.84E‐05	7	45,918,692	6840372	G/A	A	−	*IGFBP3*

Chr, chromosome; eQTL, expression quantitative trait loci; GWSD, genomewide association study.

mRNA of probe and gene names marked in bold showed also significant association with circulating IGFBP‐3 levels (*P *<* *3.5 × 10^−4^).

Additionally, using a similar strategy we performed lookup in the MuTHER consortium (Grundberg *et al*., 2012) for *cis* eQTL associations found in fat cell, skin cell, and lymphoblastic cell lines (LCL). After Bonferroni correction for 297 lookups of three different traits (*P* < 5.6 × 10^−5^), rs1065656 (*NUBP2*) showed significant associations, specifically with *FAHD1* (all tissues) and *HAGH* (fat cells and LCL) (Table S6, Supporting information). Furthermore, both genes were also significant in whole blood *cis* eQTL for the SNP rs1065656 (*NUBP2*) (Table 2).

### Associations of gene expression with circulating IGF‐I and IGFBP‐3 concentrations

We next sought to link associations between SNPs and circulating IGF‐I and IGFBP‐3 concentrations, with *cis* eQTL associations of the same SNPs. In the 986 samples of the SHIP‐TREND cohort, we examined associations between whole blood mRNA expression levels of the genes located in a 500‐kb vicinity of our significant lead SNPs and circulating IGF‐I and IGFBP‐3 concentrations. Significance of the 323 array probe trait associations was defined by a false discovery rate (FDR) <0.05. Only mRNA levels of genes in vicinity of the *NUBP2* GWAS locus were significantly associated with IGF‐I concentration (gene *SEPX1*) or IGFBP‐3 concentration (genes *HAGH* and *RPS2*). Of note, *HAGH* was the gene on which the corresponding lead SNP (rs1065656) had also a significant *cis* eQTL. The complete gene expression association results are listed in Table S7 (Supporting information).

### Association with plasma protein levels

In 197 samples of the SHIP‐TREND cohort, peptides of the following proteins that were encoded by genes in a 500‐kb vicinity of the lead SNPs were examined for protein quantitative trait analyses (pQTL): insulin‐like growth factor‐binding protein complex acid labile subunit (ALS encoded by *IGFALS* at the *NUBP2* locus), 28S ribosomal protein S34, mitochondrial (RT34 encoded by *MRPS34* at the *NUBP2* locus), insulin‐like growth factor‐binding protein 3 (IBP3 encoded by *IGFBP3* at the *IGFBP3* locus), and coiled‐coil domain‐containing protein 121 (CC121 encoded by *CCDC121* at the *GCKR* locus). Of the 32 tested SNP peptide pairs, peptides of the ALS protein had significant pQTL at FDR <0.05 (Table S8, Supporting information). Furthermore, strong associations were found in the same samples for the pQTL‐associated peptides of ALS and IBP3 with circulating levels of IGF‐I and IGFBP‐3 (*P *<* *1.0 × 10^−6^).

### Allelic heterogeneity of the *NUBP2* locus

To further examine the *NUBP2* locus, we performed a conditional analysis of this locus based on the meta‐analysis results adjusting for the lead SNP rs1065656. The analyses revealed an independent genomewide significant association of a second SNP rs11644716 with IGFBP‐3 (*P* = 6.3 × 10^−14^) and an opposite effect direction of the minor allele C (MAF = 0.05) compared with the lead SNP rs1065656 (MAF = 0.31) (*r*² = 0.03 between these two SNPs based on the HapMap R22 reference data). Although not genomewide significant, rs11644716 was also associated with circulating IGF‐I concentration (*P* = 1.8 × 10^−6^) and has an eQTL for *HAGH* (probe 4900333: *P* = 3.2 × 10^−5^; probe 1780356: *P* = 0.003) and a pQTL with ALS (eight peptides with *P*‐value <0.01). In all cases, the effect directions based on the minor allele of rs11644716 were opposite of that for the minor allele of rs1065656.

**Figure 2:**
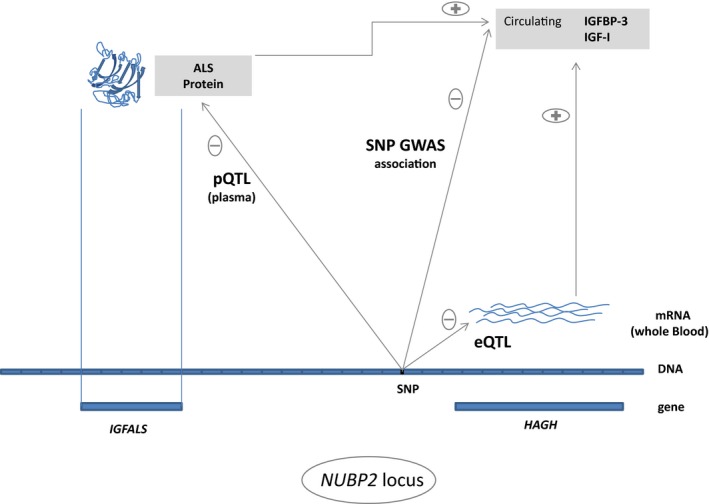
Overview of the *NUBP2* locus that shows the relations of gene expression levels, protein levels and circulating IGF‐I and IGFBP‐3 with respect to the SNP. Effect directions (±) are given for the minor C‐allele of SNP rs1065656 (MAF 31%). Note: the second signal rs11644716 (MAF 5%) has opposite expression quantitative trait loci (eQTL) and protein quantitative trait analyses (pQTL) effect directions to rs1065656, but they are consistent.

Figure 2 summarizes the relationship between the lead SNPs at the *NUBP2* locus and gene expression levels, protein levels, and circulating IGF‐I and IGFBP‐3 concentrations.

### Associations with serum metabolites

Lead SNPs associated with IGF‐I and IGFBP‐3 concentrations were examined in a published metabolite‐SNP association database (Suhre *et al*., 2011; Shin *et al*., 2014). The IGF‐I‐associated SNP rs780093 at *GCKR* locus was associated with glucose/mannose ratio (*P *=* *9.4 × 10^−143^), and the IGFBP‐3‐associated SNP rs4234798 at *SORCS2* locus was associated with caprylate (8:0)/phenylalanine ratio (*P *=* *7.3 × 10^−7^).

### Associations of top loci with age‐related traits

We also examined the associations of the IGF‐I‐ and IGFBP‐3‐associated SNPs with anthropometric traits (height, BMI, waist‐to‐hip ratio, and fat percentage) (Heid *et al*., 2010; Lango Allen *et al*., 2010; Speliotes *et al*., 2010), bone mineral density (Estrada *et al*., 2012), risk of type 2 diabetes (Voight *et al*., 2010; Morris *et al*., 2012) and related traits (fasting glucose, 2‐h glucose, HbA1c, fasting insulin, proinsulin, HOMA‐IR, and HOMA‐B) (Dupuis *et al*., 2010; Saxena *et al*., 2010; Soranzo *et al*., 2010), and coronary artery disease (Coronary Artery Disease Genetics C, 2011; Schunkert *et al*., 2011; Consortium CAD and Deloukas, 2013) (Table S9, Supporting information). Many nominal associations were expected because of the known influence of the IGF system on these traits. Of note is the finding that for rs780093 in the *GCKR* locus, the allele associated with higher IGF‐I concentration was already known to be associated with elevated risk of type 2 diabetes (*P* = 3.7 × 10^−6^), as well as higher levels of fasting glucose, fasting insulin, and HOMA‐IR (all *P* < 2.0 × 10^−4^), lower 2‐h glucose levels (*P* = 1.7 × 10^−6^), increased height (*P* = 2.0 × 10^−4^), lower waist‐to‐hip ratio (*P* = 0.0003), and higher lumbar spine bone mineral density (*P* = 0.002).

Three additional loci (*GHSR*,* CELSR2*, and *FOXO3*) showed strong associations with height (all *P* < 1.0 × 10^−4^). The IGFBP‐3‐increasing allele of SNP rs646776 (*CELSR2* locus) was associated with increased risk of coronary artery disease (*P* = 9.4 × 10^−15^). The IGF‐I‐decreasing allele of SNP rs934073 at *ASXL2* showed a nominal association with survival beyond 90 years (*P *=* *0.018) as well as higher levels of BMI (*P* = 0.008) and fat percentage (*P* = 9.4 × 10^−5^) and lower lumbar spine bone mineral density (*P* = 0.004).

We further performed lookups of top IGF‐I‐ and IGFBP‐3‐associated SNPs (*P* < 10^−6^ in the meta‐analysis of stage 1 and stage 2) for associations with survival beyond 90 years using published GWAS data (Broer *et al*., 2015) (Tables S10 and S11, Supporting information). Among 15 independent circulating IGF‐I‐associated SNPs defined based on linkage disequilibrium (LD) (settings *r* ^2^ > 0.01, 1 Mb distance), the SNP rs10457180 (*r* ^2^ = 0.96 with the lead SNP rs2153960) at *FOXO3 (P = 8.6 × 10* ^*−5*^ *)* and SNP rs11892454 (*r* ^2^ = 0.71 with the lead SNP rs934073) at *ASXL2 (P = 0.003)* reached statistical significance after Bonferroni correction for 15 independent tests. Among 13 independent circulating IGFBP‐3‐associated SNPs, the SNP rs9398172 (*r* ^2 ^= 1 with the lead SNP rs2153960) at *FOXO3* (*P = 2.5 *×* *10^−4^) remained significantly associated with survival beyond 90 years after Bonferroni correction.

### Enrichment of putative regulatory elements among loci associated with circulating IGF‐1 and IGFBP‐3 concentrations

**Figure 3:**
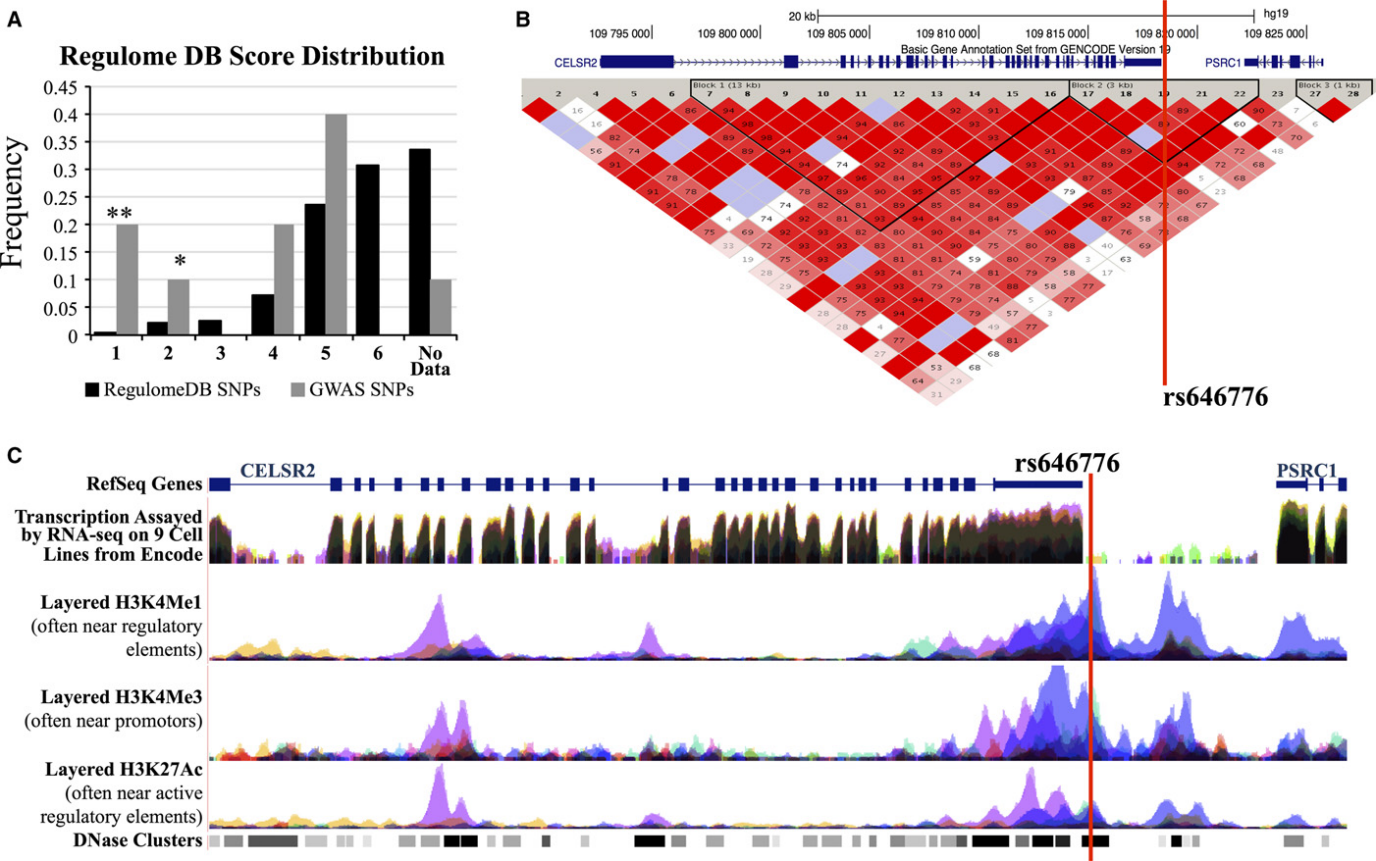
Analysis of regulatory element marks in loci associated with serum IGF‐1 and IGFBP‐3 concentrations. (A) IGF‐1‐ and IGFBP‐3‐associated SNPs are enriched for putative regulatory elements compared with all SNPs in RegulomeDB. **P < 0.005, *P < 0.05 (Monte Carlo). Overall distribution of genomewide association study (GWAS) SNPs vs. RegulomeDB SNPs *P* < 2.2E‐16, multinomial method. (B) Genomic context surrounding rs646776 showing *R* ^2^ values. (C) Representative regulatory motif tracks from USCS Genome Browser and encode showing histone mark peaks and DNase hypersensitivity at the location of rs646776.

We examined whether the identified SNPs fall within regulatory elements in the epigenetic encode and roadmap data for associated SNPs using Haploreg (http://www.broadinstitute.org/mammals/haploreg/haploreg.php) (Ward & Kellis, 2012) and RegulomeDB (http://regulomedb.org/) (Boyle *et al*., 2012) (Table S11, Supporting information). Lower scores indicate stronger evidence for the presence of a regulatory element. To determine whether these IGF‐I‐ and IGFBP‐3‐associated loci are enriched for regions likely to affect gene expression, we further examined the distribution of scores among these SNPs compared with all RegulomeDB SNPs. We found that these identified SNPs are highly enriched for low Regulome scores (*P* < 2.2 × 10^−16^ by multinomial method, Fig. 3A). The genomic and representative epigenetic context surrounding rs646776 (*CELSR2* locus), a SNP with a Regulome score of 1f, is shown as an example (Fig. 3). SNP rs646776 localizes to a genomic region of high LD (Fig. 3B) and lies within peaks of histone marks associated with regulatory elements, and a DNase hypersensitivity region (Fig. 3C)(Kent *et al*., 2002). In addition, SNP rs646776 falls in ChIP identified binding regions for CTCF, POLR2A, REST, and TAF7 (data not shown).

## Discussion

Our second GWAS report from the CHARGE IGF Working Group, here expanded to include more than >30 000 individuals, revealed several SNPs influencing circulating levels of IGF‐I and IGFBP‐3 which also have been associated with other metabolic and age‐related traits. These included several loci already implicated in the biology of GH/IGF‐I (*IGF1, IGFBP3, IGFALS, GHSR, FOXO3*) as well other novel findings including rs934073 SNP on chr 2, which is an eQTL for polycomb group gene *ASXL2* associated with reduced circulating IGF‐I. Cross‐reference of IGF‐I‐ and IGFBP‐3‐associated SNPs against published GWAS of age‐related traits identified the *ASXL2* SNP as a (to our knowledge) novel locus for longevity as defined as survival beyond 90 years. All genomewide significant associations with IGFBP‐3 and IGF‐I levels that were reported in our preceding study (Kaplan *et al*., 2011) could be confirmed here using a larger sample. Additionally, our preliminary finding of an association of circulating IGF‐I level with rs2153960 in the *FOXO3* gene reached genomewide significance using this larger sample size. Moreover, bioinformatics analysis also suggests enrichment of putative regulatory elements among these IGF‐I‐ and IGFBP‐3‐associated loci, particularly of rs646776 at *CELSR2*.

Our study reveals clues about mechanisms of IGF system regulation through the interplay of IGFBP‐3 and genes within the *NUBP2* locus, including *IGFALS* and *HAGH*. The *IGFALS* encodes the insulin‐like growth factor‐binding protein complex, acid labile subunit protein (ALS) which forms a ternary complex with IGF‐I and IGFBP‐3 (Firth *et al*., 1998; Twigg & Baxter, 1998). Like in most GWAS, our analyses cannot establish which is the causative SNP or gene of a locus. However, *IGFALS* seems to be a strong candidate supported by the associations of SNPs in the vicinity of *IGFALS* with both circulating IGFBP‐3 and plasma levels of the protein coded by *IGFALS*. Although the sample size for the plasma proteome analyses was restricted to 197 probands of SHIP‐TREND, the observed associations of the second signal rs11644716 achieved a moderately high level of statistical significance (*P* < 0.0015). *IGFALS* was not abundantly expressed in whole blood cells, and its transcript levels were neither associated with IGF‐I nor IGFBP‐3 concentration. Certainly, the protein ALS encoded by *IGFALS* is abundant in serum, but the gene is translated in liver. In contrast to *IGFALS*,* HAGH* (hydroxyacylglutathione hydrolase) was sufficiently expressed in whole blood cells and the amount of its mRNA was strongly correlated with circulating IGFBP‐3 serum concentration. Taking into account the eQTL association of the lead SNP rs1065656 with *HAGH* and the correlation with the mRNA and IGFBP‐3, this SNP might influence the IGFBP‐3 levels by modulating the amount of *HAGH* mRNA, whereas both the levels of mRNA and IGFBP‐3 are reduced per copy of the minor allele. Although this chain of associations was revealed in whole blood, it might be present in other tissues as well because significant eQTL for rs1065656 with *HAGH* were observed in other tissues studied in the MuTHER dataset (Grundberg *et al*., 2012). Given the more pronounced association with ALS and the less significant eQTL with *HAGH* of the second signal compared with the lead SNP, rs11644716 might reduce the level of circulating IGFBP‐3 indirectly by reducing the amount of ALS per minor allele. Of note, there was no nonsynonymous SNP in LD in the 1000 Genomes v3 dataset (*R*² > 0.8, SHIP cohort) for both SNPs which could have helped narrow down the functional mechanism.

Taken together with genotype–phenotype association data assembled by others, our study revealed that IGF‐I‐ and IGFBP‐3‐associated SNPs had expected associations with anthropometric and age‐related chronic disease traits (e.g., bone mineral density, disordered carbohydrate metabolism). We also found that SNPs associated with reduced IGF‐I levels tended to be associated with longer survival defined as death after 90 years (Broer *et al*., 2015). This is consistent with an observation from prior analysis of candidate genes associated with the insulin and IGF signaling axis (van Heemst *et al*., 2005). In addition, rs934073, an eQTL for additional sex comb‐like 2 (*ASXL2*), was a genomewide significant SNP associated with lower IGF‐I level which also has enriched frequency in adults older than 90 years of age (Broer *et al*., 2015). Other traits associated with the IGF‐I‐decreasing allele of rs934073 included greater adiposity and reduced lumbar spine (but not femoral neck) bone mineral density. *ASXL2* is a polycomb group protein with known functions in development that has also been associated with pediatric cancer, but to our knowledge has not before been suggested as a longevity gene (Huether *et al*., 2014). The SNP in the known longevity gene *FOXO3* has not only been previously associated with reduced fasting insulin and HOMA‐IR (Willcox *et al*., 2008), but here it was found to produce lower circulating IGF‐I levels. Multiple genetic determinants for circulating IGF‐I in normal and IGF1R resistance states might partially explain the U‐shaped association of circulating IGF‐I concentration with mortality (Suh *et al*., 2008; Burgers *et al*., 2011).

While our meta‐analysis encompasses a large number of samples from multiple cohorts, this may lead to limitations. Given the different origin of the cohorts (Table S1, Supporting information), heterogeneity in the association results might occur due to the different genetic background and the patterns of intake of nutrients across the individual studies.

In summary, this project extends our prior work (Kaplan *et al*., 2011) through the identification of several new loci related to circulating IGF‐I and IGFBP‐3 levels that also may affect aging. While the effects of insulin/IGF‐I signaling on survival often displays sex dimorphism in humans and other organisms, we found similar genetic determinants of IGF protein levels in men and women, even with relatively large sample size finding interaction by sex only for two loci (*IGFBP3* and *SORCS2*) in association with circulating IGFBP‐3. Finally, a novel identified gene candidate for long‐term survival, *ASXL2*, requires further study. Taking into account the design of our study, most of the findings should be considered as important and well‐grounded hypotheses to work on.

## Experimental procedures

### Participating studies

In total, the CHARGE IGF Working Group included 21 and 13 studies that participated in the association analysis for IGF‐I (*N* = 27 520, 53% women) and IGFBP‐3 (*N* = 18 995, 58% women), respectively. Four of the cohorts (*N* = 10 280) were previously included in a GWAS meta‐analysis of IGF‐I and IGFBP‐3 levels (Kaplan *et al*., 2011). Imputed SNPs for chromosome X were available for 16 670 and 11 959 individuals with IGF‐I and IGFBP‐3 measurements, respectively. Additionally, up to 3364 individuals (55% women) with IGF‐I from one study were available for *de novo* genotyping of selected SNPs. Detailed information on participant characteristics, IGF‐I and IGFBP‐3 measurements, and genotyping of all studies participated in the different analyses and stages is given in Table S2 (Supporting information). All participants provided informed consent, and human subjects’ research review was obtained from each participating cohort.

### Statistical analyses

#### GWAS in individual studies

Each study of the GWAS stages performed genotyping on genomewide arrays and imputed SNPs using the HapMap2 reference panel. Detailed information on genotyping and imputation is provided in Table S2 (Supporting information). Association analyses in individual studies were performed on IGF‐I and IGFBP‐3 levels measured in ng mL^−1^ using a multiple linear regression with an additive genetic model based on allele dosages adjusted for age and stratified by sex. All cohorts accounted for relatedness, population substructure using genetic principal components, study center, and laboratory batch of IGF measurement where applicable. Individuals of non‐European ancestry, with missing phenotypic data, diagnosed growth hormone deficiency, or known use of human growth hormones were excluded prior to the analyses.

#### Meta‐analysis

From each cohort's result file, monomorphic SNPs as well as SNPs with an imputation quality below 0.3 were excluded prior to the meta‐analysis. All study‐specific GWAS results were corrected by the genomic inflation factor λ_GC_ if >1. Due to the IGF‐I and IGFBP‐3 assay‐based differences in both effect sizes and variances of measurements across cohorts, a sample size‐weighted z‐score‐based meta‐analysis implemented in METAL (Willer *et al*., 2010) was conducted, and the meta‐analysis *P*‐values were corrected for genomic inflation. After meta‐analysis, SNPs with a MAF ≤1% were removed from subsequent analyses.

Our multistage design had two GWAS stages (stages 1 and 2) and an additional stage (stage 3) with *de novo* genotyping data (*N* = 3364 individuals) to confirm novel loci. After stage 1 GWAS, all 19 lead SNPs from all traits with a *P* < 10^−6^ were taken forward to stage 2. All IGF‐I lead SNPs of novel loci that had a combined stage 1 and stage 2 *P* < 10^−8^ (except *GCKR*) were selected for *de novo* replication in an additional cohort. An overview of the design and the significantly associated loci at each stage is provided in Fig. S6 (Supporting information). Details on SNP selection and quality control are given in the Appendix S1 (Supporting information). Regional association plots were generated using LocusZoom (Pruim *et al*., 2010).

#### Assessment of independent signals

To define a lead SNP of each locus, the association results of a GWAS stage with *P*‐values <1 × 10^−5^ were grouped based on the LD structure of the HapMap release 28 CEU dataset using PLINK (settings *r* ^2^ >0.01, 1 Mb distance) (Purcell *et al*., 2007). Due to the strong association of the *IGFBP3* locus with IGFBP‐3, only one lead SNP was selected regardless of several grouped results.

The analysis of secondary signals in the *NUBP2* locus was performed using the software gcta (Yang *et al*., 2011) and the genotypes of the SHIP cohort as a reference, and was verified by an analysis using the genotypes of the NHS/HPFS cohorts as a reference.

#### Sex interaction analysis

Sex interactions on IGF‐I and IGFBP‐3 levels were obtained by comparing, for each SNP, the stage 2 meta‐analysis *z*‐scores from men (z_men) (IGF‐I: *N* = 12 917, IGFBP‐3: *N* = 8052) and women (z_women) (IGF‐I: *N* = 14 602, IGFBP‐3: *N* = 10 942) using the formula z_interaction=(z_men‐z_women)/√2, assuming independent effect sizes between men and women, and matched to a common effect allele.

#### Bivariate meta‐analysis of IGF‐I and IGFBP‐3

The stage 2 meta‐analysis *z*‐scores of the combined samples IGF‐I and IGFBP‐3 were used to calculate a bivariate meta‐analysis implemented in the function multipheno.T2 of the r‐package gtx (version 0.0.8. http://CRAN.R-project.org/package=gtx). The function corresponds closely with Hotelling's *T* ^2^ test and calculates a multiphenotype association test for each marker based on the meta‐analysis result *z*‐statistics that is equivalent to using the subject‐specific data to perform a multivariate analysis of variance.

#### Gene‐based analysis

Genomewide gene‐based tests which account for both gene length and LD between SNPs were performed by vegas 0.8.27 (Versatile Gene‐Based Association Study) (Liu *et al*., 2010) using SNP *P*‐value results from the overall meta‐analyses. SNPs were allocated to one or more autosomal genes using gene boundaries ±50 kb. We performed 1 × 10^7^ permutations and defined a gene‐based *P*‐value <1 × 10^−6^ as gene‐based genomewide significant.

#### Gene expression and eQTL analysis

For each of the lead SNPs of the significant loci after final stage, significant cis eQTL associations in whole blood, lymphocytes, subcutaneous fat, muscle, and skin were looked up in the publically available association result databases (Grundberg *et al*., 2012; Westra *et al*., 2013). Association analysis of whole blood gene expression data with serum IGF‐I and IGFBP‐3 levels was conducted in 986 samples of the SHIP‐TREND cohort (Schurmann *et al*., 2012).

#### Association with plasma protein levels

Plasma proteome data were obtained as described in Appendix S1 (Supporting information) using liquid chromatography–mass spectrometry (LC‐MS). mascot (in‐house mascot server v2.3.2; Matrix Science, London, GB) search algorithm was used to match the generated peak lists with a human fasta‐formatted database containing 20 268 unique sequence entries (reviewed human database, release of October 2011). Prior to data analyses, all peptide intensity values were log10‐transformed and median–median‐normalized. Association analyses between peptides and serum IGF‐I and IGFBP‐3 levels were performed by linear regression, adjusted for age, sex, and the MS processing batch. Associations of a SNP with the peptides were conducted by linear regression, adjusted for age, sex, and the first four principal components of a peptide‐level‐based principal component analysis. Protein intensities used for analyses were obtained by averaging the corresponding peptide intensities that passed the QC filter, and were put instead of the peptide intensities into the association model. All measured peptides that passed QC and that belonged to proteins which were encoded by genes located in a 500‐kb vicinity of our lead SNPs were selected for association analyses. The assignment of protein names (uniprot identifiers) to the corresponding genes was performed using the DAVID gene conversion tool (http://david.abcc.ncifcrf.gov/). Finally, after QC the following proteins measured in 197 SHIP‐TREND samples were available: ALS, CC121, IBP3, and RT34.

#### Lookups of top loci in association with IGF correlated traits

Top SNPs associated with levels of IGF‐I and IGFBP‐3 were examined in relationship to other phenotypes using published data on serum metabolites (Suhre *et al*., 2011; Shin *et al*., 2014), anthropometric traits (Heid *et al*., 2010; Lango Allen *et al*., 2010; Speliotes *et al*., 2010), bone mineral density (Estrada *et al*., 2012), diabetes (Voight *et al*., 2010; Morris *et al*., 2012) and glycemic traits (Dupuis *et al*., 2010; Saxena *et al*., 2010; Soranzo *et al*., 2010), coronary artery disease (Coronary Artery Disease Genetics C, 2011; Schunkert *et al*., 2011; Consortium CAD and Deloukas, 2013), and survival beyond 90 years (Broer *et al*., 2015). Detailed information of the published datasets used including its references is given in Table S9 (Supporting information).

#### Assessment of regulatory elements associated with identified loci

encode and roadmap data were assessed using haploreg (http://www.broadinstitute.org/mammals/haploreg/haploreg.php) and regulomedb (http://regulomedb.org/). Statistical analysis of individual Regulome scores was performed using Monte Carlo sampling of 10 SNPs (the size of our ‘observed data’ pool). RegulomeDB assigns scores to SNP loci based on the presence of histone marks, predicted and experimentally validated transcription factor binding, DNase hypersensitivity, and other evidence for regulatory function. Scores range from 1 to 7, with lower scores indicating stronger evidence for the presence of a regulatory element. For the purpose of this analysis, score subcategories (1a, 1b, etc.) were merged. A multinomial test was performed for a statistical comparison between the observed distribution and the background distribution. The LD plot in Fig. 3B was generated using haploview 4.2 with genetic data downloaded from version 3, Release 2, using the genomic region Chr1:109590000‐109630000, and analysis panel CEU + TSI. Histone mark and DNase tracks in Fig. 3C were downloaded from UCSC Genome Browser.

## Conflict of interest

The authors do not have conflict of interests.

## Supporting information


**Appendix S1**. Materials and methods.
**Fig. S1** Manhattan plots of men and women strata.
**Fig. S2** QQ plots of meta‐analysis results.
**Fig. S3** Regional association plots for IGF‐I traits.
**Fig. S4** Regional association plots for IGFBP‐3 traits.
**Fig. S5** Results of bivariate analysis of IGF‐I and IGFBP‐3.
**Fig. S6** Flow chart of the study's design.
**Table S1** Characteristics of study cohorts.
**Table S2** Assay and genotyping information of study cohorts.
**Table S3** GWAS results with *P*‐value <10^−6^ in stage 1.
**Table S4** Genome‐wide significant loci of bivariate analysis.
**Table S5** Sex interaction results of the 12 genome‐wide significant SNPs.
**Table S6** Results of eQTL lookup in the Muther dataset.
**Table S7** Results of gene expression analysis of the genome‐wide significant loci with IGF‐I and IGFBP‐3 in the SHIP‐TREND cohort.
**Table S8** Results of pQTL analysis in the SHIP‐TREND cohort.
**Table S9** Lookup of genome‐wide significant lead SNPs in IGF‐I/IGFBP‐3 correlated traits.
**Table S10** Lookup of Top IGF‐I associated SNPs in associations with survival beyond age 90 years old.
**Table S11** Lookup of Top IGFBP‐3 associated SNPs in associations with survival beyond age 90 years old.
**Table S12** Enrichment of putative regulatory elements among IGF‐1 and IGFBP‐3 associated loci.

**Data S1** Design and funding of participating cohort studies.
