# Erratum

**DOI:** 10.1111/acel.12612

**Published:** 2017-07-12

Teumer, A., Qi, Q., Nethander, M., Aschard, H., Bandinelli, S., Beekman, M., Berndt, S. I., Bidlingmaier, M., Broer, L., CHARGE Longevity Working Group, Cappola, A., Ceda, G. P., Chanock, S., Chen, M.‐H., Chen, T. C., Chen, Y.‐D. I., Chung, J., Del Greco Miglianico, F., Eriksson, J., Ferrucci, L., Friedrich, N., Gnewuch, C., Goodarzi, M. O., Grarup, N., Guo, T., Hammer, E., Hayes, R. B., Hicks, A. A., Hofman, A., Houwing‐Duistermaat, J. J., Hu, F., Hunter, D. J., Husemoen, L. L., Isaacs, A., Jacobs, K. B., Janssen, J. A. M. J. L., Jansson, J.‐O., Jehmlich, N., Johnson, S., Juul, A., Karlsson, M., Kilpelainen, T. O., Kovacs, P., Kraft, P., Li, C., Linneberg, A., Liu, Y., Loos, R. J. F., Body Composition Genetics Consortium, Lorentzon, M., Lu, Y., Maggio, M., Magi, R., Meigs, J., Mellström, D., Nauck, M., Newman, A. B., Pollak, M. N., Pramstaller, P. P., Prokopenko, I., Psaty, B. M., Reincke, M., Rimm, E. B., Rotter, J. I., Saint Pierre, A., Schurmann, C., Seshadri, S., Sjögren, K., Slagboom, P. E., Strickler, H. D., Stumvoll, M., Suh, Y., Sun, Q., Zhang, C., Svensson, J., Tanaka, T., Tare, A., Tönjes, A., Uh, H.‐W., van Duijn, C. M., van Heemst, D., Vandenput, L., Vasan, R. S., Völker, U., Willems, S. M., Ohlsson, C., Wallaschofski, H. and Kaplan, R. C. (2016), Genomewide meta‐analysis identifies loci associated with IGF‐I and IGFBP‐3 levels with impact on age‐related traits. Aging Cell, **15**: 811–824. https://doi.org/10.1111/acel.12490.

In the article, ‘Genomewide meta‐analysis identifies loci associated with IGF‐I and IGFBP‐3 levels with impact on age‐related traits’, the published Table 1 was incorrect, due to an error. The correct version of the table is shown below:

**Table 1:** Loci associated with IGF‐I and IGFBP‐3 concentrations in men and women combined samples at genomewide significance (P < 5×10^‐8^) after final stage

Trait	SNP	A1	A2	F1	*P*	I²	Chr	Position	Nearest Gene	Gene Distance	Direction effect	
											IGF‐I	IGFBP‐3
IGF‐I^a^	rs700753	C	G	0.35	1.60E‐23	4.2	7	46 720 209	*TNS3*	561 067	***−***	***−***
IGF‐I	rs780093	T	C	0.41	2.19E‐13	24.5	2	27 596 107	*GCKR*	0	***−***	+
IGF‐I	rs978458	T	C	0.26	1.56E‐10	0.0	12	101 326 369	*IGF1*	0	***+***	***−***
IGF‐I	rs2153960	A	G	0.69	5.16E‐09	22.5	6	109 082 339	*FOXO3*	0	***+***	+
IGF‐I	rs934073	C	G	0.71	6.48E‐09	21.8	2	25 790 669	*ASXL2*	25 087	***−***	***−***
IGF‐I	rs1065656	C	G	0.31	1.17E‐08	47.9	16	1 778 837	*NUBP2*	0	***−***	***−***
IGF‐I	rs509035	A	G	0.31	2.09E‐08	0.0	3	173 646 143	*GHSR*	0	***+***	+
IGFBP‐3^a^	rs11977526	A	G	0.41	4.16E‐161	51.5	7	45 974 635	*IGFBP3*	47 239	***−***	***+***
IGFBP‐3^a^	rs700753	C	G	0.35	1.11E‐46	26.7	7	46 720 209	*TNS3*	56 1067	***−***	***−***
IGFBP‐3^a^	rs1065656	C	G	0.31	8.55E‐23	24.1	16	1 778 837	*NUBP2*	0	***−***	***−***
IGFBP‐3^a^	rs4234798	T	G	0.39	8.86E‐19	0.0	4	7 270 834	*SORCS2*	0	***−***	***−***
Bivariate analysis	rs646776	T	C	0.78	6.87E‐9	26.1/43.1	1	109 620 053	*CELSR2*	152	***−***	+

a Known association; ‘‐’, coding allele associated with lower IGF‐1 and IGFBP‐3 levels (indicated by bold italicized text were genomewide significant); “+”, coding allele associated with higher IGF‐1 and IGFBP‐3 levels (indicated by bold italicized text were genomewide significant); Chr, chromosome; A1, coding allele; A2, other allele; F1, frequency of coding allele.

We apologize for the inconvenience caused.

