# Supplementary material for: Genomewide meta‐analysis identifies loci associated with IGF‐I and IGFBP‐3 levels with impact on age‐related traits
Source: Aging Cell. 2016 Jun 21;15(5):811–24. doi: 10.1111/acel.12490 (PMC5013013; doi:10.1111/acel.12490)
Supplement: Supplementary file 1 — Appendix S1. Materials and methods. Fig. S1 Manhattan plots of men and women strata. Fig. S2 QQ plots of meta‐analysis results. Fig. S3 Regional association plots for IGF‐I traits. Fig. S4 Regional association plots for IGFBP‐3 traits. Fig. S5 Results of bivariate analysis of IGF‐I and IGFBP‐3. Fig. S6 Flow chart of the study's design. Table S1 Characteristics of study cohorts. Table S2 Assay and genotyping information of study cohorts. Table S3 GWAS results with P‐value <10−6 in stage 1. Table S4 Genome‐wide significant loci of bivariate analysis. Table S5 Sex interaction results of the 12 genome‐wide significant SNPs. Table S6 Results of eQTL lookup in the Muther dataset. Table S7 Results of gene expression analysis of the genome‐wide significant loci with IGF‐I and IGFBP‐3 in the SHIP‐TREND cohort. Table S8 Results of pQTL analysis in the SHIP‐TREND cohort. Table S9 Lookup of genome‐wide significant lead SNPs in IGF‐I/IGFBP‐3 correlated traits. Table S10 Lookup of Top IGF‐I associated SNPs in associations with survival beyond age 90 years old. Table S11 Lookup of Top IGFBP‐3 associated SNPs in associations with survival beyond age 90 years old. Table S12 Enrichment of putative regulatory elements among IGF‐1 and IGFBP‐3 associated loci. [file ACEL-15-811-s001.pdf]

Genome-wide meta-analysis reveals loci associated with circulating levels of IGF-I and IGFBP-3 with implications for metabolic and age related traits

SUPPLEMENTAL MATERIALS

Table of Contents

MATERIALS AND METHODS 3

Meta-Analysis 3

Assessment of Independent Signals 4

Gene Expression and eQTL Analyses 5

Association with Plasma Protein Levels 5

Lookups of Top Loci in Association with metabolites and IGF correlated Traits 8

SUPPLEMENTARY FIGURES 10

Supplementary Figure 1: Manhattan Plots of Men and Women Strata 10

Supplementary Figure 2: QQ Plots of Meta-Analysis Results 12

Supplementary Figure 3: Regional Association Plots for IGF-I Traits 13

Supplementary Figure 4: Regional Association Plots for IGFBP-3 Traits 16

Supplementary Figure 5: Results of Bivariate Analysis of IGF-I and IGFBP-3 17

Supplementary Figure 6: Flow chart of the study's design 19

SUPPLEMENTARY TABLES 20

Supplementary Table 1: Characteristics of study cohorts 20

Supplementary Table 2: Assay and genotyping information of study cohorts 22

Supplementary Table 3: GWAS results with p-value < 10^{-6} in stage 1 24

Supplementary Table 4: Genome-wide significant loci of bivariate analysis 25

Supplementary Table 5: Sex interaction results of the 12 genome-wide significant SNPs 26

Supplementary Table 6: Results of eQTL lookup in the Muther dataset 27

Supplementary Table 7: Results of gene expressison analysis of the genome-wide significant loci with IGF-I and IGFBP-3 in the SHIP-TREND cohort 35

Supplementary Table 8: Results of pQTL analysis in the SHIP-TREND cohort 43

Supplementary Table 9: Lookup of genome-wide significant lead SNPs in IGF-I/IGFBP-3 correlated traits 45

Supplementary Table 10: Lookup of Top IGF-I associated SNPs in associations with survival beyond age 90 years old 47

Supplementary Table 11: Lookup of Top IGFBP-3 associated SNPs in associations with survival beyond age 90 years old 55

Supplementary Table 12: Enrichment of putative regulatory elements among IGF-1 and IGFBP-3 associated loci 70

REFERENCES 71

Materials and Methods

Meta-Analysis

Individual studies' result files underwent extensive quality control before meta-analysis. File format as well as plausibility and distributions of association results including effects, their standard errors, allele frequency and imputation quality of the SNPs were performed by the gwasqc() function of the GWAtoolbox package v1.01(Fuchsberger *et al.* 2012). All cohort specific λ_{GC} values were between 0.99 and 1.07 for the IGFBP-3 and between 0.98 and 1.08 for the IGF-I outcome. Additionally, the known IGFBP-3 association of rs11977526 in *IGFBP3* was checked for a consistent effect direction and size in each study. The family-based cohorts FHS and MICROS conducted additional analyses on the men and women combined sample by additionally adjusting for sex to account for relatedness in the sex-combined analyses.

In total, GWAS results of ~2.6 million SNPs in at least 50% of the studies were available. Genome-wide significance was defined as a p-value < 5×10^{-8} correcting for one million independent tests.

Because several studies joined the project after the initial GWAS was finished, we implemented a multi-stage design with two GWAS stages and an additional stage with *de novo* genotyping data to confirm novel loci associated with (IGF-I only) levels. After stage 1 GWAS, all 19 lead SNPs from all traits with a P < 10^{-6} were taken forward to stage 2. SNPs were selected only once if the smallest P-value was in the overall stratum without adjustment for IGF-I or IGFBP-3, respectively, and if the association had the same effect direction after this adjustment or in the respective sex-stratum. One additional cohort with IGF-I but without genome-wide

data was available for stage 3. Therefore, all IGF-I lead SNPs of novel loci that had a combined stage 1 and stage 2 $P < 10^{-8}$ (except *GCKR*) were selected for *de novo* replication. The lead SNP rs780093 of the *GCKR* locus was already genome-wide significant in stage 1 ($P= 1.0×10^{-9}$) and had a P-value of $4.8×10^{-6}$ in stage 2 which is significant after Bonferroni correction for 19 tests, and thus was not included in the *de novo* replication due to limited funding. Of the seven SNPs selected for replication, for one SNP (rs1065656 of the *NUBP2* locus) *de novo* genotyping failed. All lead SNPs that were genome-wide significant after the final stage were considered as replicated.

From among all SNPs not being in linkage equilibrium (see Methods) and where results were available from at least 50% of the studies, the SNP with the smallest P-value was selected as lead SNP for the respective locus and trait. Of note, the combined stage 1 and stage 2 GWAS meta-analysis did not reveal any additional loci with a P-value $< 10^{-8}$. However, if there was a SNP in high LD with a lead SNP and also having a smaller p-value than the lead SNP after this combined analysis, this SNP was selected as the new lead SNP of the respective locus.

Assessment of Independent Signals

The analysis of secondary signals in the *NUBP2* locus was performed using the software GCTA (Yang *et al.* 2011) and the genotypes of the SHIP cohort, and was verified by a second analysis using the genotypes of the NHS/HPFS cohorts. The GC-corrected and QC filtered meta-analysis results and a condition list containing the lead SNPs of the final loci were used as input for the conditional analysis. An additional hit was found if the conditional P-value was below genome-wide significance. Subsequently, this SNP was added to the condition list and the

conditional analysis was performed again until no additional significant independent association was found.

Gene Expression and eQTL Analyses

For each of the lead SNP of the significant loci after final stage, significant cis eQTL associations in whole blood were looked-up in the publically available association result database (Westra *et al.* 2013). We also looked-up cis eQTL associations for lead SNPs in "MuTHER" (http://www.muther.ac.uk/) (Grundberg *et al.* 2012). Lymphocytes (LCLs and in some, fresh lymphocytes); subcutaneous fat; muscle and skin biopsies have been obtained from ~856 twins (1/3 monozygotic, 2/3 dizygotic) from the well-characterised TwinsUK BioResource.

Association analysis of whole blood gene expression data with serum IGF-I and IGFBP-3 measurements was conducted in 986 samples of the SHIP-TREND cohort. The gene expression levels were obtained and normalized as described previously (Schurmann *et al.* 2012). Gene expression levels were used as outcome in a linear regression model including the serum IGF-I or IGFBP-3 values as independent variable by adjusting for sex, age, red and white blood cell counts, RNA integrity number, sample storage time, and RNA amplification and labeling batch as a factor. For sensitivity analyses, BMI was included as an additional covariate in the model. The SHIP-TREND expression dataset is available at GEO (Gene Expression Omnibus) public repository under the accession GSE 36382.

Association with Plasma Protein Levels

The complexity of the plasma proteome presents an analytical challenge. Therefore, we subjected the plasma samples to immunoaffinity subtraction using the Multiple Affinity

Removal System (MARS) (Agilent Technologies, Wilmington, DE, USA) coupled to a ProteomeLab PF 2D system (Beckman Coulter, Brea, CA, USA). In brief, 20 µL plasma was diluted with 60 µL buffer A, transferred to a 0.22 µm spin filter and centrifuged for 1 min at 16,000×g to remove crude particles. The supernatant was loaded on the MARS-6 column (4.6 x 50 mm) for the depletion of the TOP-6 plasma proteins. The flow through with the low-abundant proteins was collected using the fraction collector and immediately stored at -80°C. The depleted plasma samples were sequentially incubated in the presence of dithiothreitol (0.12% w/v) at 37°C for 30 min and precipitated using trichloroacetic acid (TCA) at a final concentration of 10% (v/v). Solubilized protein was determined using a Bradford assay with bovine serum albumin as standard (Bio-Rad, Hercules, CA, USA).

Protein lysates were investigated by liquid chromatography mass spectrometry. In total, 4 µg protein lysates were reduced (2.5 mM DTT for 1 h at 60°C) and alkylated (10 mM iodacetamide for 30 min at 37°C). Proteolysis was performed overnight using trypsin (Promega, Madison, WI, USA) with a ratio of 1:25 at 37°C. The tryptic digestion was stopped by adding acetic acid at the final concentration of 1%, followed by desalting and purification using ZipTip-µC18 tips (Millipore, Billerica, MA, USA). Large scale proteomics requires, firstly, a randomized/blinded samples preparation to reduce the introduction of technical variance, secondly, divide the whole sample cohort into similar-numbered MS measuring blocks (= “batch”) to later bioinformatically adjust the technical variance introduced by LC-MS analysis in order to ensure routinely MS service, maintenance and calibration procedures during the MS sample acquisition.

Proteolytically cleaved peptides were separated prior to mass spectrometric analyses by reverse phase nanoACQUITY UPLC system (Waters, Milford, MA, USA) on a BEH130 C18 column (10 cm length, 100 µm inner diameter and 1.7 µm particle size, Waters) at a constant flow rate of 400 nL/min. Separation was achieved using a non-linear gradient of buffer B from 2% up to 60% within 90 min with 0.1% acetic acid, 2% acetonitrile in water (solvent A) and 0.1% acetic acid in 100% acetonitrile (solvent B). Separated peptides were monitored using an LTQ Orbitrap Velos MS (Thermo Scientific) equipped with a nano electrospray ion source operated with PicoTip Emitters (New Objective, Woburn, MA, USA). The MS instrument was operated in data-dependent MS/MS mode, after a first survey scan (r=30,000) MS/MS data were recorded for the Top-20 peaks in the ion trap at a collision induced energy (CID) of 35%. The exclusion time to reject masses from repetitive MS/MS fragmentation was set to 60 sec and the minimal ion signal for MS/MS was 2,000.

Raw MS data were processed using the Refiner MS 7.5 and Analyst 7.5 module (Genedata, Basel, Switzerland). MASCOT (in-house Mascot server v2.3.2, Matrix Science, London, GB) search algorithm was used to match the generated peak lists with a human FASTA-formatted database containing 20,268 unique sequence entries (reviewed human database, release of Oct/2011). Database searches were performed with carbamidomethyl (Cys) as fixed modification and oxidation (Met) as variable modification. Enzyme specificity was selected to trypsin with up to two missed cleavages allowed using 10 ppm MS tolerance and 0.6 Da MS/MS tolerances. We accepted only ranked 1 peptide hits with a Mascot ion score >23 as identified.

Prior to analyses, all peptide intensity values were median-median normalized. Association analyses with serum IGF-I and IGFBP-3 levels were performed by linear regression using the serum levels as outcomes, and including the peptide intensity, sex, age and the MS processing batch as exposures. Associations of a SNP with the peptides were conducted by linear regression using the SNP, sex, age and the first four principal components of a peptide level based principal component analysis as independent variables. Protein intensities used for analyses were obtained by averaging the corresponding peptide intensities that passed the QC filter, and were put instead of the peptide intensities into the association model.

All measured peptides that passed QC of proteins that were encoded by genes located in a 500kb vicinity of our lead SNPs were selected for association analyses. The assignment of protein names (uniprot identifiers) to the corresponding genes was performed using the DAVID gene conversion tool (http://david.abcc.ncifcrf.gov/). Finally, for the following proteins peptide intensities measured in 197 SHIP-TREND samples were available for analyses after QC: ALS, CC121, IBP3 and RT34.

Lookups of Top Loci in Association with metabolites and IGF correlated Traits

Serum metabolites: publicly available data database of the Metabolomics GWAS Server (http://www.gwas.eu) (Suhre *et al.* 2011; Shin *et al.* 2014).

Anthropometric traits: publically available data of the GIANT Consortium (http://www.broadinstitute.org/collaboration/giant/index.php/Data_Release).(Heid *et al.* 2010; Lango Allen *et al.* 2010; Speliotes *et al.* 2010) Results for fat percentage were obtained from a GWAS of fat percentage provided by Ruth Loos *et al*.

Bone mineral density: publicly available data of the GEFOS Consortium (http://www.gefos.org/?q=content/data-release).(Estrada *et al.* 2012)

Type 2 diabetes: publicly available data of the DIAGRAM Consortium (http://diagram-consortium.org/downloads.html).(Voight *et al.* 2010; Morris *et al.* 2012)

Diabetes related traits: publicly available data of the MAGIC Consortium (http://www.magicinvestigators.org/downloads/).(Dupuis *et al.* 2010; Saxena *et al.* 2010; Soranzo *et al.* 2010)

Coronary artery disease: publicly available data of the CARDIoGRAMplusC4D Consortium (http://www.cardiogramplusc4d.org/downloads/).(Coronary Artery Disease Genetics 2011; Schunkert *et al.* 2011; Consortium *et al.* 2013)

Survival beyond age 90 years old: results were obtained from a GWAS of longevity providing by Linda Broer *et al* (Broer *et al.* 2014).

Supplementary Figures

Supplementary Figure 1: Manhattan Plots of Men and Women Strata

Manhattan plots of the combined stage 1 and 2 meta-analysis results of IGF-I and IGFBP-3 traits in men and women strata. SNPs are plotted on the x-axis according to their position on each chromosome with the $-\log_{10}$ association p-value on the y-axis. The upper solid horizontal line indicates the threshold for genome-wide significance. Known hits are colored in orange, new findings in blue. Plots are truncated on the y-axis to 20.

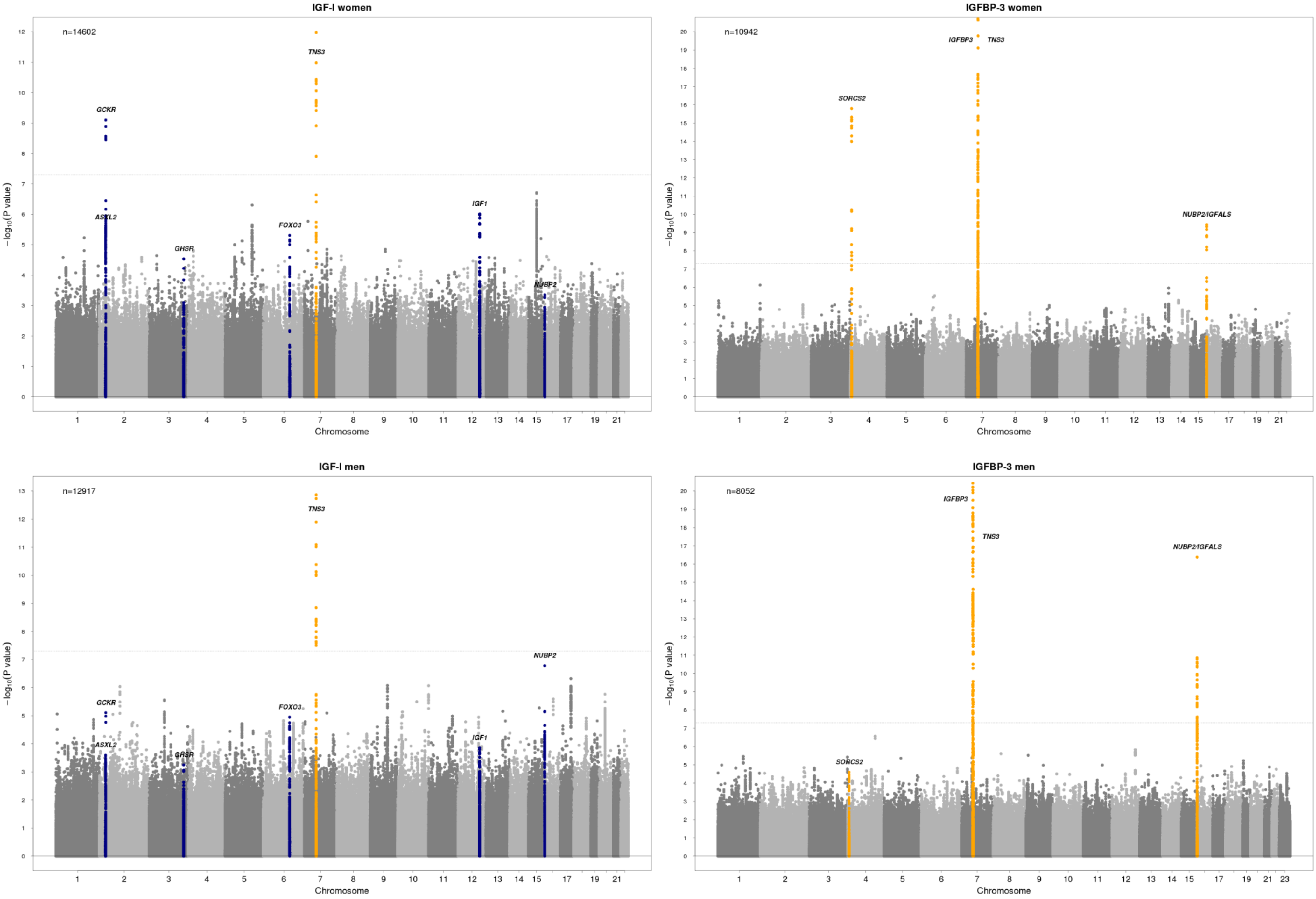
IGF-I women
n=14602
TNS3
GCKR
ASXL2
GHSR
FOXO3
IGF1
NUBP2
IGFBP-3 women
n=10942
IGFBP3
TNS3
SORCS2
NUBP2/IGFALS
IGF-I men
n=12917
TNS3
NUBP2
GCKR
FOXO3
ASXL2
GHSR
IGF1
IGFBP-3 men
n=8052
IGFBP3
TNS3
NUBP2/IGFALS
SORCS2
−log10(P value)
Chromosome

Supplementary Figure 2: QQ Plots of Meta-Analysis Results

Quantile-quantile plots of the combined stage 1 and 2 meta-analysis results of the IGF-I and IGFBP-3 traits across all strata. The observed p-values are plotted on the y-axis against the expected p-values under no association on the x-axis.

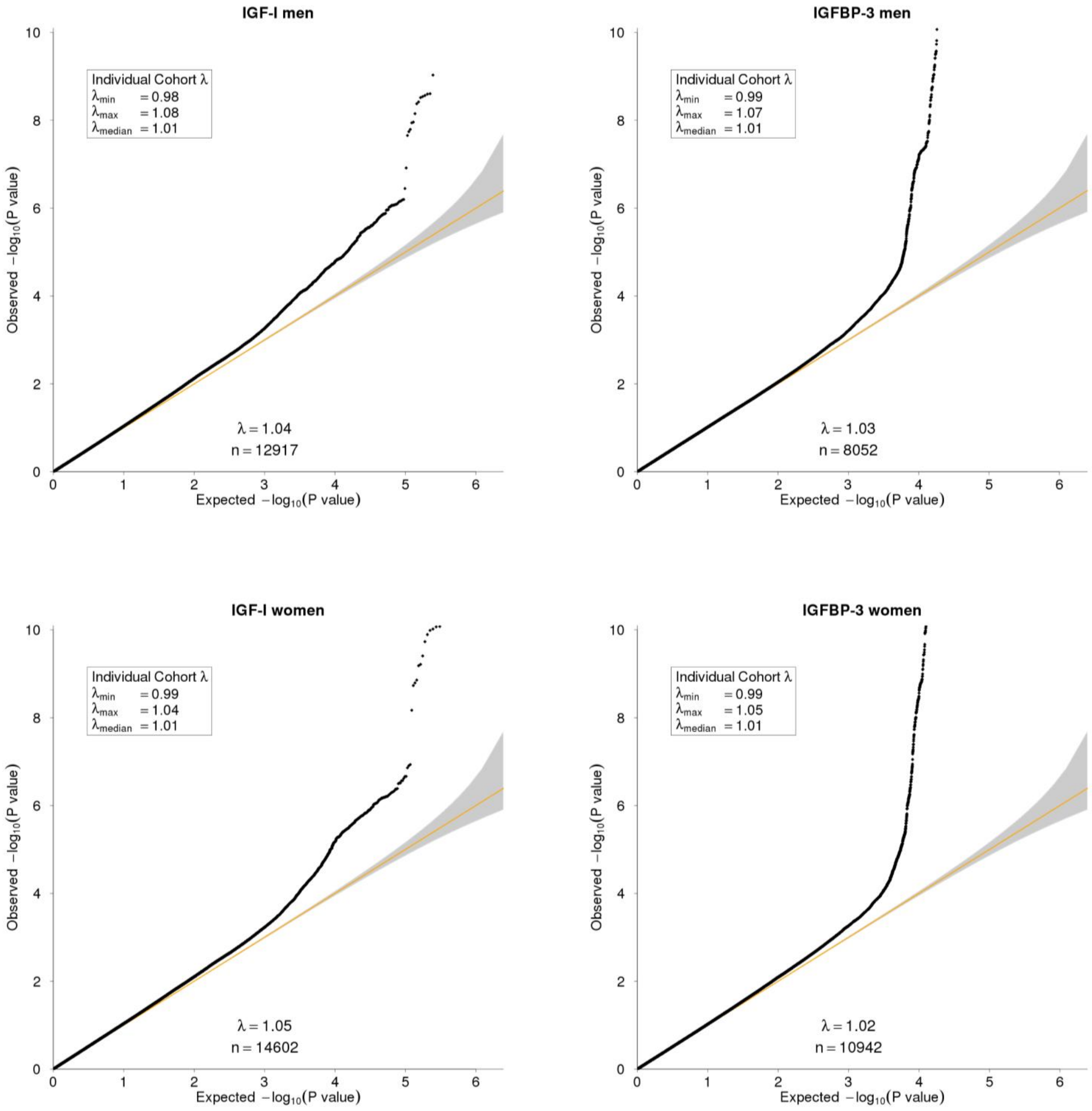

Supplementary Figure 3: Regional Association Plots for IGF-I Traits

Regional association plots of all genome-wide significantly associated SNPs with IGF-I as outcome after final stage. The $-\log_{10}$ association p-values of all SNPs in a 500kb vicinity are shown on the y-axis. The color corresponds to the correlation with the lead SNP based on the HapMap II CEU data. Plots are ordered by association p-value of the lead SNP.

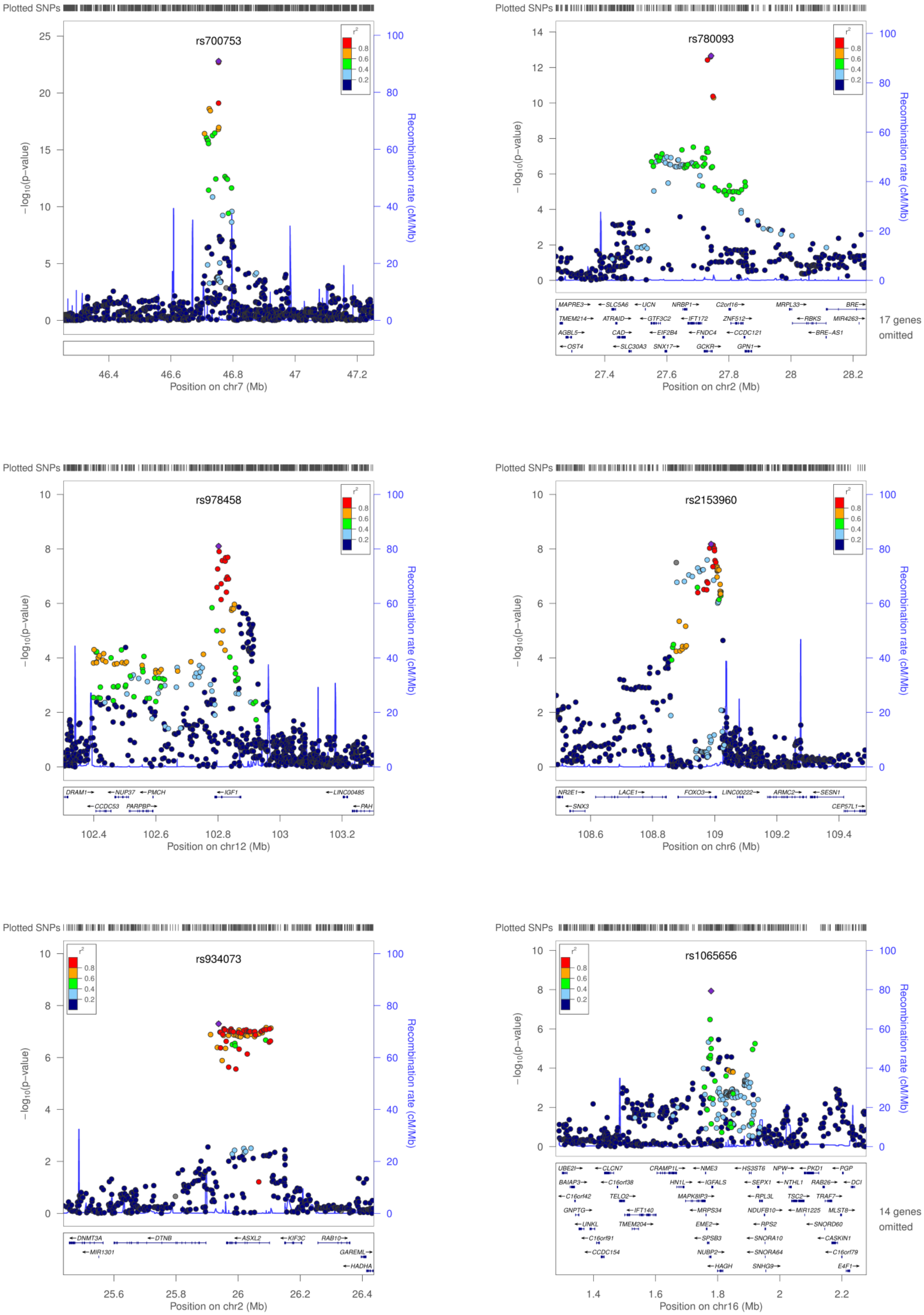
rs700753
Position on chr7 (Mb)
rs780093
Position on chr2 (Mb)
17 genes omitted
rs978458
Position on chr12 (Mb)
rs2153960
Position on chr6 (Mb)
rs934073
Position on chr2 (Mb)
rs1065656
Position on chr16 (Mb)
14 genes omitted
Plotted SNPs
−log10(p−value)
Recombination rate (cM/Mb)

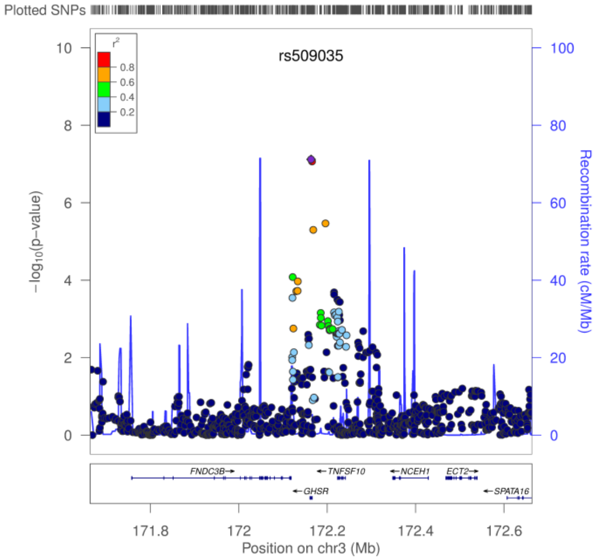

Plotted SNPs
rs509035
r2
0.8
0.6
0.4
0.2
−log10(p−value)
Recombination rate (cM/Mb)
FNDC3B
TNFSF10
NCEH1
ECT2
GHSR
SPATA16
171.8
172
172.2
172.4
172.6
Position on chr3 (Mb)

Supplementary Figure 4: Regional Association Plots for IGFBP-3 Traits

Regional association plots of all genome-wide significantly associated SNPs with IGFBP-3 as outcome after final stage. The -$\log_{10}$ association p-values of all SNPs in a 500kb vicinity are shown on the y-axis. The color corresponds to the correlation with the lead SNP based on the HapMap II CEU data. Plots are ordered by association p-value of the lead SNP.

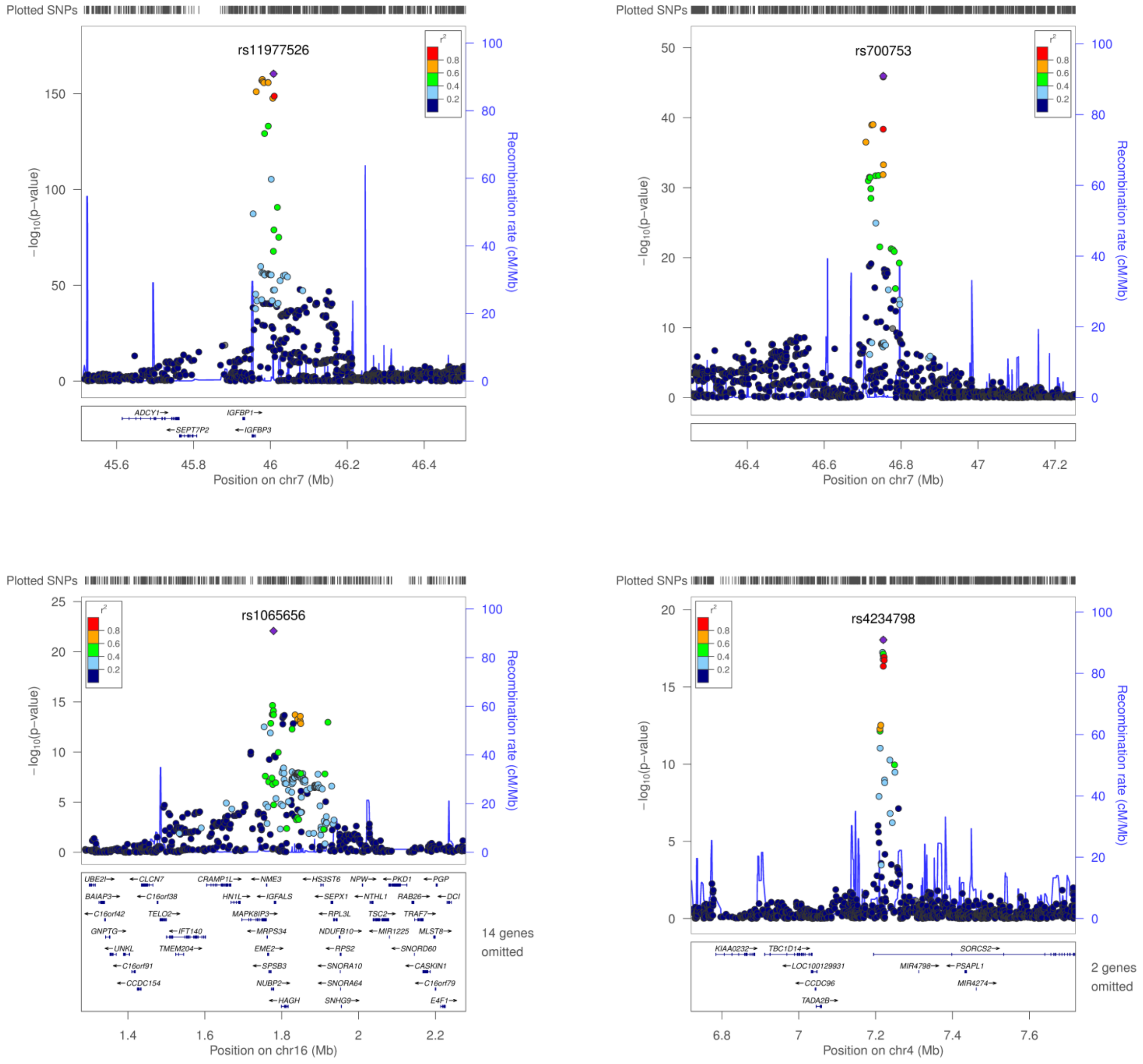

Supplementary Figure 5: Results of Bivariate Analysis of IGF-I and IGFBP-3

Manhattan plot (upper panel) and quantile-quantile plot (lower panel) of the genome-wide bivariate analysis on IGF-I and IGFBP-3. In the Manhattan plot, SNPs are plotted on the x-axis according to their position on each chromosome with the $-\log_{10}$ association p-value on the y-axis. The upper solid horizontal line indicates the threshold for genome-wide significance. Genome-wide significant loci are colored in blue. The plot is truncated on the y-axis to 20. The quantile-quantile plot shows the observed p-values on the y-axis against the expected p-values under no association on the x-axis.

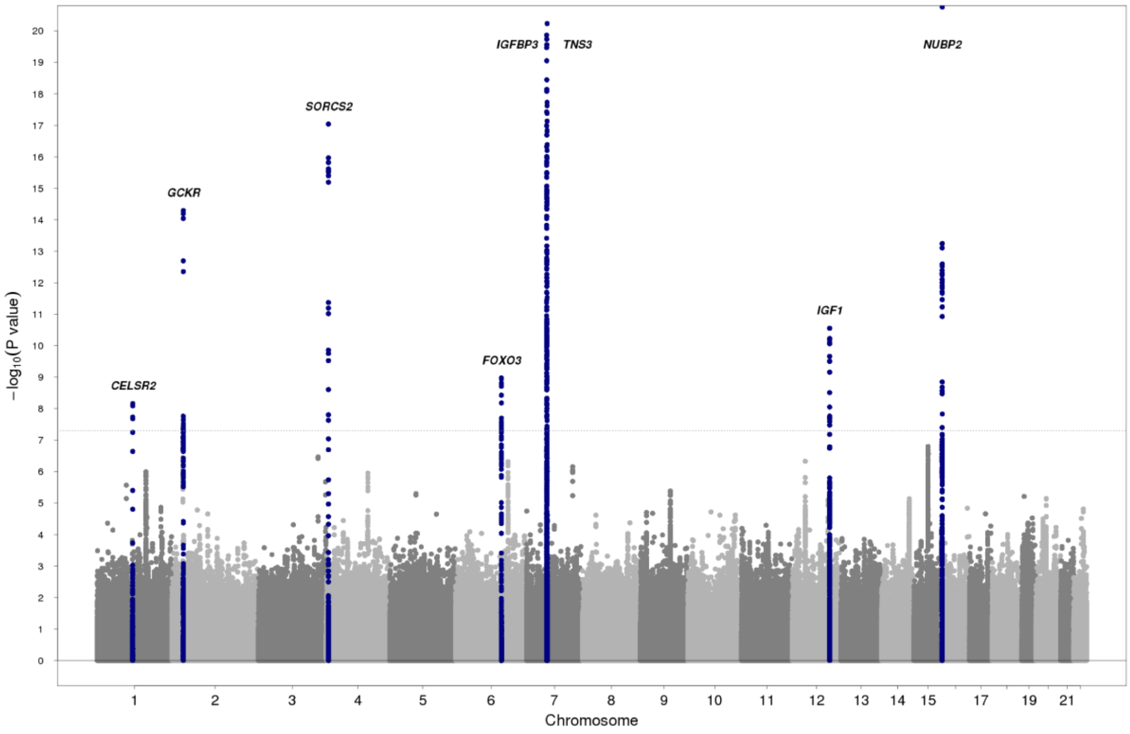

IGFBP3
TNS3
NUBP2
SORCS2
GCKR
IGF1
FOXO3
CELSR2
−log10(P value)
Chromosome

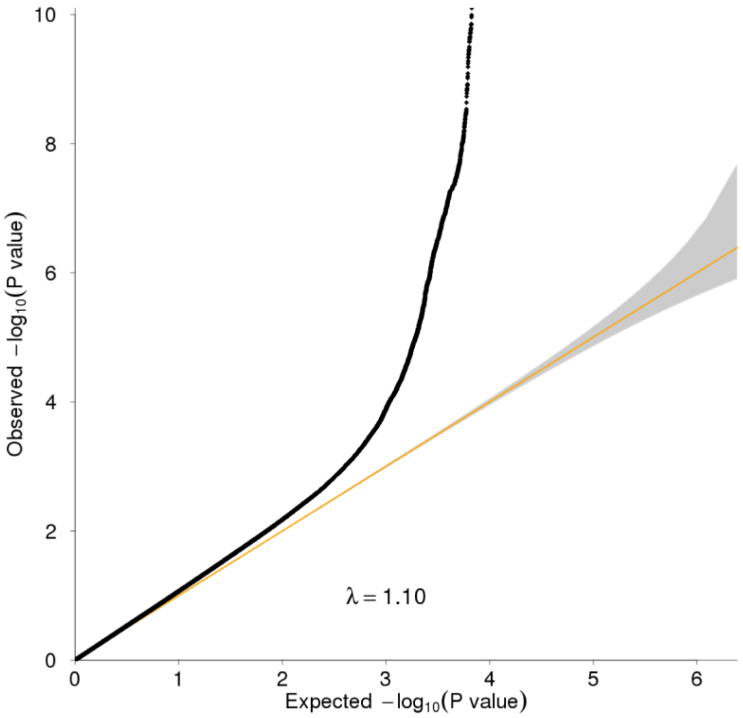

Observed −log10(P value)
Expected −log10(P value)
λ = 1.10

Supplementary Figure 6: Flow chart of the study's design.

Loci significantly associated with circulating IGF-I and IGFBP-3 concentrations at each analysis stage.

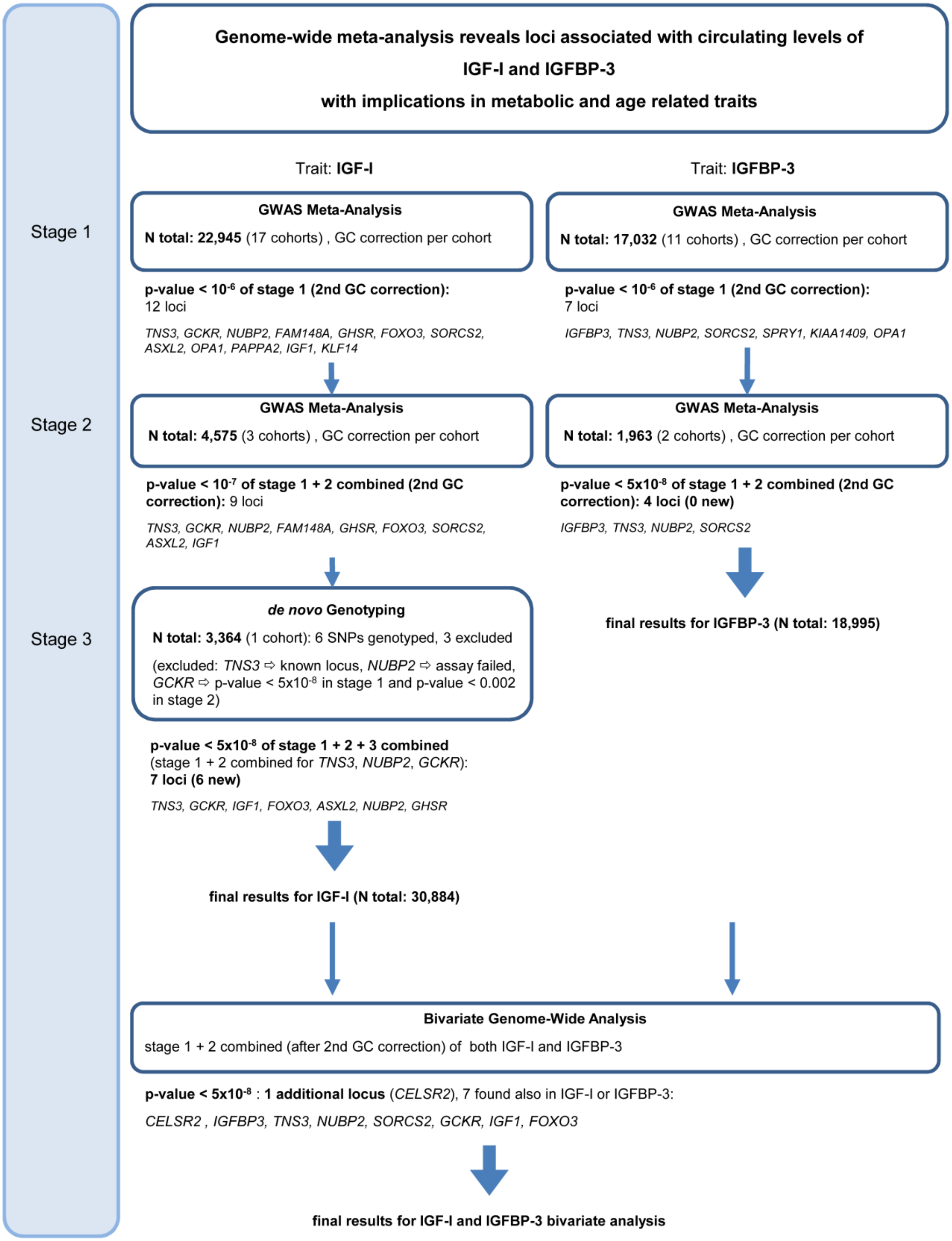

Supplementary Tables

Supplementary Table 1: Characteristics of study cohorts

Study	Stage	N IGF-I	N IGFBP-3	% Male	Mean Age	IGF-I [ng/ml], median (IQR) or mean± SD	IGFBP-3 [ng/ml], median (IQR) or mean± SD	Study Region	Reference, cohort study design
BLSA	1	516	*- 0 -*	55	68	120 (90, 153)	NA	USA	Shock, N.W., et al., Normal Human Aging: The Baltimore Study of Aging. 1984
CHS*	1 / 2	2615*	2815*	39	76	153.3 (115.9, 195.9)	3846 (3261, 4474)	USA	Fried, LP et al, Ann Epidemiol 1991 Feb;1(3):263-76 PMID: 1669507
FHS	1	3600	3600	47	40	123.53 (100.60, 151.10)	2907.57 (2238.38, 3761.41)	USA	Dawber et al, Am J Public Health Nations Health 1951;41:279-81.
GOOD	1	938	*- 0 -*	100	18.9	253.2 (215.7, 305.9)	NA	Sweden	[PMID: 16007330] Lorentzon et al. 2005. J Bone Miner Res 20, 1334-1341.
HABC	1	339	88	53	74	111 (86, 138)	3436 (2925, 4088)	USA	Cesari M et al. Am J Cardiol , 2003, 92, 522–528. PMID:12943870
InCHIANTI	1	1172	*- 0 -*	43	68	119.1 (83.9, 163.3)	NA	Italy	Ferrucci L et al, J Am Geriatr Soc. 2000 Dec;48(12):1618-25 PMID: 11129752
KORA	1	1671	1655	48	60	128.0 (101.0, 157.0)	3405 (2870, 3972)	Germany	Wichmann et al, Gesundheitswesen 2005;67:26-30
LLS	1	1910	1910	45	59	131.58 ± 37.5	4400 ± 900	The Netherlands	Westendorp et al. 2009 J Am Geriatr Soc. Sep;57(9):1634-7 doi: 10.1111/j.1532-5415.2009.02381.x
MICROS	1	1189	*- 0 -*	47	46	149.0 (84)	NA	Italy	Pattaro el al (2007), BMC Med Gen 5, 8-29
MrOS Sweden	1	941	*- 0 -*	100	75	113.1 (85.2, 145.8)	NA	Sweden	[PMID: 16598372] Mellström et al. 2006 J Bone Miner Res 21, 529-535.
NHS.CGEM	1	1240	1239	0	57	148.0 (115.0, 191.4)	4531.7 (3832.95, 5279.37)	USA	Hankinson SE et al. Lancet 1998;351: 1393-6.
NHS.CHD	1	549	549	0	60	see above	see above	USA	Hart JE et al. Circulation 2014: 130(17):1474-82. PMID: 25332277
NHS.T2D	1	1032	1032	0	56	see above	see above	USA	Rajpathak SN et al, Diabetes 2012;61:2248-54.
PLCO	1	1080	1079	100	64	193.9 (145.7, 256.4)	4526.4 (3831.3, 5236.8)	USA	Weiss JM et al. Int J Cancer 2007; Prorok et al. Control Clin Trials 200; Hayes et al. Mutat Res 2005;Yeager et al. Nat Genet 2007

RS	1	158	158	49	67	136.17	3818.8 (914.7)	The Netherlands	Hofman et al, Eur J Epidemiol 7, 403-22 (1991); Hofman et al, Eur J Epidemiol 28, 889-926 (2013)
SHIP	1	3889	3889	49	50	133.4 (101.9, 170.8)	1892.6 (1565.6, 2202.4)	Germany	Völzke et al, Int J Epidemiology
SORBS	1	932	- *0* -	59	48		NA	Germany	PMID:21559053; PMID:22907691
FHS.G2E7	2	2768	- *0* -	45	61	144.6 ± 60	NA	USA	Neurology 2014;82:1613–1619
SHIP-Trend	2	981	981	44	50	138.5 (110.3 - 173.7)	4188 (3568 - 4825)	Germany	Völzke et al, Int J Epidemiology
Health2006	3	3364	- *0* -	55	49	150 (116 - 189)	NA	Denmark	Thuesen et al, Int J Epidemiol 2014 Apr;43(2):568-75

Stage 1, discovery stage; Stage 2, replication stage; Stage 3, de novo genotyping stage
* CHS study: for IGF-I, N=1789 stage-1 and N= 826 stage-2, and for IGFBP-3, N=1833 stage-1 and N=982 stage-2.

Supplementary Table 2: Assay and genotyping information of study cohorts

Study	Specimen type	IGF-I assay	IGFBP-3 assay	Genotyping array type	Genotype calling	Genotype imputation
BLSA	Fasting, serum	Automated enzymaticic chemiluminescent assay, Pacific Biomarkers, Seattle, WA, USA	N/A	Illumina 550K	Beadstudio	MACH
CHS	Fasting, plasma	Stage 1: ELISA, Immunodiagnostic Systems Ltd/IDS, Boldon Business Park, Boldon, Tyne & Wear, England. Stage 2: ELISA, Diagnostics Systems Laboratory/DSL, Webster, TX, USA	Stage 1: Sandwich chemiluminescence assay, Immunodiagnostic Systems Ltd/IDS, Boldon, Tyne & Wear, England. Stage 2: ELISA, Diagnostics Systems Laboratory/DSL, Webster, TX, USA	Illumina 370CNV	Illumina BeadStudio	BIMBAM10 v0.99
FHS	Fasting, serum	ELISA, Human IGF-1 Immunoassay DG100, R&D Systems	ELISA, Human IGFBP-3 Immunoassay DGB300, R&D Systems	Affymetrix 550K	BRLMM	MACH
GOOD	Non-fasting, serum	Double-antibody IGF-binding protein-blocked RIA, Mediagnost, Tubingen, Germany	N/A	Illumina HumanHap 610K	BeadStudio	MACH
HABC	Fasting, serum	IGF-binding protein-blocked radioimmunoassay, ALPCO, Windham, NH, USA	Coated tube immunoradiometric (IRMA) kit, Diagnostic Systems Laboratory, Webster, TX), USA	Illumina Human1M-Duo	BeadStudio	MACH version 2.0.16
InCHIANTI	Fasting, serum	Immunoradiometric assay, Diagnostic System Laboratories, Milan, Italy.	N/A	Illumina 550K	Beadstudio	MACH
KORA	Fasting, EDTA plasma	Chemiluminescent immunometric assay, Immulite 2000, Siemens	Chemiluminescent immunometric assay, Siemens Immulite 2500	Affymetrix 6.0	Birdseed2	IMPUTE v0.4.2
LLS	Non-fasting, serum	Immulite 2500, DPC, Los Angeles, CA, USA	Immulite 2500, DPC, Los Angeles, CA, USA	Illumina 660W-Quad, Illumina OmniExpress	GenomeStudio	IMPUTEv2
MICROS	Fasting, serum	Chemiluminescent immunometric assay IMMULITE® 2000 analyzer, Siemens	N/A	Illumina HumHap300	Bead Studio	MACH v.1.016

MrOS Sweden	Fasting, serum	Double-antibody IGF-binding protein-blocked RIA, Mediagnost, Tubingen, Germany	N/A	Illumina Human1M-Duo/Illumina HumanOmni1-Quad	BeadStudio	MACH, minicmac
NHS.CGEM	64.4% fasting, 35.6% nonfasting, plasma	Enzyme-linked immunosorbent assay (ELISA), DSL, Webster, TX, USA	N/A	Affymetrix 6.0	Birdseed	MACH
NHS.CHD	62.7% fasting, 37.3% nonfasting, plasma	ELISA, Diagnostic Systems Laboratory/DSL, Webster, TX, USA	N/A	Affymetrix 6.0	Birdseed	MACH
NHS.T2D	94.7% fasting, 5.3% nonfasting, plasma	ELISA, Diagnostic Systems Laboratory/DSL, Webster, TX, USA	N/A	Illumina 550K	Birdseed	MACH
PLCO	Nonfasting blood (serum)	ELISA, Diagnostic Systems Laboratory/DSL, Webster, TX, USA	ELISA, Diagnostic Systems Laboratory/DSL, Webster, TX, USA	Illumina 240K+310K	Illumina Bead studio	MACH
RS	Fasting, serum	RIA, Medgenix Diagnosticss, Fleurus, Belgium	RIA, Medgenix Diagnosticss, Fleurus, Belgium	Illumina 550 and 610K	Beadstudio	MACH
SHIP	Non-fasting, serum	Automated two-site chemiluminescence immunoassay, Nichols Advantage	Automated two-site chemiluminescence immunoassay, Nichols Advantage	Affymetrix Genome-Wide SNP 6.0	Birdseed V2	IMPUTE v0.5.0
SORBS	Non-fasting, serum	Chemiluminescent immuno assay, Liasion, DiaSorin	N/A	Affymetrix 500K und Affymterix 6.0		IMPUTE
FHS.G2E7	Fasting, serum	Quantikine Human IGF-I DG100, SG100, and PDG100, R&D Systems, Minneapolis, MN, USA	N/A	Affymetrix GeneChipR Human Mapping 500K Array, 50K Human Gene Focused Panel	BRLMM	MACH
SHIP-Trend	Non-fasting, serum	Chemiluminescent immunometric assay, Immulite 2500, Siemens Healthcare Medical Diagnostics	Chemiluminescent immunometric assay, Immulite 2500, Siemens Healthcare Medical Diagnostics	Illumina Human Omni 2.5	GenCall	IMPUTE v2.1.2.3
Health2006	Fasting serum	Immulite 2000 IGF-1 assay, Siemens Medical Solutions Diagnostics, Los Angeles, CA, USA	N/A	N/A (replication de novo genotyping)	N/A	N/A

Supplementary Table 3: GWAS results with p-value < 10^{-6} in stage 1

Trait	SNP	Nearest Gene	Effect Allele	Other Allele	Effect Allele Frequency	N total stage 1	p-value stage1	N total stage 1+2	p-value stage 1+2	N total stage 1+2+3	p-value stage 1+2+3	p-value stage 2	N total stage 2	Median imputation quality	selected form *de novo* replication
IGF-I	rs700753	***TNS3***	c	g	0.35	22927	**3.70E-19**	27502	**1.60E-23**			**1.59E-06**	4575	1.00	no - known locus
IGF-I	rs780093	*GCKR*	t	c	0.41	22943	**1.02E-09**	27518	**2.19E-13**			**4.76E-06**	4575	1.00	no - significant in stage 2
IGF-I	rs1065656	*NUBP2*	c	g	0.31	22942	**2.23E-08**	27517	**1.17E-08**			1.08E-01	4575	0.91	yes, but genotyping failed
IGF-I	rs17205463	*FAM148A*	t	c	0.44	22942	8.47E-08	27517	**2.09E-08**	30876	7.88E-08	5.90E-02	4575	1.00	yes
IGF-I	rs509035	*GHSR*	a	g	0.31	22942	8.79E-08	27517	7.60E-08	30872	**2.09E-08**	1.78E-01	4575	1.00	yes
IGF-I	rs2153960*	*FOXO3***	a	g	0.69	22915	2.87E-07	27518	**6.63E-09**	30882	**5.16E-09**	3.40E-03	4575	0.99	yes
IGF-I	rs4234798	*SORCS2*	t	g	0.39	22941	6.50E-07	27516	8.38E-08	30870	1.75E-07	3.13E-02	4575	1.00	yes
IGF-I	rs934073	*ASXL2*	c	g	0.71	22944	7.82E-07	27519	5.03E-08	30867	**6.48E-09**	1.34E-02	4575	0.97	yes
IGF-I	rs2630239	*OPA1*	t	c	0.79	22944	8.06E-07	27519	5.16E-05			2.76E-01	4575	0.88	no - p > 1E-7
IGF-I	rs10489477	*PAPPA2*	t	c	0.15	22943	8.43E-07	27518	1.05E-07			2.93E-02	4575	0.99	no - p > 1E-7
IGF-I	rs978458	*IGF1*	t	c	0.26	22926	8.95E-07	27501	**7.92E-09**	30856	**1.56E-10**	**8.00E-04**	4575	1.00	yes
IGF-I	rs157931	*KLF14*	t	c	0.30	22920	9.68E-07	27495	2.32E-07			6.03E-02	4575	1.00	no - p > 1E-7
IGFBP-3	rs11977526	***IGFBP3***	a	g	0.41	17028	**5.98E-152**	18991	**4.16E-161**			**4.60E-12**	1963	0.96	no - known locus
IGFBP-3	rs700753	***TNS3***	c	g	0.35	17014	**4.52E-41**	18977	**1.11E-46**			**1.87E-07**	1963	1.00	no - known locus
IGFBP-3	rs1065656	***NUBP2***	c	g	0.31	17029	**4.73E-21**	18992	**8.55E-23**			3.78E-03	1963	0.93	no - known locus
IGFBP-3	rs4234798	***SORCS2***	t	g	0.39	17028	**3.05E-17**	18991	**8.86E-19**			6.96E-03	1963	1.00	no - known locus
IGFBP-3	rs17007484	*SPRY1*	a	c	0.88	17022	1.27E-07	18985	1.58E-07			4.43E-01	1963	0.99	no - p > 1E-7
IGFBP-3	rs943318	*KIAA1409*	a	g	0.67	17030	4.10E-07	18993	6.50E-07			5.69E-01	1963	0.96	no - p > 1E-7
IGFBP-3	rs2630239	*OPA1*	t	c	0.79	17031	6.88E-07	18994	4.17E-06			7.33E-01	1963	0.89	no - p > 1E-7

Significant p-values are marked in bold. All p-values except for stage 2 only results are corrected for 2nd genomic control.
* = lead SNP of locus after stage 1 was rs9480867 (p-value = 2.70E-07)
Genes of known associations are marked in bold (** = suggestive association)

Supplementary Table 4: Genome-wide significant loci of bivariate analysis

SNP	Effect Allele	Other Allele	Effect Allele Frequency	p-value	Chr	Position	Nearest Gene	Gene Distance
rs11977526	a	g	0.41	2.64E-195	7	45,974,635	*IGFBP3*	47239
rs700753	c	g	0.35	6.81E-48	7	46,720,209	*TNS3*	561067
rs1065656	c	g	0.31	1.76E-21	16	1,778,837	*NUBP2*	0
rs4234798	t	g	0.38	9.13E-18	4	7,270,834	*SORCS2*	0
rs1260326	t	c	0.42	5.15E-15	2	27,584,444	*GCKR*	0
rs2280496*	c	g	0.78	4.26E-12	7	45,723,522	*ADCY1**	0
rs978458	t	c	0.27	2.80E-11	12	101,326,369	*IGF1*	0
rs2153960	a	g	0.69	1.06E-09	6	109,094,877	*FOXO3*	0
rs788777*	a	g	0.06	1.47E-09	7	46,114,942	*IGFBP3**	187546
rs646776	t	c	0.78	6.87E-09	1	109,620,053	*CELSR2*	152

* = IGFBP3 locus

Supplementary Table 5: Sex interaction results of the 12 genome-wide significant SNPs

Trait	SNP	Effect Allele	Other Allele	p GxSEX	Chr	Position	Nearest Gene	Gene Distance	N (men)	z-score (men)	p (men)	N (women)	z-score (women)	p (women)
IGF-I	rs700753	c	g	8.71E-01	7	46,720,209	TNS3	561067	12911	-7.481	7.35E-14	14591	-7.251	4.15E-13
IGF-I	rs780093	t	c	2.24E-01	2	27,596,107	GCKR	0	12916	-4.456	8.36E-06	14601	-6.176	6.56E-10
IGF-I	rs978458	t	c	2.20E-01	12	101,326,369	IGF1	0	14413	3.652	2.61E-04	16443	5.388	7.11E-08
IGF-I	rs2153960	a	g	9.72E-01	6	109,082,339	FOXO3	0	14422	4.204	2.62E-05	16459	4.254	2.10E-05
IGF-I	rs934073	c	g	7.53E-01	2	25,790,669	ASXL2	25087	14414	-3.98	6.91E-05	16453	-4.425	9.62E-06
IGF-I	rs1065656	c	g	1.53E-01	16	1,778,837	NUBP2	0	12917	-5.291	1.21E-07	14601	-3.269	1.08E-03
IGF-I	rs509035	a	g	6.62E-01	3	173,646,143	GHSR	0	14418	3.599	3.19E-04	16454	4.218	2.47E-05
IGFBP-3	rs11977526	a	g	**4.96E-05**	7	45,974,635	IGFBP3	47239	8051	16.499	3.70E-61	10939	22.237	1.51E-109
IGFBP-3	rs700753	c	g	2.74E-02	7	46,720,209	TNS3	561067	8046	-8.635	5.86E-18	10931	-11.754	6.72E-32
IGFBP-3	rs1065656	c	g	6.24E-02	16	1,778,837	NUBP2	0	8052	-8.484	2.17E-17	10941	-5.849	4.95E-09
IGFBP-3	rs4234798	t	g	**3.48E-03**	4	7,270,834	SORCS2	0	8050	-4.17	3.05E-05	10941	-8.302	1.03E-16
Bivariate	rs646776	t	c	2.42E-01	1	109,620,053	CELSR2	152	7957	5.059	4.22E-07	10832	3.464	5.32E-04

p GxSEX: p-value from SNP-sex-interaction analysis.
Results are shown for the final stage.
Significant p-values after Bonferroni correction ($p<0.004$) are marked in bold.

Supplementary Table 6: Results of eQTL lookup in the Muther dataset

Illumina Probe	eQTL Gene	eQTL SNP	Effect Allele	Effect Allele Fre-quency	Fat beta	Fat SE	Fat p-value	LCL beta	LCL SE	LCL p-value	Skin beta	Skin SE	Skin p-value	GWAS locus
ILMN_1772731	**HAGH**	**rs1065656**	G	0.72	-0.106	0.015	**3.14E-12**	-0.172	0.016	**1.98E-26**	-0.039	0.013	**3.90E-03**	NUBP2
ILMN_2379520	**HAGH**	**rs1065656**	G	0.72	-0.092	0.016	**6.13E-09**	-0.126	0.014	**1.78E-20**	-0.045	0.014	**1.40E-03**	NUBP2
ILMN_1694593	**FAHD1**	**rs1065656**	G	0.72	-0.046	0.007	**1.05E-11**	-0.016	0.006	4.70E-03	-0.042	0.007	**2.12E-09**	NUBP2
ILMN_2246661	**FAHD1**	**rs1065656**	G	0.72	-0.036	0.016	2.01E-02	-0.075	0.013	**7.98E-09**	-0.078	0.021	2.06E-04	NUBP2
ILMN_1701457	**FAHD1**	**rs1065656**	G	0.72	-0.063	0.014	**1.35E-05**	-0.096	0.018	**1.31E-07**	-0.044	0.019	1.98E-02	NUBP2
ILMN_1671843	PSRC1	rs646776	T	0.78	-0.007	0.007	3.49E-01	-0.073	0.019	1.35E-04	-0.020	0.015	1.83E-01	CELSR2
ILMN_1729225	SORCS2	rs4234798	T	0.38	0.008	0.005	1.25E-01	-0.006	0.005	2.51E-01	0.031	0.009	3.62E-04	SORCS2
ILMN_1762255	GSTM1	rs646776	T	0.78	0.165	0.056	3.20E-03	0.251	0.073	6.26E-04	0.090	0.035	1.10E-02	CELSR2
ILMN_2356672	EIF2B4	rs780093	T	0.39	-0.005	0.012	7.01E-01	-0.027	0.008	1.10E-03	-0.042	0.013	1.40E-03	GCKR
ILMN_1745537	GNAT2	rs646776	T	0.78	-0.003	0.006	5.84E-01	0.026	0.008	1.70E-03	-0.010	0.006	1.30E-01	CELSR2
ILMN_1711208	CELSR2	rs646776	T	0.78	-0.008	0.006	1.76E-01	0.003	0.006	6.48E-01	-0.084	0.028	2.40E-03	CELSR2
ILMN_1687430	EIF2B4	rs780093	T	0.39	-0.019	0.012	1.18E-01	-0.016	0.009	7.98E-02	-0.041	0.014	2.90E-03	GCKR
ILMN_1746457	GTF3C2	rs780093	T	0.39	0.016	0.010	1.09E-01	-0.012	0.010	2.38E-01	0.042	0.015	3.60E-03	GCKR
ILMN_1741462	FAHD1	rs1065656	G	0.72	0.006	0.007	3.83E-01	-0.005	0.008	4.78E-01	-0.019	0.007	4.00E-03	NUBP2
ILMN_1682864	SPSB3	rs1065656	G	0.72	0.023	0.016	1.46E-01	0.044	0.015	4.10E-03	0.042	0.023	6.50E-02	NUBP2
ILMN_2188966	IGFALS	rs1065656	G	0.72	-0.007	0.006	2.04E-01	0.005	0.007	4.27E-01	-0.023	0.008	4.20E-03	NUBP2
ILMN_2210482	MRPS34	rs1065656	G	0.72	-0.016	0.010	1.05E-01	-0.043	0.016	7.40E-03	-0.013	0.013	3.15E-01	NUBP2
ILMN_1710416	HS3ST6	rs1065656	G	0.72	-0.010	0.005	5.70E-02	0.002	0.006	6.95E-01	0.065	0.024	7.60E-03	NUBP2
ILMN_1739521	NLGN1	rs509035	G	0.68	-0.033	0.012	7.80E-03	0.003	0.006	6.11E-01	0.009	0.010	3.44E-01	GHSR
ILMN_1687037	IFT140	rs1065656	G	0.72	0.016	0.008	4.29E-02	0.026	0.010	7.80E-03	-0.011	0.013	3.91E-01	NUBP2
ILMN_1747436	SCML4	rs2153960	G	0.29	-0.005	0.005	2.93E-01	-0.019	0.007	9.20E-03	-0.005	0.006	3.57E-01	FOXO3
ILMN_1659937	ZBTB24	rs2153960	G	0.29	0.011	0.010	2.70E-01	0.028	0.011	9.60E-03	-0.017	0.013	1.89E-01	FOXO3
ILMN_1713884	C16orf42	rs1065656	G	0.72	-0.004	0.016	8.21E-01	-0.039	0.015	9.60E-03	-0.009	0.018	6.23E-01	NUBP2
ILMN_1791217	FAM40A	rs646776	T	0.78	0.013	0.011	2.31E-01	-0.021	0.012	8.69E-02	0.037	0.015	1.08E-02	CELSR2
ILMN_2173835	FTHL3	rs780093	T	0.39	0.022	0.027	4.19E-01	0.020	0.026	4.29E-01	0.039	0.015	1.19E-02	GCKR
ILMN_2055165	MRFAP1	rs4234798	T	0.38	0.024	0.014	8.04E-02	0.002	0.010	8.37E-01	-0.045	0.018	1.26E-02	SORCS2
ILMN_1754241	C16orf73	rs1065656	G	0.72	-0.005	0.006	3.60E-01	0.009	0.006	1.58E-01	0.019	0.008	1.26E-02	NUBP2
ILMN_2057566	PGP	rs1065656	G	0.72	0.037	0.015	1.35E-02	-0.021	0.018	2.42E-01	0.035	0.017	4.42E-02	NUBP2
ILMN_1806867	PPM1G	rs780093	T	0.39	0.013	0.012	2.43E-01	-0.004	0.014	7.80E-01	-0.034	0.014	1.38E-02	GCKR
ILMN_1775549	PRSS27	rs1065656	G	0.72	0.009	0.007	1.83E-01	0.012	0.008	1.25E-01	0.026	0.011	1.43E-02	NUBP2
ILMN_1670096	NRBP1	rs780093	T	0.39	0.019	0.012	1.15E-01	0.031	0.013	1.45E-02	0.013	0.012	2.86E-01	GCKR
ILMN_1801307	TNFSF10	rs509035	G	0.68	-0.049	0.020	1.51E-02	-0.026	0.023	2.50E-01	-0.026	0.022	2.44E-01	GHSR
ILMN_2227968	NTHL1	rs1065656	G	0.72	0.039	0.016	1.55E-02	-0.001	0.018	9.63E-01	0.007	0.018	7.17E-01	NUBP2
ILMN_1678173	KHK	rs780093	T	0.39	-0.009	0.006	1.07E-01	-0.008	0.009	3.78E-01	-0.016	0.007	1.62E-02	GCKR
ILMN_1748157	EIF2B4	rs780093	T	0.39	-0.007	0.005	2.12E-01	0.011	0.005	2.32E-02	0.013	0.005	1.76E-02	GCKR
ILMN_1784352	CCM2	rs11977526	G	0.63	-0.037	0.016	1.87E-02	0.030	0.014	2.51E-02	-0.009	0.020	6.41E-01	IGFBP3
ILMN_1709613	IGF1	rs978458	T	0.27	0.065	0.028	1.88E-02	-0.004	0.012	7.60E-01	0.003	0.007	6.76E-01	IGF1
ILMN_1657237	NUBP2	rs1065656	G	0.72	0.029	0.012	1.99E-02	0.022	0.012	7.57E-02	0.035	0.016	2.88E-02	NUBP2

ILMN_1750790	GSTM5	rs646776	T	0.78	0.037	0.029	2.03E-01	0.017	0.007	2.00E-02	0.020	0.025	4.10E-01	CELSR2
ILMN_1670817	GRPEL1	rs4234798	T	0.38	0.023	0.014	1.03E-01	-0.004	0.012	7.66E-01	-0.031	0.013	2.02E-02	SORCS2
ILMN_1799100	C16orf38	rs1065656	G	0.72	-0.001	0.006	9.25E-01	0.003	0.008	7.09E-01	-0.015	0.007	2.03E-02	NUBP2
ILMN_1765438	C6orf184	rs2153960	G	0.29	-0.015	0.007	2.13E-02	-0.007	0.006	2.71E-01	0.001	0.006	8.97E-01	FOXO3
ILMN_1695005	C1orf62	rs646776	T	0.78	-0.003	0.006	5.92E-01	-0.013	0.006	2.19E-02	0.003	0.007	7.06E-01	CELSR2
ILMN_1695759	AMDHD2	rs1065656	G	0.72	0.045	0.019	2.19E-02	-0.002	0.016	8.77E-01	0.016	0.020	4.43E-01	NUBP2
ILMN_1727055	C12orf48	rs978458	T	0.27	0.011	0.007	1.16E-01	0.060	0.026	2.23E-02	-0.001	0.009	8.96E-01	IGF1
ILMN_1682428	C1orf59	rs646776	T	0.78	0.030	0.013	2.37E-02	0.025	0.025	3.33E-01	0.002	0.015	9.04E-01	CELSR2
ILMN_1765522	PPM1G	rs780093	T	0.39	-0.013	0.006	2.46E-02	-0.011	0.014	4.43E-01	0.006	0.006	3.28E-01	GCKR
ILMN_2203987	LACE1	rs2153960	G	0.29	-0.019	0.008	2.64E-02	-0.003	0.006	6.86E-01	-0.004	0.006	4.73E-01	FOXO3
ILMN_1729325	ALX3	rs646776	T	0.78	0.015	0.007	2.76E-02	-0.004	0.008	6.06E-01	0.003	0.007	6.34E-01	CELSR2
ILMN_1706690	KHK	rs780093	T	0.39	-0.002	0.005	7.46E-01	-0.003	0.009	7.58E-01	-0.011	0.005	2.78E-02	GCKR
ILMN_1698137	DNMT3A	rs934073	G	0.29	-0.009	0.006	1.21E-01	0.011	0.008	1.61E-01	-0.015	0.007	2.82E-02	ASXL2
ILMN_1717915	TRIM54	rs780093	T	0.39	0.002	0.005	7.11E-01	0.002	0.007	7.84E-01	-0.013	0.006	2.97E-02	GCKR
ILMN_1663032	FNDC4	rs780093	T	0.39	0.029	0.013	3.05E-02	0.002	0.005	7.00E-01	-0.002	0.009	7.90E-01	GCKR
ILMN_1801152	PSRC1	rs646776	T	0.78	0.016	0.007	3.14E-02	-0.015	0.007	3.22E-02	-0.015	0.008	5.15E-02	CELSR2
ILMN_1759023	WFS1	rs4234798	T	0.38	-0.041	0.019	3.14E-02	-0.004	0.020	8.33E-01	0.008	0.019	6.69E-01	SORCS2
ILMN_1810436	DNAJC27	rs934073	G	0.29	-0.001	0.012	9.49E-01	-0.012	0.011	2.79E-01	-0.030	0.014	3.24E-02	ASXL2
ILMN_2170095	PMCH	rs978458	T	0.27	-0.007	0.005	1.92E-01	0.012	0.005	3.25E-02	0.006	0.006	3.53E-01	IGF1
ILMN_1805807	SLC30A3	rs780093	T	0.39	-0.009	0.009	3.63E-01	0.016	0.008	5.97E-02	-0.025	0.012	3.29E-02	GCKR
ILMN_2061446	AADACL1	rs509035	G	0.68	-0.025	0.012	3.30E-02	0.013	0.009	1.32E-01	0.009	0.009	3.37E-01	GHSR
ILMN_1671225	GPR61	rs646776	T	0.78	0.015	0.007	3.35E-02	0.005	0.009	5.53E-01	-0.002	0.007	7.84E-01	CELSR2
ILMN_1733927	TCEB2	rs1065656	G	0.72	0.029	0.014	3.44E-02	0.018	0.014	1.75E-01	0.013	0.017	4.40E-01	NUBP2
ILMN_1801366	DNASE1L2	rs1065656	G	0.72	0.014	0.007	3.45E-02	0.008	0.007	2.35E-01	-0.023	0.037	5.44E-01	NUBP2
ILMN_1765460	AMIGO1	rs646776	T	0.78	0.014	0.007	3.62E-02	0.009	0.009	3.02E-01	0.014	0.007	4.70E-02	CELSR2
ILMN_1795704	KIAA0232	rs4234798	T	0.38	0.005	0.007	4.87E-01	-0.017	0.008	3.71E-02	0.002	0.009	7.74E-01	SORCS2
ILMN_1698968	ASXL2	rs934073	G	0.29	-0.028	0.015	7.10E-02	0.035	0.017	3.73E-02	0.025	0.021	2.36E-01	ASXL2
ILMN_2123521	SYCP3	rs978458	T	0.27	0.015	0.007	3.78E-02	-0.002	0.007	7.83E-01	-0.010	0.008	1.75E-01	IGF1
ILMN_1696003	GNAI3	rs646776	T	0.78	-0.023	0.014	1.08E-01	-0.049	0.024	3.85E-02	-0.033	0.022	1.25E-01	CELSR2
ILMN_2284013	KCNC4	rs646776	T	0.78	-0.006	0.007	3.97E-01	-0.006	0.007	3.37E-01	0.014	0.007	3.96E-02	CELSR2
ILMN_1685079	TELO2	rs1065656	G	0.72	0.030	0.015	4.20E-02	0.003	0.015	8.35E-01	0.048	0.027	7.89E-02	NUBP2
ILMN_2136068	SEC63	rs2153960	G	0.29	0.013	0.006	4.24E-02	0.008	0.008	3.10E-01	0.004	0.006	5.05E-01	FOXO3
ILMN_1701653	ASCL1	rs978458	T	0.27	0.002	0.006	7.69E-01	-0.025	0.036	4.99E-01	-0.014	0.007	4.45E-02	IGF1
ILMN_1669645	PKD1	rs1065656	G	0.72	0.019	0.016	2.26E-01	0.034	0.017	4.55E-02	0.013	0.021	5.13E-01	NUBP2
ILMN_1654408	TRAF7	rs1065656	G	0.72	0.002	0.006	6.99E-01	-0.013	0.007	5.19E-02	0.001	0.006	8.23E-01	NUBP2
ILMN_1730824	ZNF512	rs780093	T	0.39	-0.026	0.013	5.35E-02	-0.013	0.013	3.40E-01	0.007	0.014	5.99E-01	GCKR
ILMN_1701998	AFAP1	rs4234798	T	0.38	-0.031	0.016	5.39E-02	0.002	0.006	7.47E-01	0.020	0.017	2.31E-01	SORCS2
ILMN_1707713	CIB4	rs934073	G	0.29	0.005	0.005	3.38E-01	-0.005	0.005	3.40E-01	0.010	0.005	5.44E-02	ASXL2
ILMN_1725554	DNAJC5G	rs780093	T	0.39	-0.005	0.004	2.52E-01	-0.005	0.006	3.75E-01	-0.010	0.005	5.68E-02	GCKR
ILMN_1656452	C16orf59	rs1065656	G	0.72	-0.001	0.006	8.52E-01	-0.042	0.022	5.77E-02	-0.012	0.010	2.61E-01	NUBP2
ILMN_1771903	NUP37	rs978458	T	0.27	0.020	0.014	1.58E-01	0.030	0.016	5.90E-02	0.010	0.017	5.41E-01	IGF1
ILMN_1742073	ADCY1	rs700753	G	0.66	-0.009	0.005	5.93E-02	-0.003	0.006	5.76E-01	-0.002	0.005	6.97E-01	TNS3

ILMN_1674996	UNKL	rs1065656	G	0.72	-0.002	0.005	7.67E-01	-0.001	0.006	8.39E-01	0.012	0.007	6.13E-02	NUBP2
ILMN_1696830	AMPD2	rs646776	T	0.78	0.007	0.008	3.61E-01	0.018	0.010	6.29E-02	0.005	0.008	5.43E-01	CELSR2
ILMN_1671933	CLCC1	rs646776	T	0.78	-0.005	0.012	6.54E-01	-0.028	0.015	6.29E-02	-0.041	0.027	1.27E-01	CELSR2
ILMN_2328952	CLCC1	rs646776	T	0.78	0.004	0.006	5.07E-01	0.013	0.007	6.32E-02	-0.005	0.006	4.17E-01	CELSR2
ILMN_1771185	EME2	rs1065656	G	0.72	-0.011	0.006	6.42E-02	-0.006	0.007	3.76E-01	0.008	0.006	1.69E-01	NUBP2
ILMN_1741054	SLC5A6	rs780093	T	0.39	0.024	0.019	2.15E-01	0.008	0.017	6.44E-01	-0.028	0.015	6.55E-02	GCKR
ILMN_2075820	ZNF598	rs1065656	G	0.72	-0.009	0.008	2.76E-01	-0.026	0.014	7.32E-02	0.008	0.013	5.54E-01	NUBP2
ILMN_1736831	PRSS33	rs1065656	G	0.72	-0.005	0.005	3.82E-01	-0.003	0.005	5.95E-01	0.010	0.005	7.36E-02	NUBP2
ILMN_1787477	CENPO	rs934073	G	0.29	-0.001	0.005	8.62E-01	0.028	0.016	7.73E-02	-0.010	0.008	1.99E-01	ASXL2
ILMN_2403664	POMC	rs934073	G	0.29	-0.020	0.011	7.77E-02	-0.019	0.026	4.63E-01	0.001	0.010	9.24E-01	ASXL2
ILMN_1687835	C6orf224	rs2153960	G	0.29	-0.006	0.008	4.16E-01	0.010	0.006	7.78E-02	0.006	0.008	4.41E-01	FOXO3
ILMN_1739054	CCDC121	rs780093	T	0.39	-0.007	0.007	3.51E-01	-0.011	0.006	7.79E-02	0.011	0.007	8.75E-02	GCKR
ILMN_2120555	ADCY1	rs700753	G	0.66	0.017	0.010	7.93E-02	-0.005	0.017	7.63E-01	-0.010	0.008	2.07E-01	TNS3
ILMN_1732810	SNX17	rs780093	T	0.39	0.005	0.011	6.23E-01	-0.006	0.008	5.11E-01	-0.022	0.013	8.17E-02	GCKR
ILMN_1811754	NDUFB10	rs1065656	G	0.72	-0.057	0.034	9.34E-02	-0.051	0.030	8.29E-02	0.003	0.041	9.35E-01	NUBP2
ILMN_1685095	CNO	rs4234798	T	0.38	0.020	0.012	8.52E-02	0.004	0.010	6.76E-01	-0.017	0.011	1.39E-01	SORCS2
ILMN_2291152	PPP2R2C	rs4234798	T	0.38	-0.001	0.005	8.82E-01	0.010	0.006	8.52E-02	-0.004	0.006	4.37E-01	SORCS2
ILMN_1801257	CENPA	rs780093	T	0.39	0.010	0.006	9.71E-02	-0.006	0.019	7.33E-01	-0.021	0.012	8.60E-02	GCKR
ILMN_1808512	TPSD1	rs1065656	G	0.72	-0.004	0.005	4.63E-01	0.002	0.006	7.27E-01	0.010	0.006	8.66E-02	NUBP2
ILMN_2213008	ARMC2	rs2153960	G	0.29	-0.011	0.006	8.68E-02	0.008	0.007	2.58E-01	-0.008	0.006	2.07E-01	FOXO3
ILMN_2280816	CEMP1	rs1065656	G	0.72	0.004	0.008	6.69E-01	-0.003	0.013	8.13E-01	-0.016	0.010	8.68E-02	NUBP2
ILMN_1711439	EMILIN1	rs780093	T	0.39	0.022	0.013	8.70E-02	0.014	0.017	4.13E-01	-0.010	0.015	4.97E-01	GCKR
ILMN_1765833	SLC9A3R2	rs1065656	G	0.72	0.014	0.016	3.74E-01	0.000	0.006	9.53E-01	0.017	0.010	8.71E-02	NUBP2
ILMN_2169801	TPSAB1	rs1065656	G	0.72	0.070	0.041	8.75E-02	0.008	0.007	2.14E-01	0.004	0.056	9.45E-01	NUBP2
ILMN_1726417	MRPL33	rs780093	T	0.39	-0.002	0.015	8.95E-01	0.014	0.024	5.65E-01	0.027	0.016	8.77E-02	GCKR
ILMN_1751559	C16orf30	rs1065656	G	0.72	-0.007	0.016	6.58E-01	0.023	0.013	8.99E-02	0.002	0.016	8.85E-01	NUBP2
ILMN_1786789	FAM102B	rs646776	T	0.78	-0.013	0.012	2.62E-01	-0.023	0.013	9.27E-02	-0.001	0.013	9.67E-01	CELSR2
ILMN_2340886	OTOF	rs934073	G	0.29	0.010	0.006	9.44E-02	0.007	0.008	3.49E-01	-0.003	0.006	6.21E-01	ASXL2
ILMN_1758523	ABCA3	rs1065656	G	0.72	0.000	0.014	9.95E-01	-0.016	0.009	9.63E-02	-0.016	0.014	2.58E-01	NUBP2
ILMN_1805930	CSF1	rs646776	T	0.78	-0.019	0.012	9.64E-02	-0.004	0.007	5.55E-01	0.010	0.012	3.84E-01	CELSR2
ILMN_1795893	TMEM167B	rs646776	T	0.78	0.008	0.015	5.96E-01	-0.012	0.016	4.54E-01	0.029	0.017	9.69E-02	CELSR2
ILMN_1762897	GPR113	rs934073	G	0.29	0.001	0.007	8.50E-01	-0.012	0.007	9.89E-02	0.008	0.008	3.70E-01	ASXL2
ILMN_1753131	SPATA16	rs509035	G	0.68	0.001	0.005	8.97E-01	0.011	0.006	9.95E-02	-0.003	0.006	6.15E-01	GHSR
ILMN_1779256	ARMC2	rs2153960	G	0.29	0.001	0.006	8.39E-01	-0.009	0.005	1.00E-01	-0.003	0.008	7.17E-01	FOXO3
ILMN_1810554	PDPK1	rs1065656	G	0.72	-0.009	0.006	1.02E-01	0.006	0.006	3.30E-01	0.009	0.007	2.10E-01	NUBP2
ILMN_1742073	ADCY1	rs11977526	G	0.63	-0.008	0.005	1.02E-01	0.000	0.006	9.42E-01	-0.003	0.006	5.49E-01	IGFBP3
ILMN_1660551	CRAMP1L	rs1065656	G	0.72	-0.010	0.014	4.49E-01	0.018	0.012	1.48E-01	-0.028	0.017	1.03E-01	NUBP2
ILMN_1694731	CLCN7	rs1065656	G	0.72	0.029	0.018	1.04E-01	0.017	0.016	2.70E-01	0.007	0.022	7.39E-01	NUBP2
ILMN_1711720	C12orf42	rs978458	T	0.27	0.005	0.005	3.75E-01	0.010	0.006	1.04E-01	-0.005	0.006	3.75E-01	IGF1
ILMN_1789005	ATP6V0C	rs1065656	G	0.72	0.026	0.016	1.04E-01	0.004	0.009	6.67E-01	0.016	0.018	3.60E-01	NUBP2
ILMN_1672176	SMPD2	rs2153960	G	0.29	0.015	0.009	1.05E-01	0.012	0.012	3.15E-01	0.001	0.018	9.40E-01	FOXO3
ILMN_1708337	NOXO1	rs1065656	G	0.72	-0.005	0.008	5.31E-01	-0.002	0.011	8.44E-01	-0.013	0.008	1.05E-01	NUBP2

ILMN_1652198	CCM2	rs11977526	G	0.63	0.006	0.006	3.37E-01	0.010	0.014	4.99E-01	0.010	0.006	1.06E-01	IGFBP3
ILMN_1786972	SARS	rs646776	T	0.78	-0.005	0.020	8.07E-01	-0.036	0.022	1.09E-01	-0.039	0.029	1.75E-01	CELSR2
ILMN_1836744	PGP	rs1065656	G	0.72	0.016	0.010	1.09E-01	-0.006	0.011	6.05E-01	0.008	0.011	4.46E-01	NUBP2
ILMN_1759325	C16orf91	rs1065656	G	0.72	-0.017	0.011	1.11E-01	-0.004	0.013	7.93E-01	-0.013	0.015	3.87E-01	NUBP2
ILMN_1664622	KIAA0953	rs934073	G	0.29	0.006	0.005	2.90E-01	0.003	0.006	6.38E-01	0.009	0.006	1.14E-01	ASXL2
ILMN_1732127	RBKS	rs780093	T	0.39	-0.006	0.008	4.49E-01	0.001	0.009	8.96E-01	-0.014	0.009	1.16E-01	GCKR
ILMN_1720303	OSTM1	rs2153960	G	0.29	-0.023	0.019	2.08E-01	-0.015	0.010	1.18E-01	0.006	0.015	7.10E-01	FOXO3
ILMN_1810550	BAIAP3	rs1065656	G	0.72	-0.001	0.006	8.21E-01	-0.006	0.006	2.70E-01	0.009	0.006	1.21E-01	NUBP2
ILMN_1717173	ECT2	rs509035	G	0.68	-0.016	0.010	1.21E-01	-0.040	0.027	1.43E-01	-0.004	0.012	7.43E-01	GHSR
ILMN_1719696	PLD1	rs509035	G	0.68	-0.021	0.013	1.22E-01	-0.031	0.034	3.62E-01	-0.019	0.016	2.52E-01	GHSR
ILMN_1727850	KCNC4	rs646776	T	0.78	0.003	0.006	6.31E-01	-0.004	0.007	5.56E-01	-0.010	0.007	1.26E-01	CELSR2
ILMN_2197846	HADHB	rs934073	G	0.29	-0.023	0.015	1.27E-01	0.001	0.013	9.46E-01	-0.016	0.020	4.23E-01	ASXL2
ILMN_1706118	HN1L	rs1065656	G	0.72	0.017	0.011	1.29E-01	0.018	0.014	1.78E-01	0.021	0.014	1.28E-01	NUBP2
ILMN_1733930	PREB	rs780093	T	0.39	0.020	0.013	1.28E-01	-0.007	0.013	5.76E-01	-0.008	0.010	4.39E-01	GCKR
ILMN_1696133	SELI	rs934073	G	0.29	-0.006	0.009	4.89E-01	-0.004	0.013	7.46E-01	-0.028	0.019	1.29E-01	ASXL2
ILMN_1672728	KCTD5	rs1065656	G	0.72	-0.008	0.014	5.58E-01	0.026	0.017	1.30E-01	-0.010	0.019	5.83E-01	NUBP2
ILMN_1691090	MPV17	rs780093	T	0.39	-0.013	0.014	3.47E-01	0.017	0.011	1.35E-01	-0.003	0.014	8.50E-01	GCKR
ILMN_2290776	AGBL5	rs780093	T	0.39	-0.008	0.008	3.37E-01	-0.008	0.011	4.78E-01	-0.014	0.009	1.37E-01	GCKR
ILMN_1662934	UBE2I	rs1065656	G	0.72	0.010	0.007	1.38E-01	0.000	0.007	9.85E-01	0.008	0.008	3.20E-01	NUBP2
ILMN_1700889	PRR25	rs1065656	G	0.72	0.009	0.006	1.38E-01	NA	NA	NA	NA	NA	NA	NUBP2
ILMN_2218104	PAH	rs978458	T	0.27	-0.008	0.006	1.46E-01	0.009	0.006	1.41E-01	-0.004	0.006	5.41E-01	IGF1
ILMN_1663347	OTOF	rs934073	G	0.29	-0.001	0.006	9.07E-01	-0.066	0.045	1.41E-01	0.005	0.009	5.61E-01	ASXL2
ILMN_2084073	UCN	rs780093	T	0.39	0.009	0.006	1.41E-01	0.007	0.009	3.92E-01	0.000	0.007	9.76E-01	GCKR
ILMN_1731215	MYBPHL	rs646776	T	0.78	0.005	0.006	3.81E-01	0.010	0.007	1.44E-01	0.004	0.006	5.65E-01	CELSR2
ILMN_1741869	WDR47	rs646776	T	0.78	-0.005	0.017	7.74E-01	0.008	0.017	6.19E-01	0.043	0.029	1.46E-01	CELSR2
ILMN_1779886	TBC1D14	rs4234798	T	0.38	0.015	0.010	1.50E-01	-0.006	0.010	5.19E-01	-0.015	0.015	3.19E-01	SORCS2
ILMN_1758825	ABLIM2	rs4234798	T	0.38	-0.017	0.013	2.04E-01	-0.001	0.006	7.99E-01	0.024	0.017	1.52E-01	SORCS2
ILMN_1693388	NOXO1	rs1065656	G	0.72	0.006	0.006	3.00E-01	-0.009	0.006	1.52E-01	0.002	0.007	7.38E-01	NUBP2
ILMN_2397188	CACNA1H	rs1065656	G	0.72	0.008	0.010	4.15E-01	0.008	0.006	1.54E-01	-0.024	0.048	6.09E-01	NUBP2
ILMN_1801216	S100P	rs4234798	T	0.38	-0.023	0.016	1.55E-01	0.002	0.006	7.47E-01	-0.007	0.042	8.75E-01	SORCS2
ILMN_1799299	PRPF38B	rs646776	T	0.78	-0.002	0.008	7.74E-01	0.024	0.017	1.58E-01	0.007	0.011	5.42E-01	CELSR2
ILMN_1673261	EPS8L3	rs646776	T	0.78	-0.007	0.006	2.18E-01	0.004	0.006	5.34E-01	0.009	0.006	1.59E-01	CELSR2
ILMN_1778684	BRE	rs780093	T	0.39	0.011	0.008	1.60E-01	0.002	0.008	8.31E-01	-0.002	0.009	8.19E-01	GCKR
ILMN_1701589	AMPD2	rs646776	T	0.78	-0.008	0.006	1.63E-01	-0.001	0.007	8.86E-01	0.001	0.007	8.51E-01	CELSR2
ILMN_2072875	SUPT7L	rs780093	T	0.39	-0.009	0.006	1.63E-01	0.000	0.006	9.91E-01	-0.008	0.007	2.19E-01	GCKR
ILMN_1664177	ATXN7L2	rs646776	T	0.78	0.012	0.009	1.64E-01	-0.017	0.014	2.33E-01	0.009	0.015	5.63E-01	CELSR2
ILMN_2202940	CHPT1	rs978458	T	0.27	-0.022	0.016	1.65E-01	NA	NA	NA	NA	NA	NA	IGF1
ILMN_2219351	CENPO	rs934073	G	0.29	0.000	0.005	9.44E-01	0.004	0.012	7.51E-01	0.008	0.005	1.65E-01	ASXL2
ILMN_1662214	PAH	rs978458	T	0.27	0.001	0.006	8.72E-01	0.009	0.006	1.70E-01	-0.001	0.006	8.74E-01	IGF1
ILMN_2060212	TBC1D24	rs1065656	G	0.72	-0.024	0.017	1.74E-01	0.017	0.014	2.15E-01	-0.001	0.012	9.37E-01	NUBP2
ILMN_1746085	IGFBP3	rs700753	G	0.66	0.014	0.022	5.29E-01	0.008	0.006	1.75E-01	-0.012	0.028	6.74E-01	TNS3
ILMN_1676336	AADACL1	rs509035	G	0.68	-0.008	0.023	7.16E-01	0.018	0.013	1.79E-01	0.003	0.013	8.13E-01	GHSR

ILMN_1709604	SNORA64	rs1065656	G	0.72	0.003	0.007	6.32E-01	-0.023	0.017	1.81E-01	-0.005	0.013	7.08E-01	NUBP2
ILMN_1669456	NME3	rs1065656	G	0.72	0.014	0.011	1.83E-01	0.019	0.028	4.85E-01	-0.024	0.019	2.16E-01	NUBP2
ILMN_1786899	AMPD2	rs646776	T	0.78	0.009	0.007	1.85E-01	-0.007	0.008	4.13E-01	0.008	0.007	2.43E-01	CELSR2
ILMN_1792382	KCNC4	rs646776	T	0.78	-0.008	0.006	2.00E-01	-0.009	0.007	1.86E-01	-0.003	0.006	6.35E-01	CELSR2
ILMN_1695740	C1QTNF8	rs1065656	G	0.72	0.007	0.006	2.33E-01	-0.002	0.008	7.68E-01	-0.009	0.007	1.86E-01	NUBP2
ILMN_1770412	AHCYL1	rs646776	T	0.78	0.008	0.016	6.05E-01	-0.020	0.015	1.89E-01	-0.004	0.022	8.56E-01	CELSR2
ILMN_1799710	CCDC96	rs4234798	T	0.38	-0.001	0.005	9.15E-01	-0.008	0.006	1.92E-01	0.000	0.006	9.84E-01	SORCS2
ILMN_1712515	FOXO3	rs2153960	G	0.29	0.005	0.006	4.35E-01	0.005	0.008	5.48E-01	0.009	0.007	1.92E-01	FOXO3
ILMN_1797277	KIF3C	rs934073	G	0.29	0.007	0.006	2.60E-01	-0.006	0.006	3.35E-01	-0.010	0.008	1.93E-01	ASXL2
ILMN_1717492	GHSR	rs509035	G	0.68	-0.003	0.006	5.46E-01	0.009	0.007	2.12E-01	0.008	0.006	1.93E-01	GHSR
ILMN_1669376	DRAM1	rs978458	T	0.27	0.008	0.013	5.38E-01	0.022	0.017	1.95E-01	0.004	0.013	7.79E-01	IGF1
ILMN_1894057	NA	rs1065656	G	0.72	0.005	0.008	5.30E-01	-0.004	0.007	5.20E-01	0.012	0.009	1.95E-01	NUBP2
ILMN_2139816	GPSM2	rs646776	T	0.78	-0.006	0.009	5.12E-01	0.003	0.015	8.35E-01	-0.036	0.028	1.97E-01	CELSR2
ILMN_2126408	UNKL	rs1065656	G	0.72	0.009	0.007	1.98E-01	0.005	0.008	4.84E-01	0.009	0.008	2.55E-01	NUBP2
ILMN_1687940	FOXO3	rs2153960	G	0.29	-0.007	0.006	2.01E-01	-0.004	0.006	4.68E-01	-0.001	0.005	8.13E-01	FOXO3
ILMN_1811574	MAPK8IP3	rs1065656	G	0.72	0.017	0.016	2.99E-01	0.030	0.034	3.83E-01	0.028	0.022	2.02E-01	NUBP2
ILMN_1676893	ADCY3	rs934073	G	0.29	0.008	0.016	6.21E-01	-0.013	0.015	3.70E-01	-0.019	0.015	2.04E-01	ASXL2
ILMN_1728445	IGFBP1	rs11977526	G	0.63	-0.006	0.005	2.05E-01	0.001	0.006	8.99E-01	-0.003	0.006	5.91E-01	IGFBP3
ILMN_1769219	TPSG1	rs1065656	G	0.72	-0.008	0.007	2.06E-01	-0.004	0.007	5.86E-01	-0.005	0.011	6.55E-01	NUBP2
ILMN_1806691	GHSR	rs509035	G	0.68	0.001	0.006	8.13E-01	0.000	0.007	9.70E-01	-0.008	0.006	2.06E-01	GHSR
ILMN_1657058	PPIL6	rs2153960	G	0.29	-0.003	0.006	6.00E-01	0.001	0.006	8.33E-01	0.008	0.006	2.08E-01	FOXO3
ILMN_1691843	RNPS1	rs1065656	G	0.72	-0.014	0.011	2.14E-01	-0.004	0.012	7.13E-01	-0.004	0.013	7.83E-01	NUBP2
ILMN_1714987	TRIM54	rs780093	T	0.39	-0.006	0.005	2.17E-01	-0.003	0.005	5.61E-01	-0.001	0.005	7.86E-01	GCKR
ILMN_1786379	SYNGR3	rs1065656	G	0.72	0.001	0.006	8.54E-01	0.025	0.021	2.19E-01	0.000	0.006	1.00E+00	NUBP2
ILMN_1660424	AFAP1	rs4234798	T	0.38	-0.006	0.005	2.93E-01	-0.006	0.005	2.19E-01	-0.002	0.005	7.35E-01	SORCS2
ILMN_1736184	GSTM3	rs646776	T	0.78	0.045	0.037	2.20E-01	0.001	0.009	9.13E-01	-0.005	0.034	8.75E-01	CELSR2
ILMN_1788729	TCF23	rs780093	T	0.39	0.000	0.004	9.77E-01	0.007	0.006	2.22E-01	0.002	0.005	7.36E-01	GCKR
ILMN_1812615	MYBPC1	rs978458	T	0.27	0.006	0.006	3.12E-01	0.008	0.007	2.25E-01	0.001	0.010	9.27E-01	IGF1
ILMN_1815530	GCKR	rs780093	T	0.39	-0.006	0.005	2.33E-01	0.001	0.005	8.79E-01	-0.005	0.006	4.12E-01	GCKR
ILMN_1762631	TSC2	rs1065656	G	0.72	0.003	0.006	5.65E-01	0.006	0.005	2.34E-01	0.006	0.005	2.65E-01	NUBP2
ILMN_1690563	SSTR5	rs1065656	G	0.72	0.007	0.006	2.40E-01	0.001	0.006	8.38E-01	0.001	0.006	8.82E-01	NUBP2
ILMN_1723837	SLC6A17	rs646776	T	0.78	0.002	0.006	6.83E-01	0.008	0.007	2.45E-01	-0.008	0.022	7.01E-01	CELSR2
ILMN_1713162	GSTM2	rs646776	T	0.78	0.029	0.025	2.48E-01	0.017	0.035	6.36E-01	-0.012	0.027	6.53E-01	CELSR2
ILMN_1773119	CCNF	rs1065656	G	0.72	0.002	0.017	8.97E-01	-0.021	0.018	2.48E-01	0.016	0.025	5.18E-01	NUBP2
ILMN_1768646	FLJ32252	rs1065656	G	0.72	-0.006	0.005	2.48E-01	-0.003	0.006	6.09E-01	0.005	0.006	3.50E-01	NUBP2
ILMN_1773849	ATP6V0C	rs1065656	G	0.72	-0.011	0.018	5.53E-01	0.011	0.013	4.24E-01	-0.026	0.023	2.51E-01	NUBP2
ILMN_1687442	TNIK	rs509035	G	0.68	0.000	0.006	9.43E-01	-0.015	0.024	5.35E-01	-0.006	0.005	2.55E-01	GHSR
ILMN_1812688	C2orf18	rs780093	T	0.39	0.005	0.010	6.58E-01	-0.004	0.009	6.48E-01	-0.018	0.016	2.56E-01	GCKR
ILMN_1806394	UBL4B	rs646776	T	0.78	0.005	0.006	4.42E-01	0.007	0.006	2.68E-01	0.007	0.006	2.56E-01	CELSR2
ILMN_1654429	C12orf48	rs978458	T	0.27	0.000	0.006	1.00E+00	-0.002	0.008	7.59E-01	0.008	0.007	2.57E-01	IGF1
ILMN_1764827	MRFAP1L1	rs4234798	T	0.38	0.000	0.005	9.97E-01	-0.009	0.008	2.59E-01	0.000	0.007	9.71E-01	SORCS2
ILMN_2302947	CSF1	rs646776	T	0.78	-0.007	0.006	2.61E-01	0.002	0.006	7.77E-01	0.005	0.007	4.83E-01	CELSR2

ILMN_2356574	GTF3C2	rs780093	T	0.39	-0.002	0.008	7.43E-01	0.006	0.010	5.31E-01	0.012	0.011	2.61E-01	GCKR
ILMN_1768510	MAN2B2	rs4234798	T	0.38	-0.008	0.019	6.89E-01	-0.016	0.014	2.61E-01	-0.018	0.024	4.51E-01	SORCS2
ILMN_1692271	CCM2	rs11977526	G	0.63	0.006	0.006	3.43E-01	0.000	0.015	9.85E-01	0.009	0.008	2.62E-01	IGFBP3
ILMN_1738580	NR2E1	rs2153960	G	0.29	0.003	0.008	7.30E-01	-0.008	0.007	2.65E-01	-0.006	0.009	4.58E-01	FOXO3
ILMN_1745330	RNF151	rs1065656	G	0.72	0.006	0.006	2.93E-01	0.007	0.006	2.67E-01	-0.004	0.006	5.56E-01	NUBP2
ILMN_1734766	C6orf182	rs2153960	G	0.29	0.003	0.005	5.71E-01	-0.007	0.006	2.72E-01	-0.002	0.006	8.01E-01	FOXO3
ILMN_1679039	CASKIN1	rs1065656	G	0.72	0.006	0.006	3.61E-01	-0.006	0.005	2.74E-01	-0.005	0.006	3.37E-01	NUBP2
ILMN_1743371	ABHD1	rs780093	T	0.39	-0.012	0.011	2.75E-01	-0.004	0.006	5.06E-01	-0.005	0.006	3.68E-01	GCKR
ILMN_1754158	C6orf185	rs2153960	G	0.29	-0.005	0.018	7.77E-01	-0.005	0.005	2.79E-01	0.001	0.006	8.76E-01	FOXO3
ILMN_1746085	IGFBP3	rs11977526	G	0.63	-0.004	0.022	8.64E-01	0.006	0.006	2.80E-01	0.006	0.029	8.47E-01	IGFBP3
ILMN_1658805	BRE	rs780093	T	0.39	0.004	0.005	3.90E-01	-0.006	0.006	2.81E-01	-0.003	0.006	6.31E-01	GCKR
ILMN_1664542	UBE2I	rs1065656	G	0.72	-0.006	0.006	2.87E-01	-0.001	0.009	9.30E-01	-0.006	0.008	4.41E-01	NUBP2
ILMN_2320377	MAPK8IP3	rs1065656	G	0.72	-0.005	0.006	3.90E-01	0.008	0.008	2.91E-01	-0.007	0.007	3.07E-01	NUBP2
ILMN_1790317	RAB26	rs1065656	G	0.72	-0.009	0.008	3.06E-01	-0.003	0.009	7.24E-01	0.017	0.023	4.58E-01	NUBP2
ILMN_1771482	KIAA1324	rs646776	T	0.78	0.001	0.009	9.41E-01	0.019	0.018	3.06E-01	0.000	0.045	9.91E-01	CELSR2
ILMN_2315964	PSRC1	rs646776	T	0.78	0.008	0.008	3.08E-01	-0.009	0.010	3.34E-01	0.002	0.008	7.82E-01	CELSR2
ILMN_2396875	IGFBP3	rs700753	G	0.66	0.019	0.019	3.26E-01	0.006	0.006	3.08E-01	0.000	0.024	9.97E-01	TNS3
ILMN_1740180	SNX3	rs2153960	G	0.29	-0.002	0.018	9.26E-01	-0.010	0.016	5.26E-01	0.025	0.025	3.09E-01	FOXO3
ILMN_1804509	DPYSL5	rs780093	T	0.39	0.005	0.007	4.20E-01	0.007	0.007	3.09E-01	-0.001	0.006	9.28E-01	GCKR
ILMN_1676128	DNMT3A	rs934073	G	0.29	0.005	0.006	4.38E-01	-0.015	0.015	3.12E-01	-0.001	0.007	8.45E-01	ASXL2
ILMN_1671263	CACNA1H	rs1065656	G	0.72	0.014	0.014	3.12E-01	0.006	0.008	4.39E-01	-0.021	0.057	7.06E-01	NUBP2
ILMN_1756595	SH3TC1	rs4234798	T	0.38	-0.014	0.014	3.14E-01	-0.011	0.018	5.55E-01	0.005	0.013	7.07E-01	SORCS2
ILMN_2335198	NCOA1	rs934073	G	0.29	0.004	0.006	4.67E-01	0.003	0.006	6.69E-01	0.006	0.006	3.15E-01	ASXL2
ILMN_1725175	FOSL2	rs780093	T	0.39	0.002	0.005	7.10E-01	-0.002	0.006	7.85E-01	-0.006	0.006	3.18E-01	GCKR
ILMN_1728445	IGFBP1	rs700753	G	0.66	-0.002	0.005	6.57E-01	0.002	0.005	6.66E-01	0.005	0.005	3.19E-01	TNS3
ILMN_1707689	FAM59B	rs934073	G	0.29	0.006	0.008	4.64E-01	-0.008	0.008	3.24E-01	-0.005	0.007	4.56E-01	ASXL2
ILMN_1809267	CLCC1	rs646776	T	0.78	0.002	0.011	8.23E-01	0.011	0.015	4.50E-01	0.013	0.013	3.24E-01	CELSR2
ILMN_1661129	PPP2R2C	rs4234798	T	0.38	0.005	0.005	3.26E-01	0.002	0.005	6.50E-01	0.000	0.006	9.95E-01	SORCS2
ILMN_1707713	CIB4	rs780093	T	0.39	0.000	0.004	9.32E-01	-0.004	0.005	3.27E-01	-0.003	0.005	5.81E-01	GCKR
ILMN_1667893	TNS3	rs700753	G	0.66	0.009	0.020	6.66E-01	0.019	0.025	4.55E-01	-0.021	0.021	3.28E-01	TNS3
ILMN_1734602	SRRM2	rs1065656	G	0.72	0.009	0.014	4.98E-01	0.015	0.015	3.29E-01	0.005	0.022	8.19E-01	NUBP2
ILMN_1783728	TBRG4	rs11977526	G	0.63	-0.011	0.012	3.30E-01	-0.009	0.013	4.80E-01	-0.005	0.014	7.24E-01	IGFBP3
ILMN_1707077	SORT1	rs646776	T	0.78	-0.007	0.016	6.63E-01	0.012	0.015	4.37E-01	-0.018	0.019	3.35E-01	CELSR2
ILMN_1783852	CD164	rs2153960	G	0.29	0.008	0.011	4.57E-01	-0.014	0.014	3.43E-01	-0.005	0.007	4.69E-01	FOXO3
ILMN_2330170	MYBPC1	rs978458	T	0.27	0.002	0.006	6.60E-01	0.006	0.006	3.44E-01	0.004	0.015	7.84E-01	IGF1
ILMN_1789240	MLST8	rs1065656	G	0.72	0.002	0.011	8.77E-01	-0.005	0.012	6.61E-01	-0.014	0.014	3.47E-01	NUBP2
ILMN_1720274	C2orf53	rs780093	T	0.39	0.004	0.005	3.48E-01	-0.002	0.005	7.44E-01	-0.001	0.005	8.45E-01	GCKR
ILMN_1742179	FNDC7	rs646776	T	0.78	0.002	0.006	7.76E-01	-0.006	0.006	3.49E-01	-0.002	0.006	7.21E-01	CELSR2
ILMN_1651235	AFAP1	rs4234798	T	0.38	0.001	0.005	8.97E-01	0.005	0.005	3.49E-01	-0.004	0.005	4.14E-01	SORCS2
ILMN_1692295	MYO1G	rs11977526	G	0.63	-0.006	0.013	6.70E-01	-0.005	0.017	7.64E-01	-0.016	0.017	3.55E-01	IGFBP3
ILMN_2203073	RNF151	rs1065656	G	0.72	0.000	0.006	9.72E-01	0.004	0.007	5.96E-01	-0.005	0.006	3.55E-01	NUBP2
ILMN_1689774	MRFAP1L1	rs4234798	T	0.38	-0.009	0.010	3.57E-01	-0.001	0.007	9.25E-01	0.010	0.011	3.69E-01	SORCS2

ILMN_1764230	GNPTG	rs1065656	G	0.72	-0.001	0.017	9.31E-01	0.016	0.018	3.60E-01	-0.008	0.018	6.54E-01	NUBP2
ILMN_2396875	IGFBP3	rs11977526	G	0.63	-0.018	0.020	3.64E-01	0.003	0.006	6.36E-01	0.022	0.025	3.80E-01	IGFBP3
ILMN_1768483	KCNK3	rs934073	G	0.29	0.002	0.006	8.02E-01	0.002	0.007	7.89E-01	0.006	0.007	3.69E-01	ASXL2
ILMN_1795557	SNX3	rs2153960	G	0.29	-0.001	0.005	8.80E-01	-0.005	0.006	3.74E-01	0.004	0.006	5.02E-01	FOXO3
ILMN_1653056	ATP6V0C	rs1065656	G	0.72	0.003	0.005	5.42E-01	0.000	0.007	9.40E-01	-0.006	0.007	3.74E-01	NUBP2
ILMN_2205785	AKNAD1	rs646776	T	0.78	0.002	0.006	7.66E-01	0.001	0.006	8.18E-01	-0.006	0.006	3.82E-01	CELSR2
ILMN_1810474	UBE2I	rs1065656	G	0.72	-0.006	0.010	5.36E-01	0.009	0.010	3.82E-01	-0.009	0.014	5.06E-01	NUBP2
ILMN_1693702	MRFAP1L1	rs4234798	T	0.38	0.006	0.007	4.59E-01	0.007	0.009	4.59E-01	-0.008	0.009	3.84E-01	SORCS2
ILMN_2414848	TBRG4	rs11977526	G	0.63	0.003	0.010	7.97E-01	0.009	0.010	3.97E-01	0.002	0.015	9.15E-01	IGFBP3
ILMN_1716979	GSTM4	rs646776	T	0.78	0.010	0.012	4.00E-01	-0.004	0.022	8.44E-01	0.000	0.009	9.86E-01	CELSR2
ILMN_1809047	TBRG4	rs11977526	G	0.63	0.004	0.006	4.45E-01	0.000	0.015	9.76E-01	0.006	0.007	4.00E-01	IGFBP3
ILMN_1772719	GPN1	rs780093	T	0.39	-0.009	0.011	4.07E-01	-0.005	0.008	5.60E-01	-0.009	0.011	4.03E-01	GCKR
ILMN_1654945	DNMT3A	rs934073	G	0.29	0.001	0.006	8.33E-01	0.000	0.011	9.66E-01	-0.006	0.007	4.09E-01	ASXL2
ILMN_1752075	MYBPC1	rs978458	T	0.27	-0.005	0.006	4.15E-01	0.003	0.007	6.88E-01	-0.003	0.022	8.92E-01	IGF1
ILMN_2065745	RAMP3	rs11977526	G	0.63	-0.020	0.025	4.20E-01	0.000	0.005	9.69E-01	0.003	0.020	8.84E-01	IGFBP3
ILMN_1736757	GNPTAB	rs978458	T	0.27	-0.002	0.013	8.78E-01	-0.006	0.018	7.20E-01	0.015	0.019	4.24E-01	IGF1
ILMN_2392472	CENPA	rs780093	T	0.39	-0.001	0.006	8.67E-01	0.011	0.018	5.29E-01	-0.006	0.008	4.30E-01	GCKR
ILMN_1791069	ZNF513	rs780093	T	0.39	0.003	0.006	6.65E-01	-0.002	0.008	7.88E-01	-0.008	0.011	4.34E-01	GCKR
ILMN_1747084	FLJ42177	rs2153960	G	0.29	0.000	0.005	9.77E-01	0.004	0.005	4.37E-01	0.004	0.005	4.42E-01	FOXO3
ILMN_1774722	CGREF1	rs780093	T	0.39	0.004	0.005	4.41E-01	0.002	0.006	6.86E-01	-0.004	0.005	4.59E-01	GCKR
ILMN_1768483	KCNK3	rs780093	T	0.39	-0.004	0.006	4.42E-01	0.004	0.006	5.03E-01	0.000	0.006	9.97E-01	GCKR
ILMN_2412046	TRIM54	rs780093	T	0.39	0.004	0.005	4.59E-01	0.002	0.005	6.74E-01	-0.004	0.006	4.90E-01	GCKR
ILMN_2191902	ALX3	rs646776	T	0.78	-0.001	0.006	8.29E-01	-0.005	0.006	4.65E-01	0.004	0.006	5.47E-01	CELSR2
ILMN_2056087	IGF1	rs978458	T	0.27	0.006	0.017	7.31E-01	0.004	0.008	5.75E-01	-0.004	0.006	4.78E-01	IGF1
ILMN_1708957	MYBPC1	rs978458	T	0.27	0.002	0.007	7.47E-01	-0.002	0.009	8.31E-01	-0.005	0.007	4.94E-01	IGF1
ILMN_1680507	PPP2R2C	rs4234798	T	0.38	-0.004	0.005	4.99E-01	-0.002	0.005	7.36E-01	-0.016	0.023	4.95E-01	SORCS2
ILMN_1773758	PDPK1	rs1065656	G	0.72	-0.004	0.007	5.01E-01	-0.001	0.007	9.29E-01	-0.004	0.007	5.34E-01	NUBP2
ILMN_1692707	C2orf79	rs934073	G	0.29	-0.007	0.010	5.02E-01	0.002	0.014	8.61E-01	-0.003	0.014	8.41E-01	ASXL2
ILMN_1755111	C6orf199	rs2153960	G	0.29	-0.003	0.006	6.53E-01	0.002	0.008	7.45E-01	0.004	0.007	5.13E-01	FOXO3
ILMN_1725529	UBE2I	rs1065656	G	0.72	0.000	0.006	9.57E-01	-0.004	0.007	6.10E-01	0.004	0.006	5.16E-01	NUBP2
ILMN_1712751	HADHA	rs934073	G	0.29	0.014	0.021	5.17E-01	-0.005	0.018	7.86E-01	0.005	0.021	8.15E-01	ASXL2
ILMN_2401344	PPP2R2C	rs4234798	T	0.38	0.003	0.006	5.82E-01	-0.004	0.007	5.48E-01	-0.004	0.024	8.61E-01	SORCS2
ILMN_1679279	SYPL2	rs646776	T	0.78	-0.002	0.008	8.18E-01	0.004	0.008	5.54E-01	0.000	0.009	9.82E-01	CELSR2
ILMN_2363489	BRE	rs780093	T	0.39	-0.006	0.010	5.55E-01	0.006	0.011	6.07E-01	0.003	0.014	8.10E-01	GCKR
ILMN_2094294	NR2E1	rs2153960	G	0.29	0.002	0.005	7.72E-01	0.003	0.006	5.58E-01	0.001	0.006	8.16E-01	FOXO3
ILMN_1672899	POMC	rs934073	G	0.29	-0.001	0.006	8.25E-01	-0.003	0.011	7.60E-01	0.003	0.007	6.07E-01	ASXL2
ILMN_1692826	UNKL	rs1065656	G	0.72	0.008	0.017	6.37E-01	-0.009	0.017	6.10E-01	0.006	0.016	7.24E-01	NUBP2
ILMN_1734290	MAPRE3	rs780093	T	0.39	0.003	0.013	8.35E-01	0.005	0.010	6.34E-01	0.003	0.014	8.18E-01	GCKR
ILMN_1807972	MICAL1	rs2153960	G	0.29	-0.002	0.021	9.11E-01	0.010	0.021	6.39E-01	-0.008	0.022	7.27E-01	FOXO3
ILMN_1720287	E4F1	rs1065656	G	0.72	0.005	0.015	7.59E-01	0.001	0.013	9.41E-01	0.009	0.020	6.41E-01	NUBP2
ILMN_1736929	PLB1	rs780093	T	0.39	-0.002	0.005	6.75E-01	-0.003	0.006	6.55E-01	-0.002	0.007	7.91E-01	GCKR
ILMN_1785785	NCOA1	rs934073	G	0.29	0.003	0.007	7.02E-01	0.006	0.013	6.60E-01	0.000	0.010	9.68E-01	ASXL2

ILMN_2191940	SPIC	rs978458	T	0.27	0.001	0.005	7.94E-01	-0.002	0.006	6.75E-01	-0.002	0.006	8.06E-01	IGF1
ILMN_1768055	GFER	rs1065656	G	0.72	0.004	0.011	7.02E-01	NA	NA	NA	NA	NA	NA	NUBP2
ILMN_1780840	C2orf16	rs780093	T	0.39	0.001	0.005	8.78E-01	0.000	0.006	9.39E-01	-0.002	0.005	7.03E-01	GCKR
ILMN_1719661	SEPX1	rs1065656	G	0.72	-0.006	0.016	7.07E-01	0.000	0.018	9.82E-01	-0.004	0.028	8.81E-01	NUBP2
ILMN_2120555	ADCY1	rs11977526	G	0.63	0.001	0.010	9.29E-01	-0.006	0.017	7.15E-01	-0.002	0.008	8.22E-01	IGFBP3
ILMN_1780197	TBC1D24	rs1065656	G	0.72	-0.001	0.006	7.93E-01	0.001	0.006	8.26E-01	0.002	0.006	7.29E-01	NUBP2
ILMN_1717888	KHK	rs780093	T	0.39	-0.001	0.005	7.88E-01	0.001	0.006	8.67E-01	-0.001	0.006	8.16E-01	GCKR

Results are ordered by minimum p-value across all tissues.
Significant results after Bonferroni correction are marked in bold.

Supplementary Table 7: Results of gene expressison analysis of the genome-wide significant loci with IGF-I and IGFBP-3 in the SHIP-TREND cohort

Trait	Probe Name	Gene Name	beta	SE	p-value	FDR	log2 Mean Intensity	GWAS Locus
IGFBP3	5870328	RPS2	-3.88E-05	8.04E-06	1.64E-06	**5.30E-04**	13.97	NUBP2
IGFBP3	4900333	HAGH	8.68E-05	2.15E-05	6.00E-05	**9.69E-03**	10.69	NUBP2
IGFBP3	770554	RPS2	-3.76E-05	1.01E-05	2.04E-04	**2.19E-02**	12.33	NUBP2
IGFBP3	1780356	HAGH	8.70E-05	2.42E-05	3.45E-04	**2.79E-02**	8.89	NUBP2
IGFBP3	2370341	RPS2	-3.23E-05	9.15E-06	4.31E-04	**2.79E-02**	13.70	NUBP2
IGF1	1440243	SEPX1	8.38E-04	2.48E-04	7.50E-04	**4.04E-02**	12.70	NUBP2
IGF1	3610722	GPSM2	5.75E-04	1.76E-04	1.10E-03	5.08E-02	6.97	CELSR2
IGFBP3	6200243	PGP	-2.42E-05	7.53E-06	1.33E-03	5.39E-02	6.59	NUBP2
IGF1	5870328	RPS2	-5.43E-04	1.73E-04	1.72E-03	6.18E-02	13.97	NUBP2
IGF1	830605	CCDC121	-3.27E-04	1.06E-04	2.04E-03	6.60E-02	6.23	GCKR
IGFBP3	2070184	NTHL1	-2.15E-05	7.07E-06	2.44E-03	7.15E-02	6.34	NUBP2
IGF1	2630022	RNPS1	-5.28E-04	1.81E-04	3.52E-03	9.46E-02	9.00	NUBP2
IGFBP3	6450424	NME3	-2.32E-05	8.28E-06	5.24E-03	1.21E-01	8.69	NUBP2
IGF1	6900592	GSTM4	4.06E-04	1.44E-04	5.08E-03	1.21E-01	6.21	CELSR2
IGF1	770554	RPS2	-5.71E-04	2.16E-04	8.35E-03	1.59E-01	12.33	NUBP2
IGFBP3	3710215	MLST8	-2.13E-05	8.01E-06	8.10E-03	1.59E-01	6.84	NUBP2
IGFBP3	4890056	C16ORF91	-2.13E-05	8.00E-06	7.78E-03	1.59E-01	7.26	NUBP2
IGF1	3140008	PPM1G	-4.42E-04	1.71E-04	1.00E-02	1.70E-01	9.69	GCKR
IGFBP3	3370497	TSC2	1.18E-05	4.54E-06	9.52E-03	1.70E-01	6.15	NUBP2
IGF1	3890544	NUP37	-4.42E-04	1.75E-04	1.17E-02	1.90E-01	8.18	IGF1
IGF1	7570671	UNKL	4.60E-04	1.84E-04	1.28E-02	1.96E-01	6.85	NUBP2
IGF1	6560338	ZNF512	-4.72E-04	1.96E-04	1.62E-02	2.29E-01	9.26	GCKR
IGF1	4150553	TAF13	2.56E-04	1.06E-04	1.63E-02	2.29E-01	6.19	CELSR2
IGF1	6450424	NME3	-4.16E-04	1.77E-04	1.88E-02	2.53E-01	8.69	NUBP2
IGF1	5570594	STXBP3	5.41E-04	2.32E-04	2.00E-02	2.58E-01	7.71	CELSR2
IGFBP3	1240612	MAPK8IP3	-1.20E-05	5.23E-06	2.14E-02	2.66E-01	6.02	NUBP2
IGF1	6370494	EIF2B4	-3.37E-04	1.50E-04	2.48E-02	2.97E-01	8.62	GCKR
IGF1	4220576	SLC4A1AP	3.33E-04	1.50E-04	2.69E-02	3.11E-01	6.40	GCKR
IGFBP3	5080451	PSMA5	-1.99E-05	9.03E-06	2.80E-02	3.12E-01	9.39	CELSR2
IGFBP3	3940138	PKD1	-1.88E-05	8.67E-06	3.07E-02	3.31E-01	6.93	NUBP2
IGFBP3	6060592	GNAI3	2.67E-05	1.28E-05	3.69E-02	3.84E-01	7.95	CELSR2
IGF1	870484	LACE1	-2.19E-04	1.07E-04	4.04E-02	3.87E-01	6.12	FOXO3
IGF1	6940132	AMPD2	2.28E-04	1.12E-04	4.17E-02	3.87E-01	6.09	CELSR2
IGFBP3	160019	SORT1	-2.33E-05	1.13E-05	3.94E-02	3.87E-01	7.69	CELSR2
IGFBP3	3130241	TMEM167B	-1.89E-05	9.29E-06	4.19E-02	3.87E-01	8.32	CELSR2
IGF1	6840215	ECT2	-3.51E-04	1.74E-04	4.43E-02	3.97E-01	6.72	GHSR
IGFBP3	5270575	MAPK8IP3	-1.69E-05	8.52E-06	4.75E-02	4.04E-01	8.62	NUBP2

IGF1	5080451	PSMA5	-3.84E-04	1.93E-04	4.66E-02	4.04E-01	9.39	CELSR2
IGFBP3	5340066	CRAMP1L	-1.57E-05	8.03E-06	5.16E-02	4.28E-01	6.98	NUBP2
IGF1	6960059	GCKR	-2.18E-04	1.13E-04	5.32E-02	4.30E-01	6.34	GCKR
IGF1	6060592	GNAI3	5.16E-04	2.72E-04	5.84E-02	4.60E-01	7.95	CELSR2
IGFBP3	2100079	ECI1	-1.55E-05	8.25E-06	6.11E-02	4.70E-01	6.55	NUBP2
IGFBP3	7000035	HS3ST6	1.13E-05	6.06E-06	6.33E-02	4.73E-01	6.22	NUBP2
IGF1	7550138	SYPL2	2.10E-04	1.13E-04	6.44E-02	4.73E-01	6.06	CELSR2
IGF1	3130286	no GeneName	-1.91E-04	1.04E-04	6.66E-02	4.78E-01	6.15	CELSR2
IGF1	990136	C12ORF48	-2.23E-04	1.22E-04	6.90E-02	4.85E-01	6.21	IGF1
IGF1	1710332	FAHD1	-1.93E-04	1.07E-04	7.20E-02	4.85E-01	6.17	NUBP2
IGF1	6650168	NR2E1	-1.91E-04	1.06E-04	7.18E-02	4.85E-01	6.20	FOXO3
IGF1	5360026	no GeneName	-1.95E-04	1.09E-04	7.37E-02	4.86E-01	6.30	ASXL2
IGF1	3800025	C2ORF28	-3.14E-04	1.78E-04	7.85E-02	5.07E-01	8.64	GCKR
IGF1	20338	NOXO1	2.91E-04	1.73E-04	9.28E-02	5.11E-01	6.24	NUBP2
IGF1	2230626	MRPL33	-3.65E-04	2.17E-04	9.33E-02	5.11E-01	8.13	GCKR
IGF1	430402	ABCA3	1.74E-04	1.04E-04	9.25E-02	5.11E-01	6.13	NUBP2
IGF1	5570494	MRPL33	-3.23E-04	1.89E-04	8.87E-02	5.11E-01	10.00	GCKR
IGF1	6770341	PMCH	-1.82E-04	1.06E-04	8.58E-02	5.11E-01	6.16	IGF1
IGFBP3	4610201	SNORA10	2.08E-05	1.22E-05	8.77E-02	5.11E-01	7.25	NUBP2
IGFBP3	580270	NOXO1	8.75E-06	5.07E-06	8.46E-02	5.11E-01	6.14	NUBP2
IGF1	1070762	PSRC1	2.71E-04	1.59E-04	8.75E-02	5.11E-01	6.58	CELSR2
IGFBP3	3710240	CELSR2	-8.11E-06	4.69E-06	8.45E-02	5.11E-01	6.04	CELSR2
IGF1	6900367	BRE	-1.70E-04	1.02E-04	9.66E-02	5.16E-01	6.23	GCKR
IGFBP3	2760601	C16ORF73	-8.11E-06	4.91E-06	9.85E-02	5.16E-01	6.19	NUBP2
IGFBP3	520609	KIAA1324	-3.76E-05	2.28E-05	9.90E-02	5.16E-01	7.63	CELSR2
IGF1	3520711	MPV17	-2.92E-04	1.78E-04	1.01E-01	5.18E-01	7.43	GCKR
IGF1	2030070	ZNF598	-2.92E-04	1.81E-04	1.08E-01	5.21E-01	8.17	NUBP2
IGF1	2370341	RPS2	-3.17E-04	1.96E-04	1.06E-01	5.21E-01	13.70	NUBP2
IGFBP3	450161	FAHD1	1.75E-05	1.08E-05	1.07E-01	5.21E-01	6.95	NUBP2
IGFBP3	4060707	GSTM4;GSTM2	-8.72E-06	5.38E-06	1.05E-01	5.21E-01	6.23	CELSR2
IGF1	60220	C16ORF79	2.27E-04	1.43E-04	1.13E-01	5.30E-01	6.58	NUBP2
IGFBP3	3610722	GPSM2	1.31E-05	8.27E-06	1.13E-01	5.30E-01	6.97	CELSR2
IGF1	3360468	SNX17	-2.40E-04	1.53E-04	1.17E-01	5.30E-01	9.66	GCKR
IGFBP3	610592	ADCY1	8.11E-06	5.16E-06	1.16E-01	5.30E-01	6.29	IGFBP3
IGF1	6040026	GHSR	-2.06E-04	1.33E-04	1.20E-01	5.38E-01	6.26	GHSR
IGF1	2140064	GTF3C2	-2.69E-04	1.77E-04	1.30E-01	5.45E-01	8.71	GCKR
IGF1	3710215	MLST8	-2.63E-04	1.71E-04	1.25E-01	5.45E-01	6.84	NUBP2
IGF1	5570047	GHSR	-1.93E-04	1.27E-04	1.30E-01	5.45E-01	6.15	GHSR
IGFBP3	4230301	PGP	1.04E-05	6.81E-06	1.28E-01	5.45E-01	6.66	NUBP2
IGFBP3	5910528	DNASE1L2	8.12E-06	5.27E-06	1.24E-01	5.45E-01	6.28	NUBP2
IGFBP3	7150598	GSTM3	1.61E-05	1.07E-05	1.32E-01	5.48E-01	6.53	CELSR2
IGF1	3370497	TSC2	1.39E-04	9.70E-05	1.51E-01	5.74E-01	6.15	NUBP2

IGF1	4060332	IGF1	1.66E-04	1.14E-04	1.44E-01	5.74E-01	6.30	IGF1
IGF1	4260446	NR2E1	2.17E-04	1.52E-04	1.53E-01	5.74E-01	6.51	FOXO3
IGF1	430239	NRBP1	2.79E-04	1.94E-04	1.52E-01	5.74E-01	6.84	GCKR
IGF1	5960546	EIF2B4	-2.35E-04	1.63E-04	1.51E-01	5.74E-01	9.09	GCKR
IGFBP3	2030730	UBE2I	-1.11E-05	7.65E-06	1.46E-01	5.74E-01	9.76	NUBP2
IGF1	2940554	GSTM5	-1.54E-04	1.06E-04	1.46E-01	5.74E-01	6.17	CELSR2
IGF1	4730598	AMIGO1	1.67E-04	1.16E-04	1.51E-01	5.74E-01	6.19	CELSR2
IGF1	290332	TAF13	-1.45E-04	1.02E-04	1.55E-01	5.74E-01	6.25	CELSR2
IGF1	1300592	DNMT3A	-1.96E-04	1.38E-04	1.56E-01	5.74E-01	6.55	ASXL2
IGF1	2230377	TELO2	1.85E-04	1.36E-04	1.76E-01	5.78E-01	6.42	NUBP2
IGF1	4200209	RBKS	1.97E-04	1.48E-04	1.82E-01	5.78E-01	6.48	GCKR
IGF1	4490097	SPATA16	1.47E-04	1.10E-04	1.82E-01	5.78E-01	6.10	GHSR
IGF1	4610201	SNORA10	-3.61E-04	2.60E-04	1.65E-01	5.78E-01	7.25	NUBP2
IGF1	4920138	FOXO3	-1.46E-04	1.09E-04	1.80E-01	5.78E-01	6.11	FOXO3
IGF1	5260162	FOXO3	1.73E-04	1.29E-04	1.78E-01	5.78E-01	6.17	FOXO3
IGF1	6860528	CASKIN1	-1.49E-04	1.07E-04	1.64E-01	5.78E-01	6.12	NUBP2
IGFBP3	1510468	GRPEL1	-1.04E-05	7.80E-06	1.82E-01	5.78E-01	7.85	SORCS2
IGFBP3	290392	RNPS1	-1.12E-05	8.32E-06	1.80E-01	5.78E-01	7.18	NUBP2
IGFBP3	6840372	IGFBP3	-7.50E-06	5.49E-06	1.72E-01	5.78E-01	6.22	IGFBP3
IGFBP3	6940050	RNF151	-7.08E-06	5.05E-06	1.62E-01	5.78E-01	6.02	NUBP2
IGFBP3	7210397	GNPTG	-1.11E-05	8.15E-06	1.74E-01	5.78E-01	7.52	NUBP2
IGF1	1710577	GPR61	1.70E-04	1.21E-04	1.60E-01	5.78E-01	6.08	CELSR2
IGFBP3	1230577	GNAT2	7.70E-06	5.77E-06	1.83E-01	5.78E-01	6.28	CELSR2
IGF1	650075	ASXL2	2.75E-04	2.08E-04	1.86E-01	5.84E-01	9.11	ASXL2
IGF1	2060088	CLCN7	-2.71E-04	2.15E-04	2.07E-01	6.17E-01	9.93	NUBP2
IGF1	3870241	DNAJC5G	-1.64E-04	1.30E-04	2.08E-01	6.17E-01	6.17	GCKR
IGF1	6060288	PKD1	-1.37E-04	1.09E-04	2.08E-01	6.17E-01	6.40	NUBP2
IGF1	6580110	PAH	-1.27E-04	1.01E-04	2.10E-01	6.17E-01	6.11	IGF1
IGF1	7040544	CCDC53	-2.45E-04	1.92E-04	2.01E-01	6.17E-01	8.10	IGF1
IGFBP3	5890382	PTX4	-7.80E-06	6.12E-06	2.03E-01	6.17E-01	6.28	NUBP2
IGF1	1070292	GPSM2	1.62E-04	1.26E-04	1.99E-01	6.17E-01	6.40	CELSR2
IGF1	2680438	C2ORF28	-2.15E-04	1.75E-04	2.19E-01	6.26E-01	9.24	GCKR
IGF1	6220026	MAPRE3	2.02E-04	1.63E-04	2.16E-01	6.26E-01	6.89	GCKR
IGFBP3	430437	HN1L	7.73E-06	6.29E-06	2.20E-01	6.26E-01	6.18	NUBP2
IGFBP3	3130605	AKNAD1	6.08E-06	4.97E-06	2.21E-01	6.26E-01	6.15	CELSR2
IGFBP3	2600240	RAB26	6.38E-06	5.34E-06	2.32E-01	6.53E-01	6.22	NUBP2
IGF1	1850075	AGBL5	2.14E-04	1.82E-04	2.39E-01	6.59E-01	7.00	GCKR
IGF1	4230301	PGP	1.71E-04	1.45E-04	2.39E-01	6.59E-01	6.66	NUBP2
IGFBP3	4230739	SORCS2	-5.55E-06	4.78E-06	2.46E-01	6.71E-01	5.97	SORCS2
IGFBP3	4490735	KIAA0232	1.10E-05	9.61E-06	2.51E-01	6.71E-01	7.00	SORCS2
IGFBP3	6110307	MRPS34	-6.03E-06	5.25E-06	2.51E-01	6.71E-01	6.23	NUBP2
IGFBP3	650563	FAHD1	1.12E-05	9.75E-06	2.49E-01	6.71E-01	6.68	NUBP2

IGF1	5340220	MYBPHL	-1.17E-04	1.02E-04	2.54E-01	6.73E-01	6.03	CELSR2
IGF1	3440601	CLCC1	-1.17E-04	1.03E-04	2.57E-01	6.75E-01	6.13	CELSR2
IGF1	4230047	NOXO1	-1.26E-04	1.12E-04	2.61E-01	6.76E-01	6.35	NUBP2
IGF1	5690553	HADHA	-2.14E-04	1.91E-04	2.62E-01	6.76E-01	6.82	ASXL2
IGF1	3710240	CELSR2	-1.12E-04	1.00E-04	2.64E-01	6.76E-01	6.04	CELSR2
IGF1	1510152	PSRC1	1.52E-04	1.36E-04	2.66E-01	6.76E-01	6.08	CELSR2
IGFBP3	2230377	TELO2	7.04E-06	6.39E-06	2.71E-01	6.84E-01	6.42	NUBP2
IGF1	3310400	IGF1	1.07E-04	9.80E-05	2.77E-01	6.93E-01	6.11	IGF1
IGFBP3	2060088	CLCN7	-1.07E-05	1.01E-05	2.88E-01	7.15E-01	9.93	NUBP2
IGFBP3	2630022	RNPS1	-8.96E-06	8.50E-06	2.92E-01	7.20E-01	9.00	NUBP2
IGF1	6370360	RNF151	-1.11E-04	1.06E-04	2.95E-01	7.21E-01	6.06	NUBP2
IGF1	1580202	ZNF598	1.15E-04	1.11E-04	2.99E-01	7.27E-01	6.23	NUBP2
IGFBP3	1820482	NDUFB10	1.36E-05	1.32E-05	3.05E-01	7.36E-01	8.26	NUBP2
IGFBP3	5090592	FAHD1	-6.23E-06	6.16E-06	3.11E-01	7.45E-01	6.08	NUBP2
IGF1	2030730	UBE2I	-1.64E-04	1.63E-04	3.16E-01	7.50E-01	9.76	NUBP2
IGF1	3400050	TBL3	-1.47E-04	1.49E-04	3.25E-01	7.62E-01	6.45	NUBP2
IGF1	620113	ABHD1	-1.16E-04	1.18E-04	3.26E-01	7.62E-01	6.15	GCKR
IGFBP3	6370360	RNF151	4.87E-06	4.98E-06	3.28E-01	7.62E-01	6.06	NUBP2
IGFBP3	20630	C1ORF194	4.48E-06	4.60E-06	3.31E-01	7.63E-01	6.08	CELSR2
IGF1	580270	NOXO1	-1.05E-04	1.08E-04	3.33E-01	7.64E-01	6.14	NUBP2
IGF1	2100079	ECI1	-1.67E-04	1.76E-04	3.43E-01	7.64E-01	6.55	NUBP2
IGF1	3940138	PKD1	-1.76E-04	1.85E-04	3.43E-01	7.64E-01	6.93	NUBP2
IGF1	5860279	RAB10	2.23E-04	2.32E-04	3.36E-01	7.64E-01	9.81	ASXL2
IGFBP3	2710187	ATXN7L2	6.63E-06	6.98E-06	3.42E-01	7.64E-01	6.55	CELSR2
IGF1	6760719	EMILIN1	-9.35E-05	1.01E-04	3.56E-01	7.88E-01	6.23	GCKR
IGFBP3	1710577	GPR61	-5.15E-06	5.69E-06	3.66E-01	8.04E-01	6.08	CELSR2
IGF1	2570546	UBE2I	1.20E-04	1.35E-04	3.73E-01	8.11E-01	6.31	NUBP2
IGFBP3	2940554	GSTM5	-4.41E-06	4.96E-06	3.74E-01	8.11E-01	6.17	CELSR2
IGF1	6130131	DNMT3A	1.09E-04	1.23E-04	3.77E-01	8.12E-01	6.11	ASXL2
IGFBP3	1580202	ZNF598	4.54E-06	5.18E-06	3.81E-01	8.15E-01	6.23	NUBP2
IGFBP3	3990408	TRAF7	-4.43E-06	5.16E-06	3.90E-01	8.30E-01	6.10	NUBP2
IGF1	1400291	no GeneName	8.97E-05	1.11E-04	4.17E-01	8.32E-01	6.31	FOXO3
IGF1	150162	FNDC4	-7.46E-05	9.49E-05	4.32E-01	8.32E-01	6.24	GCKR
IGF1	290392	RNPS1	-1.49E-04	1.78E-04	4.01E-01	8.32E-01	7.18	NUBP2
IGF1	3870671	SUPT7L	-8.55E-05	1.07E-04	4.23E-01	8.32E-01	6.15	GCKR
IGF1	4150541	CCDC121	9.55E-05	1.20E-04	4.26E-01	8.32E-01	6.21	GCKR
IGF1	450376	TSC2	1.31E-04	1.66E-04	4.31E-01	8.32E-01	6.61	NUBP2
IGF1	5270575	MAPK8IP3	1.46E-04	1.82E-04	4.22E-01	8.32E-01	8.62	NUBP2
IGF1	6110307	MRPS34	8.84E-05	1.12E-04	4.31E-01	8.32E-01	6.23	NUBP2
IGFBP3	3390068	E4F1	5.93E-06	7.35E-06	4.20E-01	8.32E-01	8.51	NUBP2
IGFBP3	430402	ABCA3	3.94E-06	4.86E-06	4.18E-01	8.32E-01	6.13	NUBP2
IGF1	2680039	CLCC1	-7.80E-05	9.93E-05	4.33E-01	8.32E-01	6.09	CELSR2

IGF1	7150598	GSTM3	1.83E-04	2.28E-04	4.22E-01	8.32E-01	6.53	CELSR2
IGFBP3	1070292	GPSM2	-4.99E-06	5.91E-06	3.99E-01	8.32E-01	6.40	CELSR2
IGFBP3	290332	TAF13	-3.92E-06	4.78E-06	4.12E-01	8.32E-01	6.25	CELSR2
IGFBP3	4150553	TAF13	4.02E-06	5.00E-06	4.22E-01	8.32E-01	6.19	CELSR2
IGFBP3	70296	AMPD2	-4.30E-06	5.35E-06	4.22E-01	8.32E-01	6.13	CELSR2
IGFBP3	2030070	ZNF598	-6.63E-06	8.51E-06	4.36E-01	8.33E-01	8.17	NUBP2
IGF1	6940689	GFER	1.52E-04	1.97E-04	4.39E-01	8.35E-01	6.10	NUBP2
IGFBP3	3290368	IGFBP1	-1.11E-05	1.45E-05	4.45E-01	8.40E-01	7.07	IGFBP3
IGF1	6450139	FTH1P3	2.45E-04	3.23E-04	4.47E-01	8.40E-01	9.34	GCKR
IGF1	7040148	WDR47	1.15E-04	1.55E-04	4.56E-01	8.47E-01	6.90	CELSR2
IGFBP3	1070762	PSRC1	-5.57E-06	7.45E-06	4.55E-01	8.47E-01	6.58	CELSR2
IGF1	1400743	PMCH	-8.50E-05	1.16E-04	4.65E-01	8.49E-01	6.31	IGF1
IGF1	7610201	SNX3	-7.79E-05	1.06E-04	4.61E-01	8.49E-01	6.22	FOXO3
IGFBP3	2970411	SLC9A3R2	-3.49E-06	4.76E-06	4.63E-01	8.49E-01	6.07	NUBP2
IGF1	6290050	no GeneName	8.57E-05	1.19E-04	4.72E-01	8.56E-01	6.29	CELSR2
IGF1	5050022	KHK	-7.91E-05	1.11E-04	4.76E-01	8.59E-01	6.28	GCKR
IGF1	1240612	MAPK8IP3	7.54E-05	1.12E-04	5.00E-01	8.67E-01	6.02	NUBP2
IGF1	2370414	C16ORF42	1.05E-04	1.56E-04	5.02E-01	8.67E-01	6.46	NUBP2
IGF1	3190746	PAH	-7.61E-05	1.12E-04	4.97E-01	8.67E-01	6.17	IGF1
IGF1	3390068	E4F1	-1.06E-04	1.57E-04	4.99E-01	8.67E-01	8.51	NUBP2
IGF1	6450048	CCDC121	-7.71E-05	1.13E-04	4.94E-01	8.67E-01	6.29	GCKR
IGF1	1230577	GNAT2	8.50E-05	1.23E-04	4.90E-01	8.67E-01	6.28	CELSR2
IGFBP3	1170474	AMPD2	-4.30E-06	6.39E-06	5.01E-01	8.67E-01	6.00	CELSR2
IGFBP3	6020093	SARS	6.71E-06	9.77E-06	4.92E-01	8.67E-01	6.68	CELSR2
IGFBP3	3400682	RNPS1	-4.16E-06	6.35E-06	5.13E-01	8.81E-01	6.51	NUBP2
IGF1	630427	KHK	7.91E-05	1.25E-04	5.27E-01	8.87E-01	6.43	GCKR
IGF1	7210327	NCEH1	1.11E-04	1.75E-04	5.27E-01	8.87E-01	6.87	GHSR
IGFBP3	3190445	UBE2I	-3.64E-06	5.76E-06	5.27E-01	8.87E-01	6.31	NUBP2
IGFBP3	3710184	EPS8L3	2.87E-06	4.48E-06	5.22E-01	8.87E-01	6.19	CELSR2
IGF1	1470538	ZNF513	-6.58E-05	1.12E-04	5.57E-01	8.92E-01	6.41	GCKR
IGF1	2640437	GTF3C2	1.16E-04	1.88E-04	5.36E-01	8.92E-01	6.74	GCKR
IGF1	540164	KHK	6.32E-05	1.09E-04	5.61E-01	8.92E-01	6.21	GCKR
IGF1	6040255	TRIM54	-6.65E-05	1.15E-04	5.63E-01	8.92E-01	6.21	GCKR
IGF1	7560327	GPN1	-9.36E-05	1.53E-04	5.42E-01	8.92E-01	8.11	GCKR
IGF1	870202	TNFSF10	1.69E-04	2.90E-04	5.60E-01	8.92E-01	9.93	GHSR
IGF1	940202	PPM1G	6.79E-05	1.12E-04	5.46E-01	8.92E-01	6.14	GCKR
IGFBP3	2230598	IGFALS	-2.89E-06	4.81E-06	5.48E-01	8.92E-01	6.17	NUBP2
IGFBP3	2570546	UBE2I	3.75E-06	6.34E-06	5.54E-01	8.92E-01	6.31	NUBP2
IGFBP3	6860528	CASKIN1	2.89E-06	5.04E-06	5.66E-01	8.92E-01	6.12	NUBP2
IGF1	520609	KIAA1324	-2.77E-04	4.86E-04	5.69E-01	8.92E-01	7.63	CELSR2
IGF1	6550279	GSTM2	1.03E-04	1.73E-04	5.53E-01	8.92E-01	6.59	CELSR2
IGFBP3	5340220	MYBPHL	-2.97E-06	4.80E-06	5.36E-01	8.92E-01	6.03	CELSR2

IGFBP3	6940132	AMPD2	-2.99E-06	5.25E-06	5.69E-01	8.92E-01	6.09	CELSR2
IGF1	130070	IFT172	4.55E-05	1.04E-04	6.60E-01	8.97E-01	6.29	GCKR
IGF1	1570035	AGBL5	5.25E-05	1.03E-04	6.10E-01	8.97E-01	6.15	GCKR
IGF1	1710152	ARMC2	-5.71E-05	1.10E-04	6.04E-01	8.97E-01	6.05	FOXO3
IGF1	2070184	NTHL1	-6.89E-05	1.51E-04	6.49E-01	8.97E-01	6.34	NUBP2
IGF1	2350706	TRAF7	-9.54E-05	1.74E-04	5.83E-01	8.97E-01	6.87	NUBP2
IGF1	2450202	KIF3C	-5.08E-05	1.03E-04	6.22E-01	8.97E-01	6.14	ASXL2
IGF1	2600240	RAB26	-5.47E-05	1.14E-04	6.31E-01	8.97E-01	6.22	NUBP2
IGF1	290435	KRTCAP3	6.06E-05	1.39E-04	6.63E-01	8.97E-01	6.21	GCKR
IGF1	2970411	SLC9A3R2	-4.44E-05	1.01E-04	6.62E-01	8.97E-01	6.07	NUBP2
IGF1	3400682	RNPS1	-5.99E-05	1.35E-04	6.58E-01	8.97E-01	6.51	NUBP2
IGF1	5290451	UCN	5.31E-05	1.03E-04	6.07E-01	8.97E-01	6.27	GCKR
IGF1	5340431	TCF23	4.38E-05	9.96E-05	6.60E-01	8.97E-01	6.15	GCKR
IGF1	6200692	FOXO3	-1.50E-04	2.89E-04	6.04E-01	8.97E-01	11.35	FOXO3
IGF1	650563	FAHD1	9.25E-05	2.08E-04	6.57E-01	8.97E-01	6.68	NUBP2
IGF1	6620403	AGBL5	-6.34E-05	1.42E-04	6.55E-01	8.97E-01	6.69	GCKR
IGF1	6940050	RNF151	-5.54E-05	1.08E-04	6.08E-01	8.97E-01	6.02	NUBP2
IGF1	6960376	IFT140	-6.06E-05	1.39E-04	6.64E-01	8.97E-01	6.31	NUBP2
IGF1	6960730	NUBP2	-5.46E-05	1.25E-04	6.62E-01	8.97E-01	6.36	NUBP2
IGF1	7000035	HS3ST6	7.12E-05	1.29E-04	5.82E-01	8.97E-01	6.22	NUBP2
IGF1	7040689	CGREF1	-5.34E-05	1.20E-04	6.57E-01	8.97E-01	6.21	GCKR
IGFBP3	20338	NOXO1	-4.05E-06	8.12E-06	6.18E-01	8.97E-01	6.24	NUBP2
IGFBP3	2370068	UBE2I	-2.62E-06	5.13E-06	6.10E-01	8.97E-01	6.07	NUBP2
IGFBP3	2370414	C16ORF42	-3.37E-06	7.34E-06	6.46E-01	8.97E-01	6.46	NUBP2
IGFBP3	3400050	TBL3	-3.24E-06	7.01E-06	6.44E-01	8.97E-01	6.45	NUBP2
IGFBP3	450376	TSC2	-4.17E-06	7.77E-06	5.92E-01	8.97E-01	6.61	NUBP2
IGFBP3	5860609	SYNGR3	2.15E-06	5.07E-06	6.72E-01	8.97E-01	6.20	NUBP2
IGFBP3	6060288	PKD1	-2.64E-06	5.10E-06	6.05E-01	8.97E-01	6.40	NUBP2
IGFBP3	6290097	UNKL	3.07E-06	5.54E-06	5.79E-01	8.97E-01	6.35	NUBP2
IGFBP3	780719	BAIAP3	2.51E-06	4.97E-06	6.14E-01	8.97E-01	6.13	NUBP2
IGF1	2710187	ATXN7L2	7.43E-05	1.49E-04	6.18E-01	8.97E-01	6.55	CELSR2
IGF1	3610139	GSTM1	-2.59E-04	5.16E-04	6.16E-01	8.97E-01	6.98	CELSR2
IGFBP3	1510152	PSRC1	-2.93E-06	6.40E-06	6.47E-01	8.97E-01	6.08	CELSR2
IGFBP3	2680039	CLCC1	2.60E-06	4.66E-06	5.78E-01	8.97E-01	6.09	CELSR2
IGFBP3	4730598	AMIGO1	-2.59E-06	5.47E-06	6.36E-01	8.97E-01	6.19	CELSR2
IGFBP3	5570594	STXBP3	-4.63E-06	1.09E-05	6.71E-01	8.97E-01	7.71	CELSR2
IGFBP3	7040148	WDR47	-3.12E-06	7.25E-06	6.67E-01	8.97E-01	6.90	CELSR2
IGF1	1400450	BRE	-5.43E-05	1.69E-04	7.48E-01	9.18E-01	7.80	GCKR
IGF1	1470678	EME2	-5.67E-05	1.50E-04	7.06E-01	9.18E-01	6.23	NUBP2
IGF1	1710707	C2ORF16	4.62E-05	1.26E-04	7.13E-01	9.18E-01	6.30	GCKR
IGF1	1780356	HAGH	1.70E-04	5.20E-04	7.43E-01	9.18E-01	8.89	NUBP2
IGF1	1990450	CEP57L1	3.50E-05	1.04E-04	7.37E-01	9.18E-01	6.22	FOXO3

IGF1	2060274	PREB	-6.78E-05	1.82E-04	7.09E-01	9.18E-01	6.90	GCKR
IGF1	2370068	UBE2I	-3.95E-05	1.09E-04	7.18E-01	9.18E-01	6.07	NUBP2
IGF1	2760601	C16ORF73	3.67E-05	1.05E-04	7.26E-01	9.18E-01	6.19	NUBP2
IGF1	3360402	CAD	-4.45E-05	1.17E-04	7.04E-01	9.18E-01	6.26	GCKR
IGF1	4280482	DRAM1	7.16E-05	2.04E-04	7.25E-01	9.18E-01	7.52	IGF1
IGF1	4490475	SLC30A3	-6.18E-05	1.65E-04	7.08E-01	9.18E-01	6.56	GCKR
IGF1	5340066	CRAMP1L	-5.67E-05	1.72E-04	7.41E-01	9.18E-01	6.98	NUBP2
IGF1	7650020	NCEH1	-6.13E-05	1.60E-04	7.01E-01	9.18E-01	6.69	GHSR
IGFBP3	2850433	SPSB3	3.30E-06	8.46E-06	6.97E-01	9.18E-01	9.59	NUBP2
IGFBP3	6590132	IGFBP3	-2.24E-06	6.82E-06	7.42E-01	9.18E-01	6.24	IGFBP3
IGF1	1170474	AMPD2	-4.60E-05	1.36E-04	7.36E-01	9.18E-01	6.00	CELSR2
IGF1	160019	SORT1	8.16E-05	2.41E-04	7.35E-01	9.18E-01	7.69	CELSR2
IGF1	3130241	TMEM167B	6.43E-05	1.99E-04	7.46E-01	9.18E-01	8.32	CELSR2
IGF1	5910048	EPS8L3	-4.78E-05	1.36E-04	7.25E-01	9.18E-01	6.13	CELSR2
IGF1	70296	AMPD2	-4.23E-05	1.14E-04	7.11E-01	9.18E-01	6.13	CELSR2
IGFBP3	6290050	no GeneName	1.93E-06	5.59E-06	7.30E-01	9.18E-01	6.29	CELSR2
IGF1	450161	FAHD1	6.95E-05	2.31E-04	7.64E-01	9.21E-01	6.95	NUBP2
IGF1	6450373	DNMT3A	3.33E-05	1.09E-04	7.61E-01	9.21E-01	6.15	ASXL2
IGF1	6960615	RPL3L	-5.46E-05	1.80E-04	7.62E-01	9.21E-01	6.29	NUBP2
IGFBP3	580561	ADCY1	1.64E-06	5.41E-06	7.63E-01	9.21E-01	6.17	IGFBP3
IGFBP3	3440601	CLCC1	-1.49E-06	4.85E-06	7.59E-01	9.21E-01	6.13	CELSR2
IGF1	5090592	FAHD1	-3.77E-05	1.31E-04	7.74E-01	9.26E-01	6.08	NUBP2
IGF1	7210397	GNPTG	-4.98E-05	1.74E-04	7.75E-01	9.26E-01	7.52	NUBP2
IGF1	4060707	GSTM4;GSTM2	-3.26E-05	1.15E-04	7.77E-01	9.26E-01	6.23	CELSR2
IGFBP3	1710332	FAHD1	1.34E-06	5.02E-06	7.89E-01	9.32E-01	6.17	NUBP2
IGFBP3	6550279	GSTM2	-2.16E-06	8.12E-06	7.90E-01	9.32E-01	6.59	CELSR2
IGFBP3	7550138	SYPL2	1.43E-06	5.32E-06	7.88E-01	9.32E-01	6.06	CELSR2
IGF1	20524	C2ORF53	2.86E-05	1.09E-04	7.93E-01	9.32E-01	6.35	GCKR
IGF1	3850239	TRIM54	-2.76E-05	1.07E-04	7.98E-01	9.33E-01	6.07	GCKR
IGF1	1240553	SESN1	4.37E-05	2.48E-04	8.60E-01	9.42E-01	7.56	FOXO3
IGF1	2230598	IGFALS	2.16E-05	1.03E-04	8.33E-01	9.42E-01	6.17	NUBP2
IGF1	3460068	TRIM54	2.38E-05	1.41E-04	8.66E-01	9.42E-01	6.41	GCKR
IGF1	4900333	HAGH	1.02E-04	4.63E-04	8.25E-01	9.42E-01	10.69	NUBP2
IGF1	5050040	SLC5A6	2.96E-05	1.55E-04	8.48E-01	9.42E-01	7.00	GCKR
IGF1	5860609	SYNGR3	2.43E-05	1.08E-04	8.22E-01	9.42E-01	6.20	NUBP2
IGF1	5910528	DNASE1L2	-1.91E-05	1.13E-04	8.65E-01	9.42E-01	6.28	NUBP2
IGF1	6130324	ARMC2	-2.65E-05	1.25E-04	8.31E-01	9.42E-01	6.45	FOXO3
IGF1	6200243	PGP	-3.24E-05	1.61E-04	8.41E-01	9.42E-01	6.59	NUBP2
IGF1	6290097	UNKL	-2.61E-05	1.18E-04	8.25E-01	9.42E-01	6.35	NUBP2
IGF1	6590025	UCN	2.57E-05	1.29E-04	8.42E-01	9.42E-01	6.12	GCKR
IGFBP3	1440243	SEPX1	2.32E-06	1.17E-05	8.43E-01	9.42E-01	12.70	NUBP2
IGFBP3	6960376	IFT140	1.20E-06	6.53E-06	8.55E-01	9.42E-01	6.31	NUBP2

IGFBP3	6960615	RPL3L	1.93E-06	8.44E-06	8.19E-01	9.42E-01	6.29	NUBP2
IGFBP3	6960730	NUBP2	1.02E-06	5.86E-06	8.61E-01	9.42E-01	6.36	NUBP2
IGFBP3	7160707	TBC1D14	-2.02E-06	1.07E-05	8.50E-01	9.42E-01	9.71	SORCS2
IGFBP3	7210037	IGFBP1	-1.03E-06	4.68E-06	8.26E-01	9.42E-01	6.15	IGFBP3
IGF1	3130605	AKNAD1	2.14E-05	1.06E-04	8.40E-01	9.42E-01	6.15	CELSR2
IGF1	3710184	EPS8L3	-1.65E-05	9.55E-05	8.63E-01	9.42E-01	6.19	CELSR2
IGFBP3	3610139	GSTM1	5.46E-06	2.42E-05	8.22E-01	9.42E-01	6.98	CELSR2
IGFBP3	6900592	GSTM4	-1.34E-06	6.80E-06	8.43E-01	9.42E-01	6.21	CELSR2
IGF1	5860608	OST4	-4.03E-05	2.61E-04	8.77E-01	9.43E-01	9.61	GCKR
IGF1	6100523	SNX3	3.69E-05	2.41E-04	8.79E-01	9.43E-01	11.09	FOXO3
IGF1	780719	BAIAP3	1.67E-05	1.06E-04	8.75E-01	9.43E-01	6.13	NUBP2
IGFBP3	50672	GSTM1	-2.42E-06	1.52E-05	8.73E-01	9.43E-01	6.54	CELSR2
IGF1	3190445	UBE2I	-1.40E-05	1.23E-04	9.10E-01	9.45E-01	6.31	NUBP2
IGF1	3990408	TRAF7	-1.22E-05	1.10E-04	9.12E-01	9.45E-01	6.10	NUBP2
IGF1	4060292	BRE	-2.58E-05	1.78E-04	8.85E-01	9.45E-01	7.08	GCKR
IGF1	4890056	C16ORF91	-2.32E-05	1.71E-04	8.92E-01	9.45E-01	7.26	NUBP2
IGF1	6380326	EIF2B4	-1.22E-05	1.07E-04	9.10E-01	9.45E-01	6.25	GCKR
IGF1	770504	DTNB	1.19E-05	1.02E-04	9.07E-01	9.45E-01	6.30	ASXL2
IGFBP3	1470678	EME2	8.16E-07	7.04E-06	9.08E-01	9.45E-01	6.23	NUBP2
IGFBP3	4230047	NOXO1	-5.75E-07	5.24E-06	9.13E-01	9.45E-01	6.35	NUBP2
IGFBP3	60220	C16ORF79	8.87E-07	6.73E-06	8.95E-01	9.45E-01	6.58	NUBP2
IGFBP3	7570671	UNKL	-1.14E-06	8.68E-06	8.96E-01	9.45E-01	6.85	NUBP2
IGF1	6020093	SARS	-2.92E-05	2.09E-04	8.89E-01	9.45E-01	6.68	CELSR2
IGF1	20630	C1ORF194	9.59E-06	9.82E-05	9.22E-01	9.52E-01	6.08	CELSR2
IGFBP3	3130286	no GeneName	-3.96E-07	4.89E-06	9.35E-01	9.62E-01	6.15	CELSR2
IGFBP3	6940689	GFER	-6.77E-07	9.24E-06	9.42E-01	9.65E-01	6.10	NUBP2
IGFBP3	5910048	EPS8L3	-3.82E-07	6.36E-06	9.52E-01	9.73E-01	6.13	CELSR2
IGFBP3	4670463	CCDC96	-2.10E-07	4.76E-06	9.65E-01	9.80E-01	6.27	SORCS2
IGF1	50672	GSTM1	-1.48E-05	3.24E-04	9.64E-01	9.80E-01	6.54	CELSR2
IGF1	2850433	SPSB3	7.09E-06	1.80E-04	9.69E-01	9.81E-01	9.59	NUBP2
IGF1	1820482	NDUFB10	-2.36E-06	2.82E-04	9.93E-01	9.96E-01	8.26	NUBP2
IGF1	430437	HN1L	1.57E-06	1.34E-04	9.91E-01	9.96E-01	6.18	NUBP2
IGF1	5890382	PTX4	-1.84E-06	1.31E-04	9.89E-01	9.96E-01	6.28	NUBP2
IGFBP3	2350706	TRAF7	-2.59E-08	8.16E-06	9.97E-01	9.97E-01	6.87	NUBP2

Results are ordered by the association p-value.
Results with a FDR < 0.05 are marked in bold.

Supplementary Table 8: Results of pQTL analysis in the SHIP-TREND cohort

SNP	peptide sequence / protein	pQTL beta	pQTL SE	pQTL p-value	FDR	N total	uniProt Identifier	SNP chr	SNP position	Effect Allele	Other Allele	Effect Allele Frequency	SNP Imputation Quality	GWAS Locus
rs11644716	AFWLDVSHNR	-0.147	0.040	2.81E-04	**0.009**	197	ALS_HUMAN	16	1,802,928	T	C	0.96	0.70	NUBP2
rs11644716	ELVLAGNR	-0.136	0.042	1.41E-03	**0.014**	197	ALS_HUMAN	16	1,802,928	T	C	0.96	0.70	NUBP2
rs11644716	TFTPQPPGLER	-0.128	0.040	1.43E-03	**0.014**	197	ALS_HUMAN	16	1,802,928	T	C	0.96	0.70	NUBP2
rs11644716	NLPEQVFR	-0.161	0.051	1.75E-03	**0.014**	197	ALS_HUMAN	16	1,802,928	T	C	0.96	0.70	NUBP2
rs11644716	LEALPNSLLAPLGR	-0.106	0.036	3.85E-03	**0.024**	197	ALS_HUMAN	16	1,802,928	T	C	0.96	0.70	NUBP2
rs11644716	ANVFVQLPR	-0.116	0.041	5.24E-03	**0.024**	197	ALS_HUMAN	16	1,802,928	T	C	0.96	0.70	NUBP2
rs11644716	WLDLSHNR	-0.110	0.039	5.26E-03	**0.024**	197	ALS_HUMAN	16	1,802,928	T	C	0.96	0.70	NUBP2
rs11644716	DFALQNPSAVPR	-0.118	0.044	8.71E-03	**0.033**	197	ALS_HUMAN	16	1,802,928	T	C	0.96	0.70	NUBP2
rs1065656	ANVFVQLPR	0.040	0.015	9.40E-03	**0.033**	197	ALS_HUMAN	16	1,778,837	G	C	0.66	0.99	NUBP2
rs1065656	TFTPQPPGLER	0.037	0.015	1.31E-02	**0.042**	197	ALS_HUMAN	16	1,778,837	G	C	0.66	0.99	NUBP2
rs1065656	LEALPNSLLAPLGR	0.033	0.013	1.44E-02	**0.042**	197	ALS_HUMAN	16	1,778,837	G	C	0.66	0.99	NUBP2
rs1065656	DFALQNPSAVPR	0.039	0.016	1.75E-02	**0.047**	197	ALS_HUMAN	16	1,778,837	G	C	0.66	0.99	NUBP2
rs11977526	YGQPLPGYTTK	-0.049	0.021	2.37E-02	0.058	197	IBP3_HUMAN	7	45,974,635	G	A	0.61	0.99	IGFBP3
rs1065656	AFWLDVSHNR	0.033	0.015	2.86E-02	0.065	197	ALS_HUMAN	16	1,778,837	G	C	0.66	0.99	NUBP2
rs11644716	NLIAAVAPGAFLGLK	-0.091	0.042	3.18E-02	0.065	197	ALS_HUMAN	16	1,802,928	T	C	0.96	0.70	NUBP2
rs1065656	LAYLQPALFSGLAELR	0.034	0.016	3.27E-02	0.065	197	ALS_HUMAN	16	1,778,837	G	C	0.66	0.99	NUBP2
rs11644716	SLALGTFAHTPALASLGLSNNR	-0.126	0.060	3.86E-02	0.073	196	ALS_HUMAN	16	1,802,928	T	C	0.96	0.70	NUBP2
rs11644716	LAYLQPALFSGLAELR	-0.087	0.043	4.41E-02	0.078	197	ALS_HUMAN	16	1,802,928	T	C	0.96	0.70	NUBP2
rs1065656	SLALGTFAHTPALASLGLSNNR	0.043	0.022	5.36E-02	0.086	196	ALS_HUMAN	16	1,778,837	G	C	0.66	0.99	NUBP2
rs1065656	VAGLLEDTFPGLLGLR	0.062	0.032	5.47E-02	0.086	197	ALS_HUMAN	16	1,778,837	G	C	0.66	0.99	NUBP2
rs11977526	ALAQCAPPPAVCAELVR	-0.048	0.025	5.64E-02	0.086	188	IBP3_HUMAN	7	45,974,635	G	A	0.61	0.99	IGFBP3
rs11644716	VAGLLEDTFPGLLGLR	-0.150	0.087	8.86E-02	0.129	197	ALS_HUMAN	16	1,802,928	T	C	0.96	0.70	NUBP2
rs1065656	LWLEGNPWDCGCPLK	0.049	0.029	9.50E-02	0.132	195	ALS_HUMAN	16	1,778,837	G	C	0.66	0.99	NUBP2
rs1065656	WLDLSHNR	0.023	0.015	1.09E-01	0.145	197	ALS_HUMAN	16	1,778,837	G	C	0.66	0.99	NUBP2
rs1065656	ELVLAGNR	0.024	0.016	1.32E-01	0.169	197	ALS_HUMAN	16	1,778,837	G	C	0.66	0.99	NUBP2

rs11644716	DLHFLEELQLGHNR	-0.085	0.058	1.41E-01	0.173	197	ALS_HUMAN	16	1,802,928	T	C	0.96	0.70	NUBP2
rs11644716	LWLEGNPWDCGCPLK	-0.107	0.079	1.73E-01	0.205	195	ALS_HUMAN	16	1,802,928	T	C	0.96	0.70	NUBP2
rs1065656	LLQLLGR	-0.060	0.045	1.92E-01	0.220	176	RT34_HUMAN	16	1,778,837	G	C	0.66	0.99	NUBP2
rs1065656	NLPEQVFR	0.023	0.019	2.23E-01	0.246	197	ALS_HUMAN	16	1,778,837	G	C	0.66	0.99	NUBP2
rs1065656	NLIAAVAPGAFLGLK	0.018	0.016	2.42E-01	0.258	197	ALS_HUMAN	16	1,778,837	G	C	0.66	0.99	NUBP2
rs1065656	DLHFLEELQLGHNR	0.014	0.021	5.26E-01	0.543	197	ALS_HUMAN	16	1,778,837	G	C	0.66	0.99	NUBP2
rs780093	DIAILK	0.004	0.012	7.26E-01	0.726	197	CC121_HUMAN	2	27,596,107	T	C	0.39	1.00	GCKR
rs11644716	protein ALS	-0.120	0.037	**1.53E-03**		197	ALS_HUMAN	16	1,802,928	T	C	0.96	0.70	NUBP2
rs11977526	protein IBP3	-0.051	0.020	1.06E-02		197	IBP3_HUMAN	7	45,974,635	G	A	0.61	0.99	IGFBP3
rs1065656	protein ALS	0.033	0.014	1.86E-02		197	ALS_HUMAN	16	1,778,837	G	C	0.66	0.99	NUBP2
rs1065656	protein RT34	-0.060	0.045	1.92E-01		176	RT34_HUMAN	16	1,778,837	G	C	0.66	0.99	NUBP2
rs780093	protein CC121	0.004	0.012	7.26E-01		197	CC121_HUMAN	2	27,596,107	T	C	0.39	1.00	GCKR

Results are ordered by peptide/protein association and then by p-value.
Significant results are marked in bold.

Supplementary Table 9: Lookup of genome-wide significant lead SNPs in IGF-I/IGFBP-3 correlated traits

SNP	GWAS Locus	BMI	Height	Waist-to-hip ratio	Fat percentage	Lumbar spine BMD	Femoral neck BMD	CARDIoGRAM GWA meta-analysis	C4D GWA meta-analysis	CARDIoGRAMplusC4D Metabochip meta-analysis	longevity
rs1065656	*NUBP2*	7.43E-01	1.60E-01	8.20E-02	4.82E-01	6.38E-01	5.47E-01	2.04E-01	NA	NA	7.33E-01
rs11977526	*IGFBP3*	4.43E-01	3.25E-02	3.60E-02	**7.12E-03**	2.60E-01	1.00E+00	4.58E-01	NA	2.61E-01	2.30E-01
rs2153960	*FOXO3*	**1.80E-03**	**5.13E-05**	2.50E-01	6.87E-01	6.24E-01	3.62E-01	1.45E-01	4.95E-01	NA	**3.19E-04**
rs4234798	*SORCS2*	6.23E-01	7.31E-01	3.10E-01	3.34E-01	6.62E-02	8.19E-01	2.94E-01	2.05E-01	NA	6.39E-01
rs509035	*GHSR*	4.25E-01	**1.36E-13**	1.60E-01	2.31E-01	6.88E-01	3.01E-01	2.77E-01	NA	**4.89E-02**	5.78E-01
rs646776	*CELSR2*	6.51E-02	**7.63E-05**	2.30E-01	6.25E-01	**9.03E-03**	2.71E-01	**5.19E-08**	**7.01E-11**	**9.41E-15**	1.77E-01
rs700753	*TNS3*	3.55E-01	1.32E-01	2.30E-01	3.09E-01	1.57E-01	**2.34E-02**	5.58E-02	NA	NA	8.63E-01
rs780093	*GCKR*	8.71E-02	**1.90E-04**	**3.00E-04**	3.24E-01	**2.90E-03**	2.43E-01	1.27E-01	NA	5.82E-01	7.68E-01
rs934073	*ASXL2*	**8.20E-03**	1.02E-01	5.70E-02	**9.37E-05**	**4.26E-03**	3.45E-01	5.66E-01	NA	NA	**1.81E-02**
rs978458	*IGF1*	5.22E-01	1.88E-01	1.50E-02	2.67E-01	5.83E-01	1.71E-01	1.22E-01	2.74E-01	**3.71E-02**	5.18E-01

SNP	GWAS Locus	type 2 diabetes (GWAS)	type 2 diabetes (MetaboChip)	Fasting glucose	2-hour glucose	HbA1c	Fasting insulin	Proinsulin	HOMA-IR	HOMA-B
rs1065656	*NUBP2*	8.50E-01	NA	1.92E-01	7.48E-02	4.44E-01	6.58E-01	1.53E-01	8.05E-01	7.65E-01
rs11977526	*IGFBP3*	**1.90E-02**	1.57E-01	5.83E-01	2.21E-01	5.79E-01	3.07E-01	4.19E-01	1.03E-01	2.68E-01
rs2153960	*FOXO3*	3.10E-01	NA	3.00E-01	9.38E-01	1.29E-01	9.93E-01	2.57E-01	8.02E-01	8.96E-01
rs4234798	*SORCS2*	3.30E-01	NA	8.82E-01	5.08E-01	8.89E-01	5.14E-01	5.67E-01	1.80E-01	4.36E-01
rs509035	*GHSR*	7.70E-01	8.72E-01	7.09E-01	3.81E-01	**1.21E-02**	3.29E-01	4.40E-01	8.34E-01	4.50E-01
rs646776	*CELSR2*	**2.40E-02**	**1.19E-02**	1.61E-01	1.66E-01	8.36E-01	2.47E-01	3.22E-01	8.98E-01	1.94E-01
rs700753	*TNS3*	**1.50E-02**	NA	6.79E-01	3.54E-01	**2.28E-04**	8.35E-01	**2.99E-02**	7.56E-01	9.56E-01
rs780093	*GCKR*	8.60E-02	**3.70E-06**	**2.91E-13**	**1.68E-06**	3.02E-01	**1.98E-04**	**4.01E-02**	3.87E-01	**1.31E-06**
rs934073	*ASXL2*	3.70E-01	NA	2.76E-01	4.22E-01	7.79E-01	1.27E-01	1.91E-01	3.12E-01	9.23E-02
rs978458	*IGF1*	1.60E-01	**2.66E-02**	5.00E-01	2.72E-01	8.98E-01	**4.74E-04**	9.64E-01	**3.71E-04**	**9.84E-05**

NA = results not available.
p-values < 0.01 are marked in bold.

References: (height, BMI, waist-to-hip ratio, and fat percentage) (Heid *et al.* 2010; Lango Allen *et al.* 2010; Speliotes *et al.* 2010), bone mineral density (Estrada *et al.* 2012), risk of type 2 diabetes (Voight *et al.* 2010; Morris *et al.* 2012) and related traits (fasting glucose, 2-hour glucose, HbA1c, fasting insulin, proinsulin, HOMA-IR, and HOMA-B) (Dupuis *et al.* 2010; Saxena *et al.* 2010; Soranzo *et al.* 2010), coronary artery disease (Coronary Artery Disease Genetics 2011; Schunkert *et al.* 2011; Consortium *et al.* 2013) and survival beyond age 90 years old (Broer *et al.* 2014).

Supplementary Table 10: Lookup of Top IGF-I associated SNPs in associations with survival beyond age 90 years old

SNP	Effect allele	Other allele	Effect allele frequency	Effect	SE	p-value	Effect direction for IGF-I
**rs10457180**	**a**	**g**	**0.70**	**-0.144**	**0.037**	**8.56E-05**	+
rs9400239	t	c	0.30	0.144	0.037	9.10E-05	-
rs13217795	t	c	0.70	-0.144	0.037	9.15E-05	+
rs4946932	a	c	0.30	0.144	0.037	9.33E-05	-
rs2022464	a	c	0.31	0.143	0.037	9.73E-05	-
rs3800227	a	g	0.26	0.146	0.039	1.61E-04	-
rs1268178	t	g	0.66	-0.153	0.041	1.75E-04	+
rs1268170	a	g	0.65	-0.132	0.035	2.07E-04	+
rs1268169	t	g	0.65	-0.131	0.036	2.10E-04	+
rs1268166	t	c	0.66	-0.132	0.036	2.16E-04	+
rs1268167	a	g	0.35	0.131	0.036	2.17E-04	-
rs1268165	t	c	0.65	-0.131	0.036	2.31E-04	+
rs1268164	a	g	0.65	-0.131	0.036	2.34E-04	+
rs1268171	c	g	0.65	-0.130	0.036	2.44E-04	+
rs2207731	a	g	0.65	-0.130	0.035	2.51E-04	+
rs9398172	a	g	0.70	-0.133	0.036	2.54E-04	+
rs1268162	a	g	0.65	-0.130	0.036	2.55E-04	+
rs1935949	a	g	0.30	0.133	0.036	2.58E-04	-
rs3800229	t	g	0.70	-0.133	0.036	2.59E-04	+
rs1935952	c	g	0.70	-0.132	0.036	2.70E-04	+
rs1159806	a	t	0.65	-0.129	0.036	2.75E-04	+
rs1268177	a	g	0.65	-0.129	0.036	2.91E-04	+
rs4946936	t	c	0.29	0.132	0.037	2.95E-04	-
rs1268179	c	g	0.35	0.129	0.036	2.99E-04	-
rs1268174	a	g	0.65	-0.129	0.036	3.18E-04	+
rs2153960	a	g	0.70	-0.130	0.036	3.19E-04	+
rs1268175	a	g	0.65	-0.128	0.036	3.29E-04	+
rs4946935	a	g	0.29	0.131	0.037	3.51E-04	-
rs9398171	t	c	0.71	-0.130	0.037	4.02E-04	+
**rs11892454**	**a**	**g**	**0.74**	**0.115**	**0.039**	**3.03E-03**	-
rs6546452	a	g	0.22	-0.103	0.040	9.02E-03	+
rs17680828	t	c	0.78	0.102	0.040	1.04E-02	-
rs10180261	t	c	0.22	-0.102	0.040	1.07E-02	+
rs934073	c	g	0.71	0.089	0.038	1.81E-02	-
rs6760135	t	c	0.23	-0.091	0.040	2.28E-02	+
rs10190878	a	g	0.69	0.080	0.037	3.17E-02	-

rs479744	t	g	0.21	0.090	0.042	3.19E-02	-
rs7564034	a	g	0.68	0.077	0.036	3.47E-02	-
rs10184492	c	g	0.68	0.076	0.036	3.61E-02	-
rs7574653	t	c	0.68	0.076	0.036	3.62E-02	-
rs9486916	t	c	0.20	0.088	0.042	3.76E-02	-
rs6711346	a	g	0.33	-0.086	0.042	4.01E-02	+
rs13004909	a	t	0.29	-0.072	0.037	5.13E-02	+
rs7605347	a	g	0.71	0.072	0.037	5.15E-02	-
rs13410396	c	g	0.29	-0.072	0.037	5.21E-02	+
rs3778588	t	c	0.17	0.087	0.045	5.21E-02	-
rs17680961	t	g	0.71	0.072	0.037	5.23E-02	-
rs531111	t	c	0.82	-0.086	0.044	5.37E-02	+
rs11888724	a	g	0.71	0.071	0.037	5.41E-02	-
rs11904454	t	c	0.29	-0.071	0.037	5.43E-02	+
rs11888512	a	g	0.71	0.071	0.037	5.43E-02	-
rs13030095	a	g	0.29	-0.069	0.037	6.05E-02	+
rs10191905	t	g	0.71	0.069	0.037	6.21E-02	-
rs10207926	a	c	0.71	0.068	0.037	6.32E-02	-
rs10174199	a	c	0.71	0.068	0.037	6.42E-02	-
rs13418205	t	c	0.71	0.068	0.037	6.42E-02	-
rs878223	a	t	0.71	0.068	0.037	6.44E-02	-
rs6758088	a	g	0.29	-0.068	0.037	6.46E-02	+
rs890680	a	g	0.71	0.068	0.037	6.51E-02	-
rs1076000	a	g	0.29	-0.068	0.037	6.57E-02	+
rs890681	a	c	0.71	0.068	0.037	6.58E-02	-
rs10153560	a	g	0.71	0.068	0.037	6.58E-02	-
rs7572629	a	g	0.71	0.068	0.037	6.59E-02	-
rs3731639	t	g	0.29	-0.068	0.037	6.65E-02	+
rs919205	t	c	0.29	-0.068	0.037	6.65E-02	+
rs10194371	a	g	0.29	-0.067	0.037	6.71E-02	+
rs10171348	t	c	0.29	-0.067	0.037	6.72E-02	+
rs12713680	a	g	0.71	0.067	0.037	6.76E-02	-
rs10180842	a	g	0.71	0.067	0.037	6.77E-02	-
rs10182811	t	c	0.29	-0.067	0.037	6.88E-02	+
rs3813498	t	c	0.81	-0.079	0.044	6.88E-02	+
rs11897276	a	g	0.71	0.067	0.037	7.00E-02	-
rs2891480	a	c	0.29	-0.067	0.037	7.07E-02	+
rs13421137	a	g	0.29	-0.066	0.037	7.19E-02	+
rs9309434	t	c	0.29	-0.066	0.037	7.26E-02	+
rs1935958	t	c	0.15	0.083	0.047	7.49E-02	-
rs13425195	t	c	0.71	0.067	0.038	7.73E-02	-
rs7560314	t	g	0.32	-0.064	0.036	7.75E-02	+

rs10469884	t	g	0.29	-0.065	0.037	7.82E-02	+
rs7599961	t	c	0.68	0.063	0.036	7.86E-02	-
rs11894959	t	c	0.32	-0.063	0.036	8.03E-02	+
rs6753192	a	c	0.32	-0.063	0.036	8.04E-02	+
rs7591614	t	c	0.32	-0.063	0.036	8.05E-02	+
rs10490569	t	c	0.68	0.063	0.036	8.08E-02	-
rs7571093	c	g	0.68	0.063	0.036	8.11E-02	-
rs13405775	t	c	0.33	-0.062	0.036	8.28E-02	+
rs13413825	a	t	0.33	-0.062	0.036	8.30E-02	+
rs2176180	t	c	0.33	-0.062	0.036	8.49E-02	+
rs10210497	t	g	0.73	0.067	0.039	8.59E-02	-
rs4063544	a	c	0.67	0.061	0.036	8.63E-02	-
rs10168718	a	g	0.33	-0.061	0.036	8.73E-02	+
rs10208202	t	c	0.67	0.061	0.036	8.83E-02	-
rs13004845	t	c	0.33	-0.061	0.036	8.85E-02	+
rs10178552	t	c	0.32	-0.060	0.036	9.30E-02	+
rs6741640	t	c	0.33	-0.060	0.036	9.49E-02	+
rs10185886	a	c	0.67	0.060	0.036	9.50E-02	-
rs11888036	a	g	0.67	0.060	0.036	9.54E-02	-
rs9480867	a	g	0.16	0.076	0.046	9.60E-02	-
rs1268161	a	g	0.16	0.075	0.045	9.68E-02	-
rs11898327	t	c	0.67	0.059	0.036	1.03E-01	-
rs6546575	c	g	0.33	-0.059	0.036	1.04E-01	+
rs9480866	t	c	0.16	0.073	0.045	1.07E-01	-
rs9486913	c	g	0.84	-0.072	0.045	1.09E-01	+
rs761779	a	g	0.16	0.072	0.045	1.13E-01	-
rs9285397	t	c	0.84	-0.068	0.045	1.29E-01	+
rs7762395	a	g	0.16	0.069	0.046	1.30E-01	-
rs9486901	t	c	0.83	-0.066	0.045	1.42E-01	+
**rs1799969**	**a**	**g**	**0.12**	**-0.077**	**0.054**	**1.58E-01**	+
rs7747393	t	c	0.16	0.062	0.045	1.71E-01	-
rs6739177	t	c	0.67	0.054	0.041	1.79E-01	-
rs7609004	t	c	0.67	0.054	0.040	1.83E-01	-
rs9480865	t	c	0.84	-0.059	0.045	1.91E-01	+
rs7753734	t	c	0.17	0.059	0.045	1.95E-01	-
rs9486902	t	c	0.16	0.058	0.045	1.98E-01	-
rs949940	a	g	0.55	0.042	0.035	2.27E-01	+
**rs11111272**	**c**	**g**	**0.71**	**-0.042**	**0.037**	**2.68E-01**	-
rs1917615	a	c	0.22	-0.046	0.043	2.93E-01	-
**rs12084451**	**a**	**t**	**0.85**	**-0.051**	**0.049**	**2.98E-01**	-
**rs4143841**	**t**	**c**	**0.50**	**0.036**	**0.035**	**3.03E-01**	+
rs5009837	t	c	0.29	0.038	0.037	3.15E-01	+

rs12145383	a	t	0.15	0.046	0.047	3.21E-01	+
rs12061205	t	c	0.15	0.046	0.047	3.24E-01	+
rs1506779	a	t	0.85	-0.046	0.047	3.25E-01	-
rs10913208	t	c	0.15	0.046	0.047	3.28E-01	+
rs10745940	a	g	0.29	0.036	0.038	3.37E-01	+
**rs11689803**	**a**	**t**	**0.28**	**0.037**	**0.040**	**3.62E-01**	+
rs7592985	a	c	0.73	0.042	0.048	3.85E-01	-
rs12094283	t	c	0.84	-0.039	0.046	3.93E-01	-
rs10193672	c	g	0.73	0.041	0.048	3.94E-01	-
**rs157930**	**t**	**c**	**0.30**	**-0.033**	**0.039**	**4.01E-01**	+
rs10753130	t	c	0.16	0.037	0.046	4.19E-01	+
rs157931	t	c	0.30	-0.031	0.039	4.23E-01	+
rs4492574	t	c	0.75	0.030	0.039	4.37E-01	-
rs12537178	a	g	0.22	-0.031	0.042	4.62E-01	-
rs972936	t	c	0.27	0.027	0.038	4.78E-01	+
**rs10860865**	**t**	**g**	**0.27**	**0.027**	**0.038**	**4.85E-01**	+
rs10020957	a	g	0.41	-0.024	0.034	4.86E-01	-
rs5742667	a	g	0.27	0.027	0.038	4.87E-01	+
rs5742678	c	g	0.27	0.026	0.038	4.99E-01	+
rs9308315	a	t	0.27	0.025	0.038	5.09E-01	+
**rs856567**	**c**	**g**	**0.57**	**0.022**	**0.034**	**5.12E-01**	+
rs978458	t	c	0.27	0.025	0.038	5.18E-01	+
rs10489477	t	c	0.15	0.029	0.047	5.46E-01	+
rs11578503	t	c	0.15	0.028	0.048	5.49E-01	+
rs11585020	a	g	0.84	-0.028	0.047	5.56E-01	-
**rs572169**	**t**	**c**	**0.31**	**0.022**	**0.037**	**5.58E-01**	+
rs4764696	t	c	0.25	0.022	0.039	5.68E-01	+
rs509035	a	g	0.31	0.020	0.037	5.78E-01	+
rs157935	t	g	0.70	0.021	0.037	5.82E-01	-
rs2373721	c	g	0.75	-0.021	0.039	5.88E-01	-
rs125124	c	g	0.70	0.020	0.038	5.90E-01	-
rs2288378	t	c	0.25	0.021	0.039	5.97E-01	+
rs4425043	a	g	0.38	-0.018	0.035	6.07E-01	+
rs17172765	c	g	0.21	-0.022	0.044	6.09E-01	-
rs5742694	a	c	0.75	-0.020	0.039	6.10E-01	-
rs856589	a	g	0.32	-0.018	0.036	6.11E-01	+
rs856540	a	g	0.37	-0.019	0.036	6.11E-01	-
rs7296464	a	g	0.75	-0.020	0.039	6.14E-01	-
rs4689088	a	g	0.61	0.017	0.034	6.14E-01	+
rs1260320	a	g	0.39	-0.018	0.035	6.16E-01	+
rs813592	t	c	0.61	0.017	0.035	6.19E-01	-
rs2293572	c	g	0.62	0.017	0.035	6.25E-01	-

rs4234797	a	g	0.61	0.017	0.034	6.26E-01	+
rs3739095	a	g	0.44	0.017	0.035	6.30E-01	+
rs2293571	a	g	0.38	-0.017	0.035	6.31E-01	+
**rs2575352**	**a**	**g**	**0.15**	**0.023**	**0.048**	**6.31E-01**	-
rs704795	a	g	0.39	-0.017	0.035	6.36E-01	+
**rs9388487**	**t**	**g**	**0.46**	**0.018**	**0.039**	**6.39E-01**	+
rs4234798	t	g	0.39	-0.016	0.034	6.39E-01	-
rs6760828	t	c	0.61	0.015	0.035	6.65E-01	-
rs780110	a	g	0.44	0.015	0.035	6.70E-01	+
rs11767621	a	g	0.79	0.019	0.046	6.72E-01	+
rs4775455	a	t	0.56	-0.016	0.039	6.74E-01	+
rs2303369	t	c	0.38	-0.014	0.035	6.80E-01	+
rs9375435	t	c	0.46	0.014	0.034	6.83E-01	+
rs1049817	a	g	0.61	0.014	0.035	6.84E-01	-
rs4665969	t	c	0.61	0.014	0.035	6.84E-01	-
rs957755	t	g	0.19	-0.018	0.044	6.89E-01	-
rs700750	a	c	0.62	0.014	0.035	6.89E-01	+
rs6743819	t	g	0.39	-0.014	0.035	6.92E-01	+
rs10205219	t	c	0.61	0.014	0.035	6.92E-01	-
rs4665978	t	c	0.56	-0.014	0.035	6.96E-01	-
rs7586601	a	g	0.56	-0.013	0.035	7.02E-01	-
rs11769597	t	c	0.81	0.016	0.044	7.08E-01	+
rs856560	t	c	0.38	-0.013	0.034	7.10E-01	-
rs856563	t	c	0.38	-0.013	0.034	7.11E-01	-
rs963024	a	g	0.59	0.013	0.035	7.20E-01	+
rs2280737	t	c	0.62	0.012	0.035	7.21E-01	-
rs780100	t	g	0.39	-0.012	0.035	7.23E-01	+
rs704791	t	c	0.61	0.012	0.035	7.24E-01	-
rs780117	c	g	0.61	0.012	0.035	7.24E-01	-
rs1060525	a	g	0.61	0.012	0.035	7.24E-01	-
rs4724487	a	g	0.73	0.013	0.038	7.24E-01	+
rs7563162	t	c	0.39	-0.012	0.035	7.25E-01	+
rs2911712	a	t	0.61	0.012	0.035	7.25E-01	-
rs7170293	a	t	0.43	-0.013	0.036	7.26E-01	-
rs12533001	t	g	0.19	-0.016	0.046	7.26E-01	-
rs6547626	t	c	0.39	-0.012	0.035	7.26E-01	+
rs4665976	a	g	0.61	0.012	0.035	7.31E-01	-
rs1728922	a	c	0.61	0.012	0.035	7.33E-01	-
rs1647266	t	c	0.61	0.012	0.035	7.33E-01	-
rs1065656	c	g	0.30	0.013	0.038	7.33E-01	-
rs4803	a	g	0.61	0.012	0.035	7.33E-01	-
rs780102	t	c	0.61	0.012	0.035	7.36E-01	-

rs1260341	a	t	0.39	-0.012	0.035	7.36E-01	+
rs2010087	t	c	0.38	-0.012	0.035	7.37E-01	+
rs1260342	t	g	0.39	-0.012	0.035	7.38E-01	+
rs157928	t	c	0.66	0.013	0.039	7.38E-01	-
rs12476704	a	c	0.38	-0.012	0.035	7.38E-01	+
rs1647276	t	c	0.39	-0.012	0.035	7.40E-01	+
rs780106	a	c	0.61	0.012	0.035	7.40E-01	-
rs12535368	a	g	0.27	-0.013	0.038	7.40E-01	-
rs7594812	a	g	0.61	0.012	0.035	7.41E-01	-
rs780104	a	g	0.39	-0.011	0.035	7.45E-01	+
rs780107	a	g	0.61	0.011	0.035	7.48E-01	-
rs1647284	t	c	0.39	-0.011	0.035	7.49E-01	+
rs4724488	a	g	0.27	-0.012	0.038	7.51E-01	-
rs17259858	t	c	0.73	0.012	0.038	7.51E-01	+
**rs2144782**	**t**	**c**	**0.37**	**-0.012**	**0.038**	**7.52E-01**	+
rs7177173	a	t	0.60	0.011	0.035	7.52E-01	+
rs780094	t	c	0.40	-0.010	0.034	7.59E-01	-
rs10229581	t	c	0.23	0.013	0.042	7.64E-01	-
rs7602534	t	c	0.38	-0.010	0.035	7.66E-01	+
rs9388486	t	c	0.55	-0.010	0.034	7.67E-01	-
rs6907898	t	c	0.45	0.010	0.034	7.67E-01	+
rs780093	t	c	0.40	-0.010	0.034	7.68E-01	-
rs7172145	t	c	0.58	0.010	0.036	7.70E-01	+
rs7166891	a	t	0.42	-0.010	0.036	7.71E-01	-
rs17172768	a	g	0.23	0.012	0.042	7.77E-01	-
rs856565	a	c	0.27	-0.011	0.038	7.77E-01	+
rs17172769	c	g	0.23	0.012	0.042	7.79E-01	-
rs1528533	c	g	0.39	-0.010	0.035	7.82E-01	+
rs856569	a	g	0.26	-0.011	0.039	7.83E-01	+
rs13472	a	g	0.38	-0.010	0.035	7.85E-01	+
rs17172767	t	c	0.77	-0.011	0.042	7.89E-01	+
rs878024	t	c	0.27	-0.010	0.038	7.93E-01	-
rs1425270	t	c	0.55	0.009	0.035	7.95E-01	+
rs1436958	t	g	0.41	-0.009	0.035	7.96E-01	-
rs856568	a	t	0.74	0.010	0.038	8.00E-01	-
rs1260333	a	g	0.46	0.009	0.034	8.04E-01	-
rs8027751	a	t	0.40	-0.009	0.035	8.06E-01	-
rs2911711	a	t	0.54	-0.008	0.035	8.08E-01	+
rs8179252	a	c	0.61	0.008	0.035	8.11E-01	-
rs7782135	t	c	0.73	0.009	0.038	8.18E-01	+
rs11681351	a	g	0.39	-0.008	0.035	8.21E-01	+
rs7738135	a	g	0.45	0.009	0.038	8.22E-01	+

rs12912208	t	c	0.47	-0.008	0.035	8.30E-01	-
rs2414755	t	c	0.42	-0.007	0.035	8.42E-01	-
rs1260326	t	c	0.41	-0.007	0.034	8.43E-01	-
rs8034914	a	g	0.60	0.007	0.035	8.45E-01	+
rs5006593	t	c	0.40	-0.007	0.035	8.45E-01	-
rs8033816	a	g	0.57	0.007	0.037	8.47E-01	+
rs949941	a	g	0.77	-0.008	0.042	8.48E-01	+
rs8029942	a	g	0.47	-0.007	0.035	8.49E-01	-
rs8034335	a	g	0.42	-0.006	0.034	8.60E-01	-
rs700753	c	g	0.35	-0.006	0.035	8.63E-01	-
rs17271340	t	c	0.41	-0.006	0.035	8.64E-01	-
rs700752	c	g	0.35	-0.006	0.035	8.69E-01	-
rs7178424	t	c	0.44	-0.006	0.035	8.72E-01	-
rs1436966	a	g	0.53	0.006	0.035	8.74E-01	+
rs7178540	a	g	0.44	-0.005	0.035	8.77E-01	-
rs12442212	a	c	0.56	0.005	0.035	8.78E-01	+
rs12439356	t	c	0.56	0.005	0.035	8.83E-01	+
rs10519157	t	g	0.59	0.005	0.035	8.84E-01	+
rs12910541	t	c	0.51	0.005	0.036	8.91E-01	+
rs7177711	a	g	0.56	0.005	0.035	8.91E-01	+
rs12442675	a	g	0.44	-0.005	0.034	8.93E-01	-
rs7178945	t	c	0.56	0.004	0.035	8.99E-01	+
rs2042608	a	c	0.45	-0.004	0.035	9.01E-01	-
rs893156	a	g	0.44	-0.004	0.035	9.02E-01	-
rs891867	a	g	0.27	-0.005	0.038	9.02E-01	-
rs1917609	t	c	0.27	-0.005	0.038	9.04E-01	-
rs7177276	t	c	0.44	-0.004	0.035	9.05E-01	-
rs8039105	t	c	0.41	-0.004	0.035	9.09E-01	-
rs12899801	a	g	0.55	0.004	0.035	9.12E-01	+
rs12593844	t	c	0.45	-0.004	0.035	9.12E-01	-
rs12439934	a	g	0.44	-0.004	0.035	9.14E-01	-
rs1490388	t	c	0.46	-0.004	0.034	9.15E-01	+
rs17587048	t	c	0.18	-0.005	0.045	9.16E-01	-
rs17205463	t	c	0.44	-0.004	0.035	9.20E-01	-
rs9398803	a	g	0.54	-0.003	0.034	9.21E-01	-
rs9375446	a	g	0.45	-0.003	0.034	9.22E-01	+
rs12594658	a	g	0.56	0.003	0.034	9.27E-01	+
rs17271438	a	c	0.44	-0.003	0.035	9.29E-01	-
rs17271431	t	c	0.44	-0.003	0.035	9.29E-01	-
rs8025877	a	t	0.56	0.003	0.035	9.29E-01	+
rs8026008	t	c	0.44	-0.003	0.035	9.29E-01	-
rs1907067	a	c	0.54	0.003	0.034	9.30E-01	-

rs893158	a	g	0.44	-0.003	0.035	9.30E-01	-
rs856549	t	c	0.26	0.003	0.039	9.31E-01	+
rs12592402	a	g	0.56	0.003	0.035	9.32E-01	+
rs11633500	a	g	0.56	0.002	0.035	9.53E-01	+
rs4775458	t	g	0.56	0.002	0.035	9.58E-01	+
rs17205407	a	g	0.44	-0.002	0.035	9.63E-01	-
rs856575	a	t	0.50	0.002	0.040	9.63E-01	-
rs12913951	a	g	0.56	0.002	0.035	9.64E-01	+
rs856541	t	c	0.25	-0.001	0.039	9.76E-01	+
rs7162536	t	g	0.56	0.001	0.035	9.76E-01	+
rs1436964	a	g	0.56	0.001	0.035	9.76E-01	+
rs866488	t	c	0.75	0.001	0.039	9.81E-01	-
rs1415671	t	g	0.54	0.001	0.034	9.81E-01	-
rs17271403	t	c	0.44	-0.001	0.035	9.83E-01	-
rs856548	a	g	0.75	0.001	0.039	9.85E-01	-
rs8039651	t	c	0.44	0.001	0.035	9.88E-01	-
rs9398808	t	g	0.46	-0.001	0.034	9.89E-01	+
rs11635977	t	c	0.56	0.000	0.035	9.93E-01	+
rs2568696	t	g	0.25	0.000	0.039	9.94E-01	+
rs9385400	t	g	0.54	0.000	0.034	9.95E-01	-
rs933807	c	g	0.44	0.000	0.034	9.96E-01	-
rs4565329	t	c	0.46	0.000	0.034	9.98E-01	+
rs1030859	t	c	0.44	0.000	0.035	9.99E-01	-
rs856554	t	c	0.75	0.000	0.039	1.00E+00	-

"-": effect allele associated with lower IGF-I levels.
"+": effect allele associated with higher IGF-I levels.
Resuts for 13 independent SNPs defined based on LD (settings r2>0.01, 1 Mb distance) are indicated by bold text.

Supplementary Table 11: Lookup of Top IGFBP-3 associated SNPs in associations with survival beyond age 90 years old

SNP	Effect allele	Other allele	Effect allele frequency	Effect	SE	p-value	Effect direction for IGFBP-3
**rs9398172**	**a**	**g**	**0.70**	**-0.133**	**0.036**	**2.54E-04**	**+**
rs1935949	a	g	0.30	0.133	0.036	2.58E-04	-
rs3800229	t	g	0.70	-0.133	0.036	2.59E-04	+
rs1935952	c	g	0.70	-0.132	0.036	2.70E-04	+
rs2153960	a	g	0.70	-0.130	0.036	3.19E-04	+
rs4946935	a	g	0.29	0.131	0.037	3.51E-04	-
rs9398171	t	c	0.71	-0.130	0.037	4.02E-04	+
**rs17425951**	**a**	**g**	**0.12**	**-0.135**	**0.052**	**9.07E-03**	**+**
rs4340803	a	g	0.88	0.136	0.052	9.11E-03	-
rs17007509	t	c	0.88	0.135	0.052	9.13E-03	-
rs17007499	a	c	0.12	-0.134	0.052	1.02E-02	+
rs17425479	c	g	0.88	0.135	0.053	1.05E-02	-
rs10518305	t	g	0.88	0.133	0.052	1.06E-02	+
rs17007484	a	c	0.88	0.133	0.053	1.13E-02	-
**rs10243669**	**t**	**c**	**0.84**	**-0.113**	**0.046**	**1.43E-02**	+
rs12671484	a	g	0.84	-0.107	0.046	2.02E-02	+
rs12671457	a	c	0.84	-0.106	0.046	2.16E-02	+
rs1543270	t	c	0.47	-0.088	0.038	2.16E-02	+
rs12673689	a	g	0.14	0.106	0.049	3.02E-02	-
**rs7795536**	**a**	**g**	**0.17**	**-0.101**	**0.047**	**3.08E-02**	+
rs9658239	a	g	0.86	-0.103	0.048	3.32E-02	+
rs10249499	a	g	0.86	-0.103	0.049	3.49E-02	+
**rs17587111**	**a**	**g**	**0.81**	**0.104**	**0.049**	**3.52E-02**	**-**
rs1874479	a	g	0.86	-0.101	0.048	3.65E-02	+
rs12702233	t	c	0.68	0.076	0.036	3.71E-02	+
rs12535896	c	g	0.32	-0.076	0.036	3.74E-02	-
rs10231774	c	g	0.14	0.102	0.049	3.83E-02	-
rs12540816	a	c	0.68	0.074	0.036	4.23E-02	+
rs6964144	a	g	0.32	-0.073	0.037	4.44E-02	-
rs1722135	a	c	0.82	0.080	0.045	7.49E-02	-
**rs12325217**	**a**	**t**	**0.74**	**-0.072**	**0.041**	**7.53E-02**	**+**
rs1722134	a	t	0.17	-0.078	0.045	8.21E-02	+
rs1525829	t	c	0.17	-0.077	0.045	8.55E-02	+
rs809297	a	g	0.83	0.075	0.044	9.09E-02	-
rs10233070	t	c	0.06	0.129	0.077	9.56E-02	-
rs1722146	a	g	0.83	0.074	0.045	1.01E-01	-

rs1722147	a	g	0.83	0.074	0.045	1.01E-01	-
rs2464926	t	g	0.83	0.074	0.045	1.04E-01	-
rs977268	a	g	0.17	0.070	0.044	1.13E-01	+
rs10278715	t	g	0.67	0.057	0.036	1.14E-01	+
rs17172643	t	c	0.67	0.056	0.036	1.17E-01	+
rs11973257	a	g	0.83	-0.069	0.044	1.18E-01	-
rs10266929	a	g	0.33	-0.056	0.036	1.20E-01	-
rs1905992	a	g	0.67	0.055	0.036	1.23E-01	+
rs6463333	a	t	0.85	-0.072	0.049	1.40E-01	-
rs2949308	a	c	0.39	-0.057	0.039	1.43E-01	+
rs788773	a	g	0.77	-0.060	0.041	1.44E-01	+
rs1496499	t	g	0.54	0.049	0.033	1.45E-01	-
rs788771	t	c	0.77	-0.060	0.041	1.46E-01	+
rs2464927	t	c	0.23	0.059	0.041	1.47E-01	-
rs1722144	t	c	0.77	-0.059	0.041	1.48E-01	+
rs337286	t	c	0.15	0.071	0.049	1.48E-01	-
rs1852210	a	g	0.46	-0.048	0.034	1.57E-01	+
rs1657129	a	g	0.16	0.074	0.052	1.58E-01	-
rs788783	t	c	0.26	0.056	0.040	1.58E-01	-
rs2949838	t	c	0.54	0.047	0.034	1.61E-01	-
rs1542818	t	g	0.46	-0.047	0.033	1.63E-01	+
rs2965074	t	g	0.46	-0.047	0.033	1.64E-01	+
rs2965072	t	g	0.46	-0.047	0.033	1.64E-01	+
rs788728	a	g	0.22	0.061	0.044	1.64E-01	-
rs12538548	a	g	0.74	-0.055	0.040	1.64E-01	+
rs788727	t	c	0.46	-0.048	0.035	1.66E-01	+
rs788744	t	c	0.76	-0.056	0.040	1.66E-01	+
rs924140	t	c	0.46	-0.046	0.034	1.66E-01	+
rs17578201	a	g	0.23	0.056	0.041	1.69E-01	-
rs788735	a	g	0.23	0.055	0.040	1.70E-01	-
rs2690723	a	c	0.76	-0.056	0.041	1.70E-01	+
rs1722124	t	g	0.23	0.055	0.040	1.72E-01	-
**rs12155236**	**t**	**c**	**0.08**	**-0.102**	**0.075**	**1.73E-01**	**-**
rs2270628	t	c	0.21	0.056	0.041	1.73E-01	-
rs9806826	t	c	0.29	0.050	0.037	1.74E-01	-
**rs943318**	**a**	**g**	**0.67**	**-0.050**	**0.037**	**1.77E-01**	**+**
rs788734	a	c	0.23	0.053	0.040	1.82E-01	-
rs788732	t	c	0.77	-0.053	0.040	1.83E-01	+
rs1722139	a	c	0.76	-0.053	0.040	1.83E-01	+
rs788745	t	g	0.76	-0.053	0.040	1.83E-01	+
rs788733	c	g	0.23	0.053	0.040	1.83E-01	-
rs788738	t	c	0.77	-0.053	0.040	1.85E-01	+

rs7781417	a	c	0.84	-0.061	0.046	1.85E-01	-
rs2949837	a	t	0.67	0.050	0.038	1.86E-01	-
rs2462326	a	g	0.77	-0.053	0.040	1.86E-01	+
rs2462688	t	c	0.46	-0.046	0.035	1.86E-01	+
rs788743	a	g	0.77	-0.053	0.040	1.87E-01	+
rs700744	a	g	0.23	0.053	0.040	1.87E-01	-
rs337284	a	c	0.83	-0.063	0.048	1.87E-01	+
rs788736	t	c	0.23	0.053	0.040	1.88E-01	-
rs1722122	a	g	0.23	0.053	0.040	1.88E-01	-
rs13235531	a	g	0.84	-0.060	0.046	1.88E-01	-
rs1723917	c	g	0.23	0.053	0.040	1.89E-01	-
rs11979093	a	g	0.16	0.061	0.046	1.89E-01	+
rs788740	a	g	0.23	0.053	0.040	1.90E-01	-
rs6955810	t	g	0.82	-0.058	0.044	1.91E-01	-
rs2949307	t	c	0.62	-0.046	0.035	1.91E-01	+
rs4255045	t	c	0.24	0.061	0.047	1.92E-01	-
rs6463330	a	t	0.16	0.059	0.045	1.92E-01	+
rs943319	a	g	0.32	0.049	0.037	1.94E-01	-
rs7777076	a	c	0.16	0.059	0.045	1.95E-01	+
rs1859520	c	g	0.84	-0.059	0.045	1.96E-01	-
rs956010	a	g	0.86	0.064	0.049	1.96E-01	-
rs7778638	a	g	0.84	-0.059	0.046	1.97E-01	-
rs7793304	a	g	0.16	0.059	0.045	1.97E-01	+
rs7788742	a	g	0.84	-0.059	0.045	1.97E-01	-
rs7781943	t	c	0.16	0.059	0.045	1.98E-01	+
rs12112487	a	g	0.84	-0.058	0.045	1.98E-01	-
rs7806883	a	t	0.16	0.058	0.045	1.98E-01	+
rs12113686	t	g	0.16	0.058	0.045	1.99E-01	+
rs7783846	a	g	0.16	0.058	0.045	1.99E-01	+
rs7808435	a	t	0.84	-0.058	0.045	2.00E-01	-
rs3890267	a	g	0.84	-0.058	0.045	2.02E-01	-
rs1657139	t	c	0.15	0.062	0.049	2.04E-01	-
rs10275903	t	g	0.91	-0.076	0.061	2.07E-01	+
rs1722141	a	g	0.22	0.053	0.042	2.10E-01	-
rs2960427	a	g	0.24	0.050	0.040	2.16E-01	-
rs1535343	t	c	0.85	-0.060	0.048	2.18E-01	+
rs2091148	a	g	0.36	-0.042	0.035	2.25E-01	+
rs1657137	t	c	0.15	0.058	0.049	2.28E-01	-
rs3110697	a	g	0.42	0.041	0.034	2.28E-01	-
rs2492883	a	g	0.29	0.045	0.037	2.29E-01	-
rs11977526	a	g	0.41	-0.043	0.036	2.30E-01	+
rs388928	c	g	0.71	-0.045	0.038	2.34E-01	+

rs2437744	t	c	0.71	-0.045	0.037	2.34E-01	+
rs2949833	t	c	0.32	-0.045	0.038	2.37E-01	+
rs2906912	a	g	0.15	0.058	0.049	2.38E-01	-
rs2204488	c	g	0.37	-0.042	0.036	2.40E-01	+
rs788723	t	c	0.25	0.046	0.039	2.43E-01	-
rs3185708	a	g	0.16	-0.066	0.057	2.44E-01	+
rs2982432	t	c	0.15	0.056	0.048	2.46E-01	-
rs788724	t	c	0.25	0.045	0.039	2.46E-01	-
rs2437745	c	g	0.71	-0.043	0.037	2.47E-01	+
rs173164	a	g	0.85	-0.055	0.048	2.49E-01	+
rs404772	c	g	0.85	-0.062	0.054	2.53E-01	+
rs1404161	a	c	0.46	-0.039	0.034	2.54E-01	+
rs788776	t	g	0.46	-0.039	0.034	2.54E-01	+
rs1722115	c	g	0.81	-0.055	0.048	2.55E-01	+
rs2949306	t	c	0.46	-0.039	0.034	2.58E-01	+
rs2960426	c	g	0.64	0.039	0.034	2.59E-01	-
rs2917519	a	c	0.16	0.053	0.047	2.59E-01	-
rs2982433	a	g	0.85	-0.054	0.048	2.61E-01	+
rs788729	t	c	0.75	-0.044	0.039	2.62E-01	+
rs1657133	a	g	0.84	-0.053	0.047	2.63E-01	+
rs10260816	c	g	0.56	0.039	0.035	2.63E-01	-
rs788760	a	g	0.75	-0.046	0.041	2.63E-01	+
rs1525828	a	t	0.31	0.040	0.036	2.66E-01	-
rs12596510	a	g	0.16	0.052	0.047	2.68E-01	-
rs788764	a	g	0.75	-0.045	0.041	2.69E-01	+
rs2982447	t	c	0.84	-0.052	0.047	2.70E-01	+
rs788713	t	c	0.24	0.046	0.042	2.71E-01	-
rs10499638	a	c	0.76	-0.045	0.041	2.72E-01	+
rs12532079	t	g	0.24	0.045	0.041	2.73E-01	-
rs12599337	t	c	0.16	0.051	0.047	2.73E-01	-
rs344363	t	c	0.15	0.056	0.051	2.73E-01	-
rs1742462	a	c	0.84	-0.051	0.047	2.75E-01	+
rs6956126	t	g	0.15	-0.051	0.047	2.78E-01	+
rs1085924	t	c	0.24	0.044	0.041	2.79E-01	-
rs788761	c	g	0.46	-0.037	0.034	2.81E-01	+
rs1742464	t	c	0.16	0.050	0.047	2.84E-01	-
rs433268	c	g	0.16	0.058	0.054	2.85E-01	-
rs911392	t	c	0.84	-0.050	0.047	2.85E-01	+
rs1496497	t	g	0.81	-0.046	0.044	2.87E-01	+
rs911391	a	c	0.16	0.050	0.047	2.87E-01	-
rs1581571	t	c	0.47	-0.035	0.034	2.91E-01	+
rs7781438	t	c	0.52	0.036	0.034	2.92E-01	-

rs1917615	a	c	0.22	-0.046	0.043	2.93E-01	-
rs427732	c	g	0.16	0.057	0.054	2.93E-01	-
rs788725	t	c	0.48	-0.035	0.033	2.94E-01	+
rs788778	a	g	0.48	-0.036	0.034	2.94E-01	+
rs2982446	t	c	0.16	0.049	0.047	2.95E-01	-
rs2965083	t	c	0.36	-0.036	0.034	2.96E-01	+
rs2462687	t	c	0.31	0.043	0.042	2.99E-01	-
rs12325282	t	g	0.83	-0.049	0.047	3.05E-01	+
rs10255707	t	c	0.21	0.049	0.048	3.09E-01	-
rs2464923	a	g	0.39	0.036	0.036	3.09E-01	-
rs1496495	t	c	0.81	-0.043	0.043	3.14E-01	+
rs788762	a	g	0.26	0.041	0.041	3.14E-01	-
rs1525816	a	t	0.86	-0.055	0.055	3.15E-01	+
rs3848346	a	c	0.15	0.047	0.047	3.19E-01	-
rs11971230	a	g	0.92	-0.063	0.063	3.19E-01	-
rs10258520	t	c	0.98	0.133	0.134	3.20E-01	+
rs2965071	a	t	0.68	-0.036	0.036	3.21E-01	+
rs3848348	t	g	0.85	-0.047	0.047	3.22E-01	+
rs788714	t	c	0.52	0.034	0.035	3.22E-01	-
rs911396	t	g	0.26	0.065	0.066	3.22E-01	-
rs2982235	a	g	0.85	-0.047	0.048	3.23E-01	+
rs3813760	t	c	0.16	0.047	0.047	3.23E-01	-
rs1722108	t	c	0.39	0.035	0.036	3.23E-01	-
rs1742421	a	g	0.15	0.053	0.053	3.23E-01	-
rs11643835	a	g	0.84	-0.046	0.047	3.24E-01	+
rs2492881	a	g	0.85	-0.048	0.049	3.25E-01	+
rs9658238	a	g	0.81	-0.042	0.043	3.25E-01	+
rs17808002	a	g	0.06	0.076	0.077	3.25E-01	+
rs9806787	a	c	0.15	0.046	0.047	3.26E-01	-
rs12325141	a	g	0.85	-0.046	0.047	3.26E-01	+
rs9652777	c	g	0.84	-0.046	0.047	3.27E-01	+
rs9652786	t	c	0.84	-0.046	0.047	3.28E-01	+
rs17234242	t	c	0.06	0.076	0.077	3.28E-01	+
rs2974856	a	g	0.85	-0.047	0.048	3.28E-01	+
rs977269	a	g	0.94	-0.076	0.077	3.28E-01	-
rs2471557	c	g	0.32	0.035	0.036	3.29E-01	-
rs10278486	t	c	0.22	0.040	0.041	3.30E-01	+
rs11980950	t	c	0.08	0.063	0.064	3.31E-01	+
rs809298	a	c	0.32	0.035	0.036	3.33E-01	-
rs1657095	a	g	0.15	0.046	0.048	3.33E-01	-
rs2917523	t	c	0.15	0.046	0.048	3.33E-01	-
rs11766515	a	g	0.85	-0.048	0.050	3.35E-01	+

rs1521469	a	g	0.15	-0.049	0.051	3.37E-01	+
rs17234277	a	g	0.94	-0.075	0.078	3.37E-01	-
rs6945214	a	g	0.06	0.074	0.078	3.38E-01	+
rs788752	a	g	0.15	0.048	0.050	3.38E-01	-
rs992768	t	c	0.92	-0.062	0.065	3.39E-01	-
rs11981723	t	g	0.94	-0.074	0.078	3.39E-01	-
rs788765	c	g	0.68	-0.034	0.036	3.40E-01	+
rs1525831	t	c	0.68	-0.035	0.036	3.41E-01	+
rs11248898	a	g	0.16	0.045	0.047	3.42E-01	-
rs1657094	t	c	0.15	0.045	0.048	3.43E-01	-
rs2462685	a	g	0.79	-0.039	0.041	3.44E-01	+
rs1525819	c	g	0.21	0.039	0.041	3.45E-01	-
rs2965073	t	g	0.78	-0.038	0.041	3.46E-01	+
rs447782	t	c	0.85	-0.045	0.048	3.47E-01	+
rs344361	t	c	0.15	0.050	0.054	3.48E-01	-
rs1722148	a	c	0.21	0.039	0.041	3.48E-01	-
rs788751	a	g	0.15	0.046	0.050	3.52E-01	-
rs788718	a	g	0.19	0.040	0.043	3.53E-01	-
rs408286	a	c	0.15	0.048	0.052	3.53E-01	-
rs453494	t	c	0.85	-0.044	0.048	3.55E-01	+
rs788768	a	g	0.79	-0.038	0.041	3.57E-01	+
rs2492886	t	g	0.85	-0.044	0.047	3.59E-01	+
rs6969255	a	g	0.61	-0.033	0.036	3.59E-01	+
rs788766	a	g	0.79	-0.038	0.041	3.60E-01	+
rs11640914	a	g	0.15	0.044	0.048	3.61E-01	-
rs3743853	a	g	0.15	0.043	0.048	3.61E-01	-
rs11640407	a	t	0.85	-0.043	0.047	3.62E-01	+
rs378201	c	g	0.85	-0.044	0.048	3.63E-01	+
rs2189922	t	c	0.06	0.071	0.079	3.63E-01	+
rs6973777	a	g	0.06	0.071	0.078	3.65E-01	+
rs404413	t	c	0.16	0.044	0.048	3.67E-01	-
rs11642885	t	c	0.85	-0.043	0.047	3.68E-01	+
rs788749	a	t	0.86	-0.046	0.051	3.69E-01	+
rs9806945	a	g	0.85	-0.042	0.047	3.69E-01	+
rs2575359	a	t	0.85	-0.042	0.047	3.72E-01	+
rs17559	a	g	0.10	-0.051	0.058	3.73E-01	+
rs1657120	t	c	0.86	-0.045	0.050	3.73E-01	+
rs1534151	a	g	0.68	-0.032	0.036	3.74E-01	+
rs3817899	c	g	0.10	-0.051	0.058	3.75E-01	+
rs788716	a	g	0.81	-0.038	0.043	3.76E-01	+
rs940892	t	c	0.09	0.060	0.067	3.76E-01	-
rs17172644	t	c	0.20	-0.037	0.043	3.80E-01	-

rs2881018	t	c	0.06	0.069	0.078	3.80E-01	+
rs17233934	a	g	0.06	0.068	0.078	3.82E-01	+
rs1657116	a	g	0.85	-0.041	0.047	3.84E-01	+
rs6948362	t	c	0.04	0.077	0.089	3.87E-01	+
rs856572	t	c	0.85	0.042	0.049	3.89E-01	-
rs12325218	t	g	0.85	-0.041	0.048	3.90E-01	+
rs1625279	a	g	0.85	-0.041	0.047	3.90E-01	+
rs1742419	t	c	0.85	-0.041	0.047	3.90E-01	+
**rs1625393**	**a**	**g**	**0.15**	**0.041**	**0.047**	**3.92E-01**	**-**
rs12512052	a	g	0.32	0.037	0.043	3.92E-01	+
rs2960438	t	c	0.19	0.037	0.043	3.93E-01	-
rs1534150	t	c	0.68	-0.031	0.036	3.94E-01	+
rs12673906	a	t	0.31	0.033	0.038	3.95E-01	-
rs2453836	a	g	0.81	-0.041	0.048	3.95E-01	+
rs8046750	c	g	0.15	0.047	0.055	3.97E-01	-
rs788731	a	g	0.48	-0.028	0.033	3.98E-01	+
rs1723939	t	c	0.48	-0.028	0.033	3.99E-01	+
rs449530	a	g	0.15	0.040	0.048	3.99E-01	-
rs788782	a	t	0.49	0.034	0.040	4.01E-01	-
rs7781391	c	g	0.84	-0.043	0.051	4.02E-01	-
rs856588	a	g	0.15	-0.041	0.049	4.02E-01	+
rs1486173	t	g	0.90	0.048	0.057	4.03E-01	+
rs1589896	a	g	0.49	-0.032	0.038	4.03E-01	+
rs1657122	t	c	0.85	-0.040	0.048	4.04E-01	+
rs1742432	t	c	0.85	-0.040	0.048	4.04E-01	+
rs1657121	a	g	0.15	0.040	0.048	4.05E-01	-
rs448374	a	g	0.15	0.040	0.048	4.05E-01	-
rs410465	a	t	0.15	0.040	0.048	4.05E-01	-
rs12112098	a	g	0.86	0.049	0.059	4.06E-01	-
rs2754187	a	c	0.85	-0.040	0.048	4.06E-01	+
rs411193	t	c	0.85	-0.040	0.048	4.06E-01	+
rs448961	a	g	0.15	0.040	0.048	4.06E-01	-
rs420681	a	g	0.85	-0.040	0.048	4.06E-01	+
rs428123	a	g	0.85	-0.040	0.048	4.06E-01	+
rs260347	t	c	0.96	-0.074	0.089	4.06E-01	-
rs1742431	a	c	0.15	0.039	0.048	4.07E-01	-
rs260348	a	g	0.96	-0.074	0.089	4.08E-01	-
rs2754186	a	g	0.85	-0.039	0.048	4.08E-01	+
rs856551	a	c	0.15	-0.040	0.049	4.09E-01	+
rs2471553	a	g	0.69	-0.030	0.036	4.11E-01	+
rs7199384	a	g	0.15	0.039	0.047	4.11E-01	-
rs6955849	a	c	0.27	-0.037	0.045	4.12E-01	-

rs903889	t	g	0.78	-0.033	0.040	4.12E-01	+
rs6820163	a	c	0.82	0.044	0.053	4.12E-01	+
rs2754189	a	c	0.15	0.039	0.048	4.12E-01	-
rs1617255	t	c	0.15	0.039	0.047	4.12E-01	-
rs1742429	t	c	0.85	-0.039	0.047	4.14E-01	+
rs1657117	t	c	0.15	0.039	0.047	4.15E-01	-
rs11643973	t	c	0.15	0.039	0.047	4.15E-01	-
rs11643972	t	g	0.15	0.039	0.047	4.15E-01	-
rs1742426	c	g	0.15	0.039	0.047	4.16E-01	-
rs1742423	c	g	0.85	-0.039	0.047	4.16E-01	+
rs1657118	t	c	0.85	-0.039	0.047	4.17E-01	+
rs2471552	t	c	0.78	-0.033	0.040	4.17E-01	+
rs9648082	t	c	0.16	0.040	0.049	4.18E-01	-
rs1742422	a	g	0.15	0.038	0.047	4.19E-01	-
rs788741	a	c	0.48	-0.027	0.033	4.19E-01	+
rs788739	t	c	0.48	-0.027	0.033	4.20E-01	+
rs1525832	a	g	0.54	0.031	0.039	4.20E-01	-
rs1178435	t	c	0.16	0.037	0.045	4.20E-01	-
rs2345016	t	g	0.79	-0.034	0.042	4.22E-01	-
rs1742428	a	g	0.15	0.038	0.047	4.23E-01	-
rs260346	t	c	0.96	-0.071	0.089	4.25E-01	-
rs2132572	t	c	0.22	0.032	0.040	4.25E-01	-
rs2132571	t	c	0.31	0.029	0.036	4.26E-01	-
rs11770622	t	c	0.16	0.036	0.046	4.30E-01	-
rs788715	t	c	0.52	0.030	0.039	4.33E-01	-
rs9648084	t	g	0.12	0.041	0.053	4.33E-01	-
rs2268670	t	c	0.15	0.037	0.047	4.33E-01	-
rs4720544	t	c	0.21	0.032	0.042	4.42E-01	+
rs2815304	a	g	0.15	0.055	0.072	4.43E-01	+
rs6944635	a	g	0.80	-0.033	0.042	4.43E-01	+
rs856539	c	g	0.79	0.036	0.046	4.43E-01	-
rs2965069	t	c	0.34	-0.027	0.035	4.43E-01	+
rs6970565	t	c	0.61	0.027	0.035	4.47E-01	+
rs6950179	t	c	0.39	-0.026	0.035	4.50E-01	-
rs6961418	a	g	0.20	-0.032	0.042	4.51E-01	-
rs1077750	t	c	0.61	0.026	0.035	4.53E-01	+
rs260361	a	g	0.20	0.032	0.042	4.53E-01	+
rs260368	a	c	0.79	-0.031	0.042	4.53E-01	-
rs2116643	c	g	0.36	0.026	0.035	4.55E-01	+
rs7794166	t	c	0.39	-0.026	0.035	4.55E-01	-
rs260365	a	g	0.21	0.031	0.042	4.56E-01	+
rs260363	t	c	0.20	0.031	0.042	4.59E-01	+

rs12537178	a	g	0.22	-0.031	0.042	4.62E-01	-
rs7788288	c	g	0.39	-0.026	0.035	4.63E-01	-
rs9655050	c	g	0.12	0.039	0.053	4.65E-01	-
rs260369	a	t	0.04	0.066	0.090	4.65E-01	+
rs788711	t	c	0.26	0.028	0.039	4.73E-01	-
rs11760757	a	g	0.97	0.080	0.112	4.78E-01	+
rs9993	a	g	0.83	0.032	0.045	4.81E-01	-
rs4689639	t	c	0.31	-0.025	0.036	4.84E-01	-
rs4689637	a	g	0.31	-0.025	0.036	4.85E-01	-
rs10020957	a	g	0.41	-0.024	0.034	4.86E-01	-
rs1004028	t	c	0.83	0.032	0.046	4.88E-01	-
rs788717	t	c	0.78	-0.029	0.041	4.89E-01	+
rs1178436	t	c	0.18	0.030	0.044	4.94E-01	-
rs11644716	t	c	0.95	0.059	0.086	4.95E-01	-
rs788754	t	g	0.24	0.027	0.040	4.99E-01	-
rs4689667	a	t	0.30	-0.027	0.040	5.01E-01	-
rs17172610	a	g	0.17	-0.030	0.046	5.05E-01	+
rs788748	a	g	0.50	0.022	0.033	5.06E-01	-
rs260356	t	c	0.96	-0.058	0.088	5.08E-01	-
rs17492975	t	c	0.17	0.035	0.053	5.09E-01	-
rs856567	c	g	0.57	0.022	0.034	5.12E-01	+
rs13244568	t	c	0.13	0.034	0.052	5.14E-01	-
rs13230270	t	c	0.61	0.022	0.035	5.21E-01	+
rs2461127	a	g	0.42	0.022	0.034	5.27E-01	+
rs2471271	a	g	0.41	0.022	0.035	5.30E-01	+
rs11767059	t	c	0.19	-0.027	0.044	5.32E-01	-
rs2935265	t	c	0.52	0.026	0.043	5.45E-01	+
rs2471267	t	c	0.42	0.021	0.034	5.46E-01	+
rs11767935	t	c	0.12	0.032	0.053	5.47E-01	-
rs10499642	t	c	0.18	0.026	0.043	5.49E-01	-
rs4724427	c	g	0.19	-0.026	0.044	5.49E-01	-
rs12597803	t	c	0.14	0.034	0.056	5.50E-01	-
rs7781162	c	g	0.13	0.031	0.052	5.51E-01	-
rs3735666	a	c	0.83	0.027	0.045	5.51E-01	-
rs7780838	t	g	0.13	0.031	0.052	5.52E-01	-
rs1521470	a	g	0.15	-0.034	0.058	5.57E-01	+
rs1486170	a	g	0.62	0.020	0.034	5.59E-01	+
rs11982719	c	g	0.16	-0.027	0.047	5.67E-01	+
rs1486171	a	t	0.62	0.020	0.035	5.67E-01	+
rs2471551	c	g	0.20	0.024	0.042	5.68E-01	-
rs1532663	t	c	0.65	0.026	0.045	5.70E-01	+
rs4689085	t	c	0.28	-0.022	0.038	5.72E-01	-

rs1464930	a	g	0.81	-0.024	0.043	5.73E-01	+
rs7808776	t	c	0.12	0.030	0.053	5.75E-01	-
rs7792950	a	g	0.61	0.019	0.035	5.77E-01	+
rs6944785	t	c	0.18	0.024	0.044	5.77E-01	-
rs7791503	t	c	0.27	0.022	0.039	5.80E-01	-
rs11978000	t	c	0.17	-0.026	0.047	5.80E-01	+
rs11772890	a	g	0.61	0.019	0.035	5.80E-01	+
rs12754	t	g	0.83	0.025	0.045	5.81E-01	-
rs951449	t	c	0.53	0.020	0.036	5.82E-01	+
rs10277696	a	g	0.12	0.030	0.055	5.83E-01	-
rs11764490	a	g	0.88	-0.028	0.051	5.86E-01	+
rs10253036	t	c	0.12	0.029	0.053	5.87E-01	-
rs1486167	a	t	0.88	0.030	0.055	5.88E-01	+
rs4999732	a	g	0.40	-0.028	0.051	5.88E-01	-
rs6463347	a	c	0.71	0.021	0.039	5.89E-01	-
rs260362	a	g	0.09	0.034	0.063	5.89E-01	+
rs2471554	t	g	0.81	-0.023	0.043	5.90E-01	+
rs260366	t	g	0.91	-0.034	0.063	5.91E-01	-
rs260367	a	c	0.09	0.034	0.063	5.94E-01	+
rs1872588	a	g	0.80	0.026	0.048	5.95E-01	+
rs1723957	t	c	0.76	-0.021	0.040	5.97E-01	+
rs7800989	t	c	0.13	0.027	0.052	6.01E-01	-
rs13240808	a	g	0.87	-0.027	0.052	6.04E-01	+
rs904978	a	g	0.62	-0.018	0.035	6.08E-01	-
rs17172765	c	g	0.21	-0.022	0.044	6.09E-01	-
rs10240695	a	c	0.63	-0.018	0.035	6.10E-01	-
rs856589	a	g	0.32	-0.018	0.036	6.11E-01	+
rs856540	a	g	0.37	-0.019	0.036	6.11E-01	-
rs4689088	a	g	0.61	0.017	0.034	6.14E-01	+
rs788720	t	c	0.36	0.018	0.036	6.17E-01	-
rs344357	c	g	0.16	0.023	0.047	6.21E-01	-
rs260359	a	c	0.06	0.036	0.072	6.23E-01	+
rs10256378	t	c	0.80	0.021	0.043	6.25E-01	+
rs1486162	a	g	0.87	0.025	0.051	6.26E-01	+
rs7789988	a	c	0.25	0.019	0.039	6.26E-01	-
rs4234797	a	g	0.61	0.017	0.034	6.26E-01	+
rs10255992	t	c	0.13	-0.025	0.052	6.29E-01	-
rs6967436	a	g	0.19	-0.025	0.052	6.30E-01	-
rs2575352	a	g	0.15	0.023	0.048	6.31E-01	-
**rs11248888**	**c**	**g**	**0.89**	**0.027**	**0.056**	**6.33E-01**	**-**
rs2280497	a	g	0.83	0.022	0.047	6.35E-01	-
rs1486163	c	g	0.87	0.025	0.052	6.36E-01	+

rs10267258	t	c	0.87	0.025	0.052	6.39E-01	+
rs4234798	t	g	0.39	-0.016	0.034	6.39E-01	-
rs11761115	a	g	0.13	0.024	0.052	6.43E-01	-
rs17172634	a	g	0.87	0.024	0.052	6.44E-01	+
rs17172602	t	c	0.17	-0.021	0.046	6.44E-01	+
rs11766906	a	g	0.77	-0.018	0.040	6.54E-01	+
rs11766636	a	g	0.13	-0.023	0.052	6.54E-01	-
rs788709	t	c	0.09	0.032	0.074	6.63E-01	-
rs6975330	a	c	0.63	0.015	0.035	6.67E-01	-
rs11767621	a	g	0.79	0.019	0.046	6.72E-01	+
rs11764339	a	g	0.23	0.017	0.040	6.73E-01	-
rs2453849	a	g	0.27	0.017	0.041	6.74E-01	-
rs1486161	a	g	0.87	0.021	0.051	6.77E-01	+
rs11760834	a	t	0.12	0.022	0.053	6.77E-01	-
rs344359	t	c	0.17	0.019	0.046	6.78E-01	-
rs2575351	c	g	0.83	-0.021	0.052	6.78E-01	+
rs2965077	a	g	0.55	0.014	0.034	6.79E-01	-
rs2294619	a	g	0.82	-0.018	0.044	6.79E-01	+
rs12920634	t	c	0.11	-0.023	0.056	6.84E-01	+
rs2140917	c	g	0.16	-0.020	0.049	6.84E-01	-
rs957755	t	g	0.19	-0.018	0.044	6.89E-01	-
rs700750	a	c	0.62	0.014	0.035	6.89E-01	+
rs7794677	t	c	0.76	-0.016	0.040	6.89E-01	+
rs12596094	t	g	0.84	-0.020	0.050	6.94E-01	+
rs12597098	t	c	0.16	0.020	0.050	6.95E-01	-
rs2008103	a	c	0.22	-0.016	0.041	6.97E-01	-
rs12501357	t	c	0.38	0.014	0.035	6.98E-01	+
rs1404160	t	g	0.76	-0.016	0.040	6.99E-01	+
rs10937793	t	c	0.36	0.014	0.036	6.99E-01	+
rs7661066	t	c	0.38	0.014	0.035	6.99E-01	+
rs7661077	t	c	0.38	0.013	0.035	7.01E-01	+
rs2960429	c	g	0.45	-0.013	0.034	7.05E-01	+
rs13247207	t	c	0.12	0.020	0.053	7.07E-01	-
rs11769597	t	c	0.81	0.016	0.044	7.08E-01	+
rs856560	t	c	0.38	-0.013	0.034	7.10E-01	-
rs856563	t	c	0.38	-0.013	0.034	7.11E-01	-
rs1065663	a	g	0.83	-0.017	0.046	7.15E-01	+
rs1178432	t	c	0.12	0.021	0.058	7.17E-01	-
rs4689651	c	g	0.21	-0.015	0.043	7.22E-01	-
rs2965082	a	c	0.46	-0.012	0.033	7.23E-01	+
rs1917611	a	c	0.27	-0.014	0.038	7.23E-01	+
rs4724487	a	g	0.73	0.013	0.038	7.24E-01	+

rs12533001	t	g	0.19	-0.016	0.046	7.26E-01	-
rs11860469	t	c	0.93	0.024	0.068	7.27E-01	-
rs12595860	t	c	0.14	0.021	0.061	7.28E-01	-
rs1525238	a	g	0.27	-0.013	0.038	7.29E-01	+
rs879965	t	c	0.54	0.011	0.033	7.32E-01	-
rs1065656	c	g	0.30	0.013	0.038	7.33E-01	-
rs10228265	a	g	0.69	0.012	0.036	7.34E-01	+
rs10254077	t	c	0.08	-0.021	0.064	7.39E-01	-
rs1875341	a	g	0.45	-0.011	0.034	7.40E-01	-
rs12535368	a	g	0.27	-0.013	0.038	7.40E-01	-
rs12152570	t	c	0.38	0.011	0.035	7.45E-01	+
rs1352090	c	g	0.62	0.011	0.035	7.46E-01	+
rs4724488	a	g	0.27	-0.012	0.038	7.51E-01	-
rs17259858	t	c	0.73	0.012	0.038	7.51E-01	+
rs11865665	a	g	0.93	0.021	0.068	7.54E-01	-
**rs1385939**	**t**	**g**	**0.23**	**-0.014**	**0.044**	**7.55E-01**	**+**
rs6852212	c	g	0.21	-0.014	0.044	7.55E-01	-
rs879966	t	g	0.60	0.010	0.034	7.60E-01	+
rs11859266	c	g	0.93	0.021	0.068	7.61E-01	-
rs2049397	a	g	0.13	-0.016	0.052	7.63E-01	-
rs10229581	t	c	0.23	0.013	0.042	7.64E-01	-
rs10085433	a	c	0.13	0.015	0.052	7.72E-01	-
rs10081205	t	c	0.15	-0.015	0.052	7.74E-01	-
rs1742458	c	g	0.93	0.019	0.068	7.76E-01	-
rs17172768	a	g	0.23	0.012	0.042	7.77E-01	-
rs856565	a	c	0.27	-0.011	0.038	7.77E-01	+
rs17172769	c	g	0.23	0.012	0.042	7.79E-01	-
rs856569	a	g	0.26	-0.011	0.039	7.83E-01	+
rs17172767	t	c	0.77	-0.011	0.042	7.89E-01	+
rs878024	t	c	0.27	-0.010	0.038	7.93E-01	-
rs1864858	a	g	0.32	-0.010	0.037	7.95E-01	+
rs11770444	t	c	0.85	-0.012	0.047	7.99E-01	+
rs856568	a	t	0.74	0.010	0.038	8.00E-01	-
rs2473466	a	g	0.60	0.009	0.035	8.03E-01	+
rs13246216	a	c	0.85	-0.012	0.048	8.08E-01	+
rs1742424	a	g	0.06	-0.017	0.068	8.08E-01	+
rs6944270	t	c	0.60	0.011	0.045	8.11E-01	+
rs12702194	a	g	0.13	-0.012	0.052	8.12E-01	-
rs12333578	a	g	0.15	0.011	0.047	8.13E-01	-
rs13232034	a	g	0.13	-0.012	0.053	8.15E-01	-
rs7782135	t	c	0.73	0.009	0.038	8.18E-01	+
rs7787941	t	c	0.85	-0.011	0.048	8.22E-01	+

rs10951846	a	t	0.26	0.010	0.044	8.22E-01	-
rs10807932	a	t	0.62	0.008	0.035	8.23E-01	+
rs7804883	a	g	0.85	-0.011	0.048	8.23E-01	+
rs13231830	c	g	0.86	0.012	0.052	8.25E-01	+
rs10263950	t	g	0.15	0.011	0.048	8.25E-01	-
rs12154951	c	g	0.86	0.011	0.052	8.27E-01	+
rs2575369	t	c	0.57	0.008	0.036	8.32E-01	+
rs856559	a	g	0.27	-0.008	0.038	8.39E-01	+
rs13232036	a	t	0.15	0.010	0.047	8.39E-01	-
rs10243028	a	c	0.85	-0.010	0.048	8.39E-01	+
rs4689671	a	g	0.58	0.009	0.043	8.40E-01	+
**rs7185434**	**a**	**t**	**0.04**	**-0.027**	**0.134**	**8.40E-01**	**+**
rs6946867	a	t	0.85	-0.010	0.049	8.41E-01	+
rs13244644	c	g	0.86	0.010	0.052	8.41E-01	+
rs2280496	c	g	0.79	0.008	0.041	8.41E-01	+
rs2222627	t	c	0.14	-0.010	0.052	8.44E-01	-
rs6959938	a	g	0.15	0.009	0.047	8.45E-01	-
rs10233512	t	g	0.79	0.009	0.045	8.47E-01	+
rs6942846	t	c	0.92	-0.013	0.067	8.47E-01	+
rs949941	a	g	0.77	-0.008	0.042	8.48E-01	+
rs10807931	a	c	0.85	-0.009	0.047	8.49E-01	+
rs10231485	t	c	0.14	-0.010	0.052	8.49E-01	-
rs1525827	a	g	0.15	0.009	0.047	8.50E-01	-
rs1525826	a	g	0.15	0.009	0.047	8.51E-01	-
rs17172757	a	c	0.08	-0.012	0.063	8.51E-01	-
rs6954637	a	c	0.14	-0.010	0.052	8.53E-01	-
rs2235648	a	c	0.60	0.007	0.035	8.54E-01	+
rs13244360	a	g	0.86	0.010	0.052	8.55E-01	+
rs10081257	t	c	0.86	0.010	0.052	8.55E-01	+
rs6975917	t	c	0.85	-0.008	0.047	8.57E-01	+
rs949795	a	g	0.86	0.009	0.052	8.58E-01	+
rs10081377	a	g	0.86	0.009	0.052	8.58E-01	+
rs12149777	a	g	0.60	0.006	0.035	8.58E-01	+
rs7776585	a	t	0.14	-0.009	0.052	8.59E-01	-
rs12702234	c	g	0.32	-0.006	0.036	8.59E-01	+
rs7792709	c	g	0.14	-0.009	0.052	8.60E-01	-
rs10245478	a	g	0.14	-0.009	0.052	8.60E-01	-
rs1818943	t	c	0.86	0.009	0.052	8.60E-01	+
rs10232458	t	c	0.87	0.009	0.051	8.60E-01	+
rs6951099	t	c	0.85	-0.009	0.054	8.62E-01	+
rs1030640	a	g	0.92	0.011	0.063	8.63E-01	+
rs7799370	a	g	0.14	-0.009	0.052	8.63E-01	-

rs700753	c	g	0.35	-0.006	0.035	8.63E-01	-
rs700752	c	g	0.35	-0.006	0.035	8.69E-01	-
rs1905993	t	c	0.09	-0.010	0.058	8.69E-01	-
rs12670400	t	c	0.18	0.007	0.046	8.74E-01	-
rs7783023	t	c	0.14	-0.008	0.052	8.77E-01	-
rs6945559	t	g	0.13	-0.008	0.051	8.78E-01	-
rs7809174	t	c	0.14	-0.008	0.052	8.79E-01	-
rs17503164	a	g	0.91	0.009	0.058	8.81E-01	+
rs10274544	a	g	0.86	0.008	0.052	8.81E-01	+
rs10263873	c	g	0.87	0.008	0.051	8.84E-01	+
rs856547	t	g	0.92	0.009	0.062	8.84E-01	+
rs6943602	t	g	0.87	-0.007	0.051	8.86E-01	+
rs1525242	a	c	0.92	0.009	0.063	8.86E-01	+
rs17581802	t	c	0.09	-0.008	0.059	8.88E-01	-
rs4493818	c	g	0.87	-0.007	0.051	8.89E-01	+
rs6952108	t	c	0.92	0.008	0.063	8.93E-01	+
rs17172753	t	c	0.08	-0.008	0.062	8.94E-01	-
rs11770996	a	g	0.92	0.008	0.062	8.96E-01	+
**rs1872589**	**a**	**g**	**0.09**	**-0.008**	**0.059**	**8.97E-01**	**-**
rs10262325	t	c	0.13	0.006	0.051	8.99E-01	-
rs10225606	t	c	0.15	0.006	0.049	9.02E-01	-
rs891867	a	g	0.27	-0.005	0.038	9.02E-01	-
rs1917609	t	c	0.27	-0.005	0.038	9.04E-01	-
rs12112847	t	c	0.68	0.004	0.036	9.05E-01	-
rs17172752	a	g	0.92	0.007	0.062	9.05E-01	+
rs17172754	c	g	0.92	0.007	0.062	9.12E-01	+
rs1554465	a	g	0.78	0.005	0.041	9.13E-01	+
rs260336	a	g	0.04	0.011	0.101	9.14E-01	+
rs17587048	t	c	0.18	-0.005	0.045	9.16E-01	-
rs856543	a	g	0.92	0.007	0.062	9.16E-01	+
rs13228392	a	g	0.08	0.007	0.062	9.16E-01	-
rs12155377	t	g	0.87	-0.005	0.050	9.28E-01	+
rs1486165	t	c	0.14	-0.005	0.058	9.28E-01	-
rs856549	t	c	0.26	0.003	0.039	9.31E-01	+
rs6979582	t	c	0.13	0.004	0.050	9.38E-01	-
rs10235181	a	t	0.05	-0.007	0.105	9.44E-01	-
rs17581179	t	g	0.91	0.004	0.059	9.49E-01	+
rs17581193	a	c	0.91	0.004	0.059	9.50E-01	+
rs2437732	t	g	0.58	0.002	0.036	9.50E-01	+
rs4689665	t	c	0.17	-0.002	0.046	9.60E-01	-
rs856575	a	t	0.50	0.002	0.040	9.63E-01	-
rs17580738	t	c	0.92	0.003	0.061	9.65E-01	+

rs10951832	a	t	0.20	-0.002	0.046	9.67E-01	-
rs9931988	c	g	0.06	-0.003	0.078	9.69E-01	+
rs3813761	t	c	0.94	0.003	0.078	9.70E-01	-
rs6968126	a	g	0.13	0.002	0.050	9.70E-01	-
rs13246409	a	g	0.87	0.002	0.051	9.71E-01	+
rs6955080	t	c	0.13	0.002	0.050	9.74E-01	-
rs10228324	t	c	0.87	-0.002	0.050	9.75E-01	+
rs856541	t	c	0.25	-0.001	0.039	9.76E-01	+
rs1385944	a	t	0.08	-0.002	0.061	9.77E-01	-
rs2745189	t	c	0.14	-0.001	0.051	9.81E-01	-
rs866488	t	c	0.75	0.001	0.039	9.81E-01	-
rs856548	a	g	0.75	0.001	0.039	9.85E-01	-
rs17502849	t	c	0.92	-0.001	0.061	9.87E-01	+
rs1385943	a	g	0.08	-0.001	0.061	9.87E-01	-
rs17502828	a	g	0.92	0.001	0.061	9.91E-01	+
rs2568696	t	g	0.25	0.000	0.039	9.94E-01	+
rs17503015	t	g	0.08	0.000	0.061	9.97E-01	-
rs17502974	a	c	0.08	0.000	0.061	9.98E-01	-
rs17502891	a	c	0.08	0.000	0.061	9.99E-01	-
rs17502946	a	c	0.08	0.000	0.061	9.99E-01	-
rs856554	t	c	0.75	0.000	0.039	1.00E+00	-
rs17581039	t	c	0.92	0.000	0.061	1.00E+00	+

"-": effect allele associated with lower IGFBP-3 levels.
"+": effect allele associated with higher IGFBP-3 levels.
Resuts for 13 independent SNPs defined based on LD (settings r2>0.01, 1 Mb distance) are indicated by bold text.

Supplementary Table 12: Enrichment of putative regulatory elements among IGF-1 and IGFBP-3 associated loci

SNP	Proximity to IGF1 or IGFBP3	Region	Gene	Enhancer histone marks (ENCODE)	Enhancer histone marks (RODEMAP)	Promoter histone marks (ROADMAP)	DNAse Hypersensitivity	TF Motif (Prediction)	TF ChIP	Regulome Score
rs978458	cis	Intronic	IGF1	no data	8 cell types	SK.MUS	7 cell types	GR, Gm397	NFκB	1b
rs700753	cis	Intergenic	TNS3 / IGFBP3	NHLF, Huvec	10 cell types	LIV.A	25 cell types	Gfi1 ,Hsf, Nkx2	no data	2b
rs11977526	cis	Intergenic	IGFBP3	no data	no data	no data	no data	AP2, ELF1, Ets, NRSF, Zic4	no data	no score
rs646776	trans	Intergenic	CELSR2	6 cell types	46 cell types	4 cell types	6 cell types	NRSF, PU.1, TR4	CTCF, POLR2A, REST, TAF7	1f
rs4234798	trans	Intronic	SORCS2	HSMM	14 cell types	no data	30 cell types	no data	CEBPB	4
rs2153960	trans	Intronic	FOXO3	GM12878, H1	27 cell types	IPS.20	4 cell types	GCNF, Mef2, Pax-4, RXRA	RXRA, KAP1	4
rs780093	trans	Intronic	GCKR	no data	LIV.A	no data	GM12892, Adult_CD4_Th0	INSM1	no data	5
rs934073	trans	Intergenic	ASXL2	no data	no data	no data	ProgFib	TFII-I, ZEB1	no data	5
rs1065656	trans	3'-UTR	NUBP2	HMEC, NHEK	8 cell types	no data	4 cell types	AP-4, BHLHE40, Foxa, GR, PTF1, p300	no data	5
rs509035	trans	Intronic	GHSR	no data	no data	PANC.I	T-47D	Crx, Hoxb13, NFκB	no data	5

References

Broer L, Buchman AS, Deelen J, Evans DS, Faul JD, Lunetta KL, Sebastiani P, Smith JA, Smith AV, Tanaka T, Yu L, Arnold AM, Aspelund T, Benjamin EJ, De Jager PL, Eirkisdottir G, Evans DA, Garcia ME, Hofman A, Kaplan RC, Kardia SL, Kiel DP, Oostra BA, Orwoll ES, Parimi N, Psaty BM, Rivadeneira F, Rotter JI, Seshadri S, Singleton A, Tiemeier H, Uitterlinden AG, Zhao W, Bandinelli S, Bennett DA, Ferrucci L, Gudnason V, Harris TB, Karasik D, Launer LJ, Perls TT, Slagboom PE, Tranah GJ, Weir DR, Newman AB, van Duijn CM, Murabito JM (2014). GWAS of Longevity in CHARGE Consortium Confirms APOE and FOXO3 Candidacy. *The journals of gerontology. Series A, Biological sciences and medical sciences*.

Consortium CAD, Deloukas P, Kanoni S, Willenborg C, Farrall M, Assimes TL, Thompson JR, Ingelsson E, Saleheen D, Erdmann J, Goldstein BA, Stirrups K, Konig IR, Cazier JB, Johansson A, Hall AS, Lee JY, Willer CJ, Chambers JC, Esko T, Folkersen L, Goel A, Grundberg E, Havulinna AS, Ho WK, Hopewell JC, Eriksson N, Kleber ME, Kristiansson K, Lundmark P, Lyytikainen LP, Rafelt S, Shungin D, Strawbridge RJ, Thorleifsson G, Tikkanen E, Van Zuydam N, Voight BF, Waite LL, Zhang W, Ziegler A, Absher D, Altshuler D, Balmforth AJ, Barroso I, Braund PS, Burgdorf C, Claudi-Boehm S, Cox D, Dimitriou M, Do R, Consortium D, Consortium C, Doney AS, El Mokhtari N, Eriksson P, Fischer K, Fontanillas P, Franco-Cereceda A, Gigante B, Groop L, Gustafsson S, Hager J, Hallmans G, Han BG, Hunt SE, Kang HM, Illig T, Kessler T, Knowles JW, Kolovou G, Kuusisto J, Langenberg C, Langford C, Leander K, Lokki ML, Lundmark A, McCarthy MI, Meisinger C, Melander O, Mihailov E, Maouche S, Morris AD, Muller-Nurasyid M, Mu TC, Nikus K, Peden JF, Rayner NW, Rasheed A, Rosinger S, Rubin D, Rumpf MP, Schafer A, Sivananthan M, Song C, Stewart AF, Tan ST, Thorgeirsson G, van der Schoot CE, Wagner PJ, Wellcome Trust Case Control C, Wells GA, Wild PS, Yang TP, Amouyel P, Arveiler D, Basart H, Boehnke M, Boerwinkle E, Brambilla P, Cambien F, Cupples AL, de Faire U, Dehghan A, Diemert P, Epstein SE, Evans A, Ferrario MM, Ferrieres J, Gauguier D, Go AS, Goodall AH, Gudnason V, Hazen SL, Holm H, Iribarren C, Jang Y, Kahonen M, Kee F, Kim HS, Klopp N, Koenig W, Kratzer W, Kuulasmaa K, Laakso M, Laaksonen R, Lee JY, Lind L, Ouwehand WH, Parish S, Park JE, Pedersen NL, Peters A, Quertermous T, Rader DJ, Salomaa V, Schadt E, Shah SH, Sinisalo J, Stark K, Stefansson K, Tregouet DA, Virtamo J, Wallentin L, Wareham N, Zimmermann ME, Nieminen MS, Hengstenberg C, Sandhu MS, Pastinen T, Syvanen AC, Hovingh GK, Dedoussis G, Franks PW, Lehtimaki T, Metspalu A, Zalloua PA, Siegbahn A, Schreiber S, Ripatti S, Blankenberg SS, Perola M, Clarke R, Boehm BO, O'Donnell C, Reilly MP, Marz W, Collins R, Kathiresan S, Hamsten A, Kooner JS, Thorsteinsdottir U, Danesh J, Palmer CN, Roberts R, Watkins H, Schunkert H, Samani NJ (2013). Large-scale association analysis identifies new risk loci for coronary artery disease. *Nature genetics*. **45**, 25-33.

Coronary Artery Disease Genetics C (2011). A genome-wide association study in Europeans and South Asians identifies five new loci for coronary artery disease. *Nature genetics*. **43**, 339-344.

Dupuis J, Langenberg C, Prokopenko I, Saxena R, Soranzo N, Jackson AU, Wheeler E, Glazer NL, Bouatia-Naji N, Gloyn AL, Lindgren CM, Magi R, Morris AP, Randall J, Johnson T, Elliott P, Rybin D, Thorleifsson G, Steinthorsdottir V, Henneman P, Grallert H, Dehghan A, Hottenga JJ, Franklin CS, Navarro P, Song K, Goel A, Perry JR, Egan JM, Lajunen T, Grarup N, Sparso T, Doney A, Voight BF, Stringham HM, Li M, Kanoni S, Shrader P, Cavalcanti-Proenca C, Kumari M, Qi L, Timpson NJ, Gieger C, Zabena C, Rocheleau G, Ingelsson E, An P, O'Connell J, Luan J, Elliott A, McCarroll SA, Payne F, Roccasecca RM, Pattou F, Sethupathy P, Ardlie K, Ariyurek Y, Balkau B, Barter P, Beilby JP, Ben-Shlomo Y, Benediktsson R, Bennett AJ, Bergmann S, Bochud M, Boerwinkle E, Bonnefond A, Bonnycastle LL, Borch-Johnsen K, Bottcher Y, Brunner E, Bumpstead SJ, Charpentier G, Chen YD, Chines P, Clarke R, Coin LJ, Cooper MN, Cornelis M, Crawford G, Crisponi L, Day IN, de Geus EJ, Delplanque J, Dina C, Erdos MR, Fedson AC, Fischer-Rosinsky A, Forouhi NG, Fox CS, Frants R,

Franzosi MG, Galan P, Goodarzi MO, Graessler J, Groves CJ, Grundy S, Gwilliam R, Gyllensten U, Hadjadj S, Hallmans G, Hammond N, Han X, Hartikainen AL, Hassanali N, Hayward C, Heath SC, Hercberg S, Herder C, Hicks AA, Hillman DR, Hingorani AD, Hofman A, Hui J, Hung J, Isomaa B, Johnson PR, Jorgensen T, Jula A, Kaakinen M, Kaprio J, Kesaniemi YA, Kivimaki M, Knight B, Koskinen S, Kovacs P, Kyvik KO, Lathrop GM, Lawlor DA, Le Bacquer O, Lecoeur C, Li Y, Lyssenko V, Mahley R, Mangino M, Manning AK, Martinez-Larrad MT, McAteer JB, McCulloch LJ, McPherson R, Meisinger C, Melzer D, Meyre D, Mitchell BD, Morken MA, Mukherjee S, Naitza S, Narisu N, Neville MJ, Oostra BA, Orru M, Pakyz R, Palmer CN, Paolisso G, Pattaro C, Pearson D, Peden JF, Pedersen NL, Perola M, Pfeiffer AF, Pichler I, Polasek O, Posthuma D, Potter SC, Pouta A, Province MA, Psaty BM, Rathmann W, Rayner NW, Rice K, Ripatti S, Rivadeneira F, Roden M, Rolandsson O, Sandbaek A, Sandhu M, Sanna S, Sayer AA, Scheet P, Scott LJ, Seedorf U, Sharp SJ, Shields B, Sigurethsson G, Sijbrands EJ, Silveira A, Simpson L, Singleton A, Smith NL, Sovio U, Swift A, Syddall H, Syvanen AC, Tanaka T, Thorand B, Tichet J, Tonjes A, Tuomi T, Uitterlinden AG, van Dijk KW, van Hoek M, Varma D, Visvikis-Siest S, Vitart V, Vogelzangs N, Waeber G, Wagner PJ, Walley A, Walters GB, Ward KL, Watkins H, Weedon MN, Wild SH, Willemsen G, Witteman JC, Yarnell JW, Zeggini E, Zelenika D, Zethelius B, Zhai G, Zhao JH, Zillikens MC, Consortium D, Consortium G, Global BC, Borecki IB, Loos RJ, Meneton P, Magnusson PK, Nathan DM, Williams GH, Hattersley AT, Silander K, Salomaa V, Smith GD, Bornstein SR, Schwarz P, Spranger J, Karpe F, Shuldiner AR, Cooper C, Dedoussis GV, Serrano-Rios M, Morris AD, Lind L, Palmer LJ, Hu FB, Franks PW, Ebrahim S, Marmot M, Kao WH, Pankow JS, Sampson MJ, Kuusisto J, Laakso M, Hansen T, Pedersen O, Pramstaller PP, Wichmann HE, Illig T, Rudan I, Wright AF, Stumvoll M, Campbell H, Wilson JF, Anders Hamsten on behalf of Procardis C, investigators M, Bergman RN, Buchanan TA, Collins FS, Mohlke KL, Tuomilehto J, Valle TT, Altshuler D, Rotter JI, Siscovick DS, Penninx BW, Boomsma DI, Deloukas P, Spector TD, Frayling TM, Ferrucci L, Kong A, Thorsteinsdottir U, Stefansson K, van Duijn CM, Aulchenko YS, Cao A, Scuteri A, Schlessinger D, Uda M, Ruokonen A, Jarvelin MR, Waterworth DM, Vollenweider P, Peltonen L, Mooser V, Abecasis GR, Wareham NJ, Sladek R, Froguel P, Watanabe RM, Meigs JB, Groop L, Boehnke M, McCarthy MI, Florez JC, Barroso I (2010). New genetic loci implicated in fasting glucose homeostasis and their impact on type 2 diabetes risk. *Nature genetics*. **42**, 105-116.

Estrada K, Styrkarsdottir U, Evangelou E, Hsu YH, Duncan EL, Ntzani EE, Oei L, Albagha OM, Amin N, Kemp JP, Koller DL, Li G, Liu CT, Minster RL, Moayyeri A, Vandenput L, Willner D, Xiao SM, Yerges-Armstrong LM, Zheng HF, Alonso N, Eriksson J, Kammerer CM, Kaptoge SK, Leo PJ, Thorleifsson G, Wilson SG, Wilson JF, Aalto V, Alen M, Aragaki AK, Aspelund T, Center JR, Dailiana Z, Duggan DJ, Garcia M, Garcia-Giralt N, Giroux S, Hallmans G, Hocking LJ, Husted LB, Jameson KA, Khusainova R, Kim GS, Kooperberg C, Koromila T, Kruk M, Laaksonen M, Lacroix AZ, Lee SH, Leung PC, Lewis JR, Masi L, Mencej-Bedrac S, Nguyen TV, Nogues X, Patel MS, Prezelj J, Rose LM, Scollen S, Siggeirsdottir K, Smith AV, Svensson O, Trompet S, Trummer O, van Schoor NM, Woo J, Zhu K, Balcells S, Brandi ML, Buckley BM, Cheng S, Christiansen C, Cooper C, Dedoussis G, Ford I, Frost M, Goltzman D, Gonzalez-Macias J, Kahonen M, Karlsson M, Khusnutdinova E, Koh JM, Kollia P, Langdahl BL, Leslie WD, Lips P, Ljunggren O, Lorenc RS, Marc J, Mellstrom D, Obermayer-Pietsch B, Olmos JM, Pettersson-Kymmer U, Reid DM, Riancho JA, Ridker PM, Rousseau F, Slagboom PE, Tang NL, Urreizti R, Van Hul W, Viikari J, Zarrabeitia MT, Aulchenko YS, Castano-Betancourt M, Grundberg E, Herrera L, Ingvarsson T, Johannsdottir H, Kwan T, Li R, Luben R, Medina-Gomez C, Palsson ST, Reppe S, Rotter JI, Sigurdsson G, van Meurs JB, Verlaan D, Williams FM, Wood AR, Zhou Y, Gautvik KM, Pastinen T, Raychaudhuri S, Cauley JA, Chasman DI, Clark GR, Cummings SR, Danoy P, Dennison EM, Eastell R, Eisman JA, Gudnason V, Hofman A, Jackson RD, Jones G, Jukema JW, Khaw KT, Lehtimaki T, Liu Y, Lorentzon M, McCloskey E, Mitchell BD, Nandakumar K, Nicholson GC, Oostra BA, Peacock M, Pols HA, Prince RL, Raitakari O, Reid IR, Robbins J, Sambrook PN, Sham PC, Shuldiner AR, Tylavsky FA, van Duijn CM, Wareham NJ,

Cupples LA, Econs MJ, Evans DM, Harris TB, Kung AW, Psaty BM, Reeve J, Spector TD, Streeten EA, Zillikens MC, Thorsteinsdottir U, Ohlsson C, Karasik D, Richards JB, Brown MA, Stefansson K, Uitterlinden AG, Ralston SH, Ioannidis JP, Kiel DP, Rivadeneira F (2012). Genome-wide meta-analysis identifies 56 bone mineral density loci and reveals 14 loci associated with risk of fracture. *Nature genetics*. **44**, 491-501.

Fuchsberger C, Taliun D, Pramstaller PP, Pattaro C, consortium CK (2012). GWAtoolbox: an R package for fast quality control and handling of genome-wide association studies meta-analysis data. *Bioinformatics*. **28**, 444-445.

Grundberg E, Small KS, Hedman AK, Nica AC, Buil A, Keildson S, Bell JT, Yang TP, Meduri E, Barrett A, Nisbett J, Sekowska M, Wilk A, Shin SY, Glass D, Travers M, Min JL, Ring S, Ho K, Thorleifsson G, Kong A, Thorsteindottir U, Ainali C, Dimas AS, Hassanali N, Ingle C, Knowles D, Krestyaninova M, Lowe CE, Di Meglio P, Montgomery SB, Parts L, Potter S, Surdulescu G, Tsaprouni L, Tsoka S, Bataille V, Durbin R, Nestle FO, O'Rahilly S, Soranzo N, Lindgren CM, Zondervan KT, Ahmadi KR, Schadt EE, Stefansson K, Smith GD, McCarthy MI, Deloukas P, Dermitzakis ET, Spector TD, Multiple Tissue Human Expression Resource C (2012). Mapping cis- and trans-regulatory effects across multiple tissues in twins. *Nature genetics*. **44**, 1084-1089.

Heid IM, Jackson AU, Randall JC, Winkler TW, Qi L, Steinthorsdottir V, Thorleifsson G, Zillikens MC, Speliotes EK, Magi R, Workalemahu T, White CC, Bouatia-Naji N, Harris TB, Berndt SI, Ingelsson E, Willer CJ, Weedon MN, Luan J, Vedantam S, Esko T, Kilpelainen TO, Kutalik Z, Li S, Monda KL, Dixon AL, Holmes CC, Kaplan LM, Liang L, Min JL, Moffatt MF, Molony C, Nicholson G, Schadt EE, Zondervan KT, Feitosa MF, Ferreira T, Lango Allen H, Weyant RJ, Wheeler E, Wood AR, Magic, Estrada K, Goddard ME, Lettre G, Mangino M, Nyholt DR, Purcell S, Smith AV, Visscher PM, Yang J, McCarroll SA, Nemesh J, Voight BF, Absher D, Amin N, Aspelund T, Coin L, Glazer NL, Hayward C, Heard-Costa NL, Hottenga JJ, Johansson A, Johnson T, Kaakinen M, Kapur K, Ketkar S, Knowles JW, Kraft P, Kraja AT, Lamina C, Leitzmann MF, McKnight B, Morris AP, Ong KK, Perry JR, Peters MJ, Polasek O, Prokopenko I, Rayner NW, Ripatti S, Rivadeneira F, Robertson NR, Sanna S, Sovio U, Surakka I, Teumer A, van Wingerden S, Vitart V, Zhao JH, Cavalcanti-Proenca C, Chines PS, Fisher E, Kulzer JR, Lecoeur C, Narisu N, Sandholt C, Scott LJ, Silander K, Stark K, Tammesoo ML, Teslovich TM, Timpson NJ, Watanabe RM, Welch R, Chasman DI, Cooper MN, Jansson JO, Kettunen J, Lawrence RW, Pellikka N, Perola M, Vandenput L, Alavere H, Almgren P, Atwood LD, Bennett AJ, Biffar R, Bonnycastle LL, Bornstein SR, Buchanan TA, Campbell H, Day IN, Dei M, Dorr M, Elliott P, Erdos MR, Eriksson JG, Freimer NB, Fu M, Gaget S, Geus EJ, Gjesing AP, Grallert H, Grassler J, Groves CJ, Guiducci C, Hartikainen AL, Hassanali N, Havulinna AS, Herzig KH, Hicks AA, Hui J, Igl W, Jousilahti P, Jula A, Kajantie E, Kinnunen L, Kolcic I, Koskinen S, Kovacs P, Kroemer HK, Krzelj V, Kuusisto J, Kvaloy K, Laitinen J, Lantieri O, Lathrop GM, Lokki ML, Luben RN, Ludwig B, McArdle WL, McCarthy A, Morken MA, Nelis M, Neville MJ, Pare G, Parker AN, Peden JF, Pichler I, Pietilainen KH, Platou CG, Pouta A, Ridderstrale M, Samani NJ, Saramies J, Sinisalo J, Smit JH, Strawbridge RJ, Stringham HM, Swift AJ, Teder-Laving M, Thomson B, Usala G, van Meurs JB, van Ommen GJ, Vatin V, Volpato CB, Wallaschofski H, Walters GB, Widen E, Wild SH, Willemsen G, Witte DR, Zgaga L, Zitting P, Beilby JP, James AL, Kahonen M, Lehtimaki T, Nieminen MS, Ohlsson C, Palmer LJ, Raitakari O, Ridker PM, Stumvoll M, Tonjes A, Viikari J, Balkau B, Ben-Shlomo Y, Bergman RN, Boeing H, Smith GD, Ebrahim S, Froguel P, Hansen T, Hengstenberg C, Hveem K, Isomaa B, Jorgensen T, Karpe F, Khaw KT, Laakso M, Lawlor DA, Marre M, Meitinger T, Metspalu A, Midthjell K, Pedersen O, Salomaa V, Schwarz PE, Tuomi T, Tuomilehto J, Valle TT, Wareham NJ, Arnold AM, Beckmann JS, Bergmann S, Boerwinkle E, Boomsma DI, Caulfield MJ, Collins FS, Eiriksdottir G, Gudnason V, Gyllensten U, Hamsten A, Hattersley AT, Hofman A, Hu FB, Illig T, Iribarren C, Jarvelin MR, Kao WH, Kaprio J, Launer LJ, Munroe PB, Oostra B, Penninx BW, Pramstaller PP, Psaty BM, Quertermous T, Rissanen A, Rudan I, Shuldiner AR, Soranzo N, Spector TD, Syvanen AC, Uda M, Uitterlinden A, Volzke H,

Vollenweider P, Wilson JF, Witteman JC, Wright AF, Abecasis GR, Boehnke M, Borecki IB, Deloukas P, Frayling TM, Groop LC, Haritunians T, Hunter DJ, Kaplan RC, North KE, O'Connell JR, Peltonen L, Schlessinger D, Strachan DP, Hirschhorn JN, Assimes TL, Wichmann HE, Thorsteinsdottir U, van Duijn CM, Stefansson K, Cupples LA, Loos RJ, Barroso I, McCarthy MI, Fox CS, Mohlke KL, Lindgren CM (2010). Meta-analysis identifies 13 new loci associated with waist-hip ratio and reveals sexual dimorphism in the genetic basis of fat distribution. *Nature genetics*. **42**, 949-960.

Lango Allen H, Estrada K, Lettre G, Berndt SI, Weedon MN, Rivadeneira F, Willer CJ, Jackson AU, Vedantam S, Raychaudhuri S, Ferreira T, Wood AR, Weyant RJ, Segre AV, Speliotes EK, Wheeler E, Soranzo N, Park JH, Yang J, Gudbjartsson D, Heard-Costa NL, Randall JC, Qi L, Vernon Smith A, Magi R, Pastinen T, Liang L, Heid IM, Luan J, Thorleifsson G, Winkler TW, Goddard ME, Sin Lo K, Palmer C, Workalemahu T, Aulchenko YS, Johansson A, Zillikens MC, Feitosa MF, Esko T, Johnson T, Ketkar S, Kraft P, Mangino M, Prokopenko I, Absher D, Albrecht E, Ernst F, Glazer NL, Hayward C, Hottenga JJ, Jacobs KB, Knowles JW, Kutalik Z, Monda KL, Polasek O, Preuss M, Rayner NW, Robertson NR, Steinthorsdottir V, Tyrer JP, Voight BF, Wiklund F, Xu J, Zhao JH, Nyholt DR, Pellikka N, Perola M, Perry JR, Surakka I, Tammesoo ML, Altmaier EL, Amin N, Aspelund T, Bhangale T, Boucher G, Chasman DI, Chen C, Coin L, Cooper MN, Dixon AL, Gibson Q, Grundberg E, Hao K, Juhani Junttila M, Kaplan LM, Kettunen J, Konig IR, Kwan T, Lawrence RW, Levinson DF, Lorentzon M, McKnight B, Morris AP, Muller M, Suh Ngwa J, Purcell S, Rafelt S, Salem RM, Salvi E, Sanna S, Shi J, Sovio U, Thompson JR, Turchin MC, Vandenput L, Verlaan DJ, Vitart V, White CC, Ziegler A, Almgren P, Balmforth AJ, Campbell H, Citterio L, De Grandi A, Dominiczak A, Duan J, Elliott P, Elosua R, Eriksson JG, Freimer NB, Geus EJ, Glorioso N, Haiqing S, Hartikainen AL, Havulinna AS, Hicks AA, Hui J, Igl W, Illig T, Jula A, Kajantie E, Kilpelainen TO, Koiranen M, Kolcic I, Koskinen S, Kovacs P, Laitinen J, Liu J, Lokki ML, Marusic A, Maschio A, Meitinger T, Mulas A, Pare G, Parker AN, Peden JF, Petersmann A, Pichler I, Pietilainen KH, Pouta A, Ridderstrale M, Rotter JI, Sambrook JG, Sanders AR, Schmidt CO, Sinisalo J, Smit JH, Stringham HM, Bragi Walters G, Widen E, Wild SH, Willemsen G, Zagato L, Zgaga L, Zitting P, Alavere H, Farrall M, McArdle WL, Nelis M, Peters MJ, Ripatti S, van Meurs JB, Aben KK, Ardlie KG, Beckmann JS, Beilby JP, Bergman RN, Bergmann S, Collins FS, Cusi D, den Heijer M, Eiriksdottir G, Gejman PV, Hall AS, Hamsten A, Huikuri HV, Iribarren C, Kahonen M, Kaprio J, Kathiresan S, Kiemeney L, Kocher T, Launer LJ, Lehtimaki T, Melander O, Mosley TH, Jr., Musk AW, Nieminen MS, O'Donnell CJ, Ohlsson C, Oostra B, Palmer LJ, Raitakari O, Ridker PM, Rioux JD, Rissanen A, Rivolta C, Schunkert H, Shuldiner AR, Siscovick DS, Stumvoll M, Tonjes A, Tuomilehto J, van Ommen GJ, Viikari J, Heath AC, Martin NG, Montgomery GW, Province MA, Kayser M, Arnold AM, Atwood LD, Boerwinkle E, Chanock SJ, Deloukas P, Gieger C, Gronberg H, Hall P, Hattersley AT, Hengstenberg C, Hoffman W, Lathrop GM, Salomaa V, Schreiber S, Uda M, Waterworth D, Wright AF, Assimes TL, Barroso I, Hofman A, Mohlke KL, Boomsma DI, Caulfield MJ, Cupples LA, Erdmann J, Fox CS, Gudnason V, Gyllensten U, Harris TB, Hayes RB, Jarvelin MR, Mooser V, Munroe PB, Ouwehand WH, Penninx BW, Pramstaller PP, Quertermous T, Rudan I, Samani NJ, Spector TD, Volzke H, Watkins H, Wilson JF, Groop LC, Haritunians T, Hu FB, Kaplan RC, Metspalu A, North KE, Schlessinger D, Wareham NJ, Hunter DJ, O'Connell JR, Strachan DP, Wichmann HE, Borecki IB, van Duijn CM, Schadt EE, Thorsteinsdottir U, Peltonen L, Uitterlinden AG, Visscher PM, Chatterjee N, Loos RJ, Boehnke M, McCarthy MI, Ingelsson E, Lindgren CM, Abecasis GR, Stefansson K, Frayling TM, Hirschhorn JN (2010). Hundreds of variants clustered in genomic loci and biological pathways affect human height. *Nature*. **467**, 832-838.

Morris AP, Voight BF, Teslovich TM, Ferreira T, Segre AV, Steinthorsdottir V, Strawbridge RJ, Khan H, Grallert H, Mahajan A, Prokopenko I, Kang HM, Dina C, Esko T, Fraser RM, Kanoni S, Kumar A, Lagou V, Langenberg C, Luan J, Lindgren CM, Muller-Nurasyid M, Pechlivanis S, Rayner NW, Scott LJ, Wiltshire S, Yengo L, Kinnunen L, Rossin EJ, Raychaudhuri S, Johnson AD, Dimas AS, Loos RJ,

Vedantam S, Chen H, Florez JC, Fox C, Liu CT, Rybin D, Couper DJ, Kao WH, Li M, Cornelis MC, Kraft P, Sun Q, van Dam RM, Stringham HM, Chines PS, Fischer K, Fontanillas P, Holmen OL, Hunt SE, Jackson AU, Kong A, Lawrence R, Meyer J, Perry JR, Platou CG, Potter S, Rehnberg E, Robertson N, Sivapalaratnam S, Stancakova A, Stirrups K, Thorleifsson G, Tikkanen E, Wood AR, Almgren P, Atalay M, Benediktsson R, Bonnycastle LL, Burtt N, Carey J, Charpentier G, Crenshaw AT, Doney AS, Dorkhan M, Edkins S, Emilsson V, Eury E, Forsen T, Gertow K, Gigante B, Grant GB, Groves CJ, Guiducci C, Herder C, Hreidarsson AB, Hui J, James A, Jonsson A, Rathmann W, Klopp N, Kravic J, Krjutskov K, Langford C, Leander K, Lindholm E, Lobbens S, Mannisto S, Mirza G, Muhleisen TW, Musk B, Parkin M, Rallidis L, Saramies J, Sennblad B, Shah S, Sigurethsson G, Silveira A, Steinbach G, Thorand B, Trakalo J, Veglia F, Wennauer R, Winckler W, Zabaneh D, Campbell H, van Duijn C, Uitterlinden AG, Hofman A, Sijbrands E, Abecasis GR, Owen KR, Zeggini E, Trip MD, Forouhi NG, Syvanen AC, Eriksson JG, Peltonen L, Nothen MM, Balkau B, Palmer CN, Lyssenko V, Tuomi T, Isomaa B, Hunter DJ, Qi L, Wellcome Trust Case Control C, Meta-Analyses of G, Insulin-related traits Consortium I, Genetic Investigation of ATC, Asian Genetic Epidemiology Network-Type 2 Diabetes C, South Asian Type 2 Diabetes C, Shuldiner AR, Roden M, Barroso I, Wilsgaard T, Beilby J, Hovingh K, Price JF, Wilson JF, Rauramaa R, Lakka TA, Lind L, Dedoussis G, Njolstad I, Pedersen NL, Khaw KT, Wareham NJ, Keinanen-Kiukaanniemi SM, Saaristo TE, Korpi-Hyovalti E, Saltevo J, Laakso M, Kuusisto J, Metspalu A, Collins FS, Mohlke KL, Bergman RN, Tuomilehto J, Boehm BO, Gieger C, Hveem K, Cauchi S, Froguel P, Baldassarre D, Tremoli E, Humphries SE, Saleheen D, Danesh J, Ingelsson E, Ripatti S, Salomaa V, Erbel R, Jockel KH, Moebus S, Peters A, Illig T, de Faire U, Hamsten A, Morris AD, Donnelly PJ, Frayling TM, Hattersley AT, Boerwinkle E, Melander O, Kathiresan S, Nilsson PM, Deloukas P, Thorsteinsdottir U, Groop LC, Stefansson K, Hu F, Pankow JS, Dupuis J, Meigs JB, Altshuler D, Boehnke M, McCarthy MI, Replication DIG, Meta-analysis C (2012). Large-scale association analysis provides insights into the genetic architecture and pathophysiology of type 2 diabetes. *Nature genetics*. **44**, 981-990.

Saxena R, Hivert MF, Langenberg C, Tanaka T, Pankow JS, Vollenweider P, Lyssenko V, Bouatia-Naji N, Dupuis J, Jackson AU, Kao WH, Li M, Glazer NL, Manning AK, Luan J, Stringham HM, Prokopenko I, Johnson T, Grarup N, Boesgaard TW, Lecoeur C, Shrader P, O'Connell J, Ingelsson E, Couper DJ, Rice K, Song K, Andreasen CH, Dina C, Kottgen A, Le Bacquer O, Pattou F, Taneera J, Steinthorsdottir V, Rybin D, Ardlie K, Sampson M, Qi L, van Hoek M, Weedon MN, Aulchenko YS, Voight BF, Grallert H, Balkau B, Bergman RN, Bielinski SJ, Bonnefond A, Bonnycastle LL, Borch-Johnsen K, Bottcher Y, Brunner E, Buchanan TA, Bumpstead SJ, Cavalcanti-Proenca C, Charpentier G, Chen YD, Chines PS, Collins FS, Cornelis M, G JC, Delplanque J, Doney A, Egan JM, Erdos MR, Firmann M, Forouhi NG, Fox CS, Goodarzi MO, Graessler J, Hingorani A, Isomaa B, Jorgensen T, Kivimaki M, Kovacs P, Krohn K, Kumari M, Lauritzen T, Levy-Marchal C, Mayor V, McAteer JB, Meyre D, Mitchell BD, Mohlke KL, Morken MA, Narisu N, Palmer CN, Pakyz R, Pascoe L, Payne F, Pearson D, Rathmann W, Sandbaek A, Sayer AA, Scott LJ, Sharp SJ, Sijbrands E, Singleton A, Siscovick DS, Smith NL, Sparso T, Swift AJ, Syddall H, Thorleifsson G, Tonjes A, Tuomi T, Tuomilehto J, Valle TT, Waeber G, Walley A, Waterworth DM, Zeggini E, Zhao JH, consortium G, investigators M, Illig T, Wichmann HE, Wilson JF, van Duijn C, Hu FB, Morris AD, Frayling TM, Hattersley AT, Thorsteinsdottir U, Stefansson K, Nilsson P, Syvanen AC, Shuldiner AR, Walker M, Bornstein SR, Schwarz P, Williams GH, Nathan DM, Kuusisto J, Laakso M, Cooper C, Marmot M, Ferrucci L, Mooser V, Stumvoll M, Loos RJ, Altshuler D, Psaty BM, Rotter JI, Boerwinkle E, Hansen T, Pedersen O, Florez JC, McCarthy MI, Boehnke M, Barroso I, Sladek R, Froguel P, Meigs JB, Groop L, Wareham NJ, Watanabe RM (2010). Genetic variation in GIPR influences the glucose and insulin responses to an oral glucose challenge. *Nature genetics*. **42**, 142-148.

Schunkert H, Konig IR, Kathiresan S, Reilly MP, Assimes TL, Holm H, Preuss M, Stewart AF, Barbalic M, Gieger C, Absher D, Aherrahrou Z, Allayee H, Altshuler D, Anand SS, Andersen K, Anderson JL,

Ardissino D, Ball SG, Balmforth AJ, Barnes TA, Becker DM, Becker LC, Berger K, Bis JC, Boekholdt SM, Boerwinkle E, Braund PS, Brown MJ, Burnett MS, Buysschaert I, Cardiogenics, Carlquist JF, Chen L, Cichon S, Codd V, Davies RW, Dedoussis G, Dehghan A, Demissie S, Devaney JM, Diemert P, Do R, Doering A, Eifert S, Mokhtari NE, Ellis SG, Elosua R, Engert JC, Epstein SE, de Faire U, Fischer M, Folsom AR, Freyer J, Gigante B, Girelli D, Gretarsdottir S, Gudnason V, Gulcher JR, Halperin E, Hammond N, Hazen SL, Hofman A, Horne BD, Illig T, Iribarren C, Jones GT, Jukema JW, Kaiser MA, Kaplan LM, Kastelein JJ, Khaw KT, Knowles JW, Kolovou G, Kong A, Laaksonen R, Lambrechts D, Leander K, Lettre G, Li M, Lieb W, Loley C, Lotery AJ, Mannucci PM, Maouche S, Martinelli N, McKeown PP, Meisinger C, Meitinger T, Melander O, Merlini PA, Mooser V, Morgan T, Muhleisen TW, Muhlestein JB, Munzel T, Musunuru K, Nahrstaedt J, Nelson CP, Nothen MM, Olivieri O, Patel RS, Patterson CC, Peters A, Peyvandi F, Qu L, Quyyumi AA, Rader DJ, Rallidis LS, Rice C, Rosendaal FR, Rubin D, Salomaa V, Sampietro ML, Sandhu MS, Schadt E, Schafer A, Schillert A, Schreiber S, Schrezenmeir J, Schwartz SM, Siscovick DS, Sivananthan M, Sivapalaratnam S, Smith A, Smith TB, Snoep JD, Soranzo N, Spertus JA, Stark K, Stirrups K, Stoll M, Tang WH, Tennstedt S, Thorgeirsson G, Thorleifsson G, Tomaszewski M, Uitterlinden AG, van Rij AM, Voight BF, Wareham NJ, Wells GA, Wichmann HE, Wild PS, Willenborg C, Witteman JC, Wright BJ, Ye S, Zeller T, Ziegler A, Cambien F, Goodall AH, Cupples LA, Quertermous T, Marz W, Hengstenberg C, Blankenberg S, Ouwehand WH, Hall AS, Deloukas P, Thompson JR, Stefansson K, Roberts R, Thorsteinsdottir U, O'Donnell CJ, McPherson R, Erdmann J, Consortium CA, Samani NJ (2011). Large-scale association analysis identifies 13 new susceptibility loci for coronary artery disease. *Nature genetics*. **43**, 333-338.

Schurmann C, Heim K, Schillert A, Blankenberg S, Carstensen M, Dorr M, Endlich K, Felix SB, Gieger C, Grallert H, Herder C, Hoffmann W, Homuth G, Illig T, Kruppa J, Meitinger T, Muller C, Nauck M, Peters A, Rettig R, Roden M, Strauch K, Volker U, Volzke H, Wahl S, Wallaschofski H, Wild PS, Zeller T, Teumer A, Prokisch H, Ziegler A (2012). Analyzing illumina gene expression microarray data from different tissues: methodological aspects of data analysis in the metaxpress consortium. *PloS one*. **7**, e50938.

Shin SY, Fauman EB, Petersen AK, Krumsiek J, Santos R, Huang J, Arnold M, Erte I, Forgetta V, Yang TP, Walter K, Menni C, Chen L, Vasquez L, Valdes AM, Hyde CL, Wang V, Ziemek D, Roberts P, Xi L, Grundberg E, Multiple Tissue Human Expression Resource C, Waldenberger M, Richards JB, Mohney RP, Milburn MV, John SL, Trimmer J, Theis FJ, Overington JP, Suhre K, Brosnan MJ, Gieger C, Kastenmuller G, Spector TD, Soranzo N (2014). An atlas of genetic influences on human blood metabolites. *Nature genetics*. **46**, 543-550.

Soranzo N, Sanna S, Wheeler E, Gieger C, Radke D, Dupuis J, Bouatia-Naji N, Langenberg C, Prokopenko I, Stolerman E, Sandhu MS, Heeney MM, Devaney JM, Reilly MP, Ricketts SL, Stewart AF, Voight BF, Willenborg C, Wright B, Altshuler D, Arking D, Balkau B, Barnes D, Boerwinkle E, Bohm B, Bonnefond A, Bonnycastle LL, Boomsma DI, Bornstein SR, Bottcher Y, Bumpstead S, Burnett-Miller MS, Campbell H, Cao A, Chambers J, Clark R, Collins FS, Coresh J, de Geus EJ, Dei M, Deloukas P, Doring A, Egan JM, Elosua R, Ferrucci L, Forouhi N, Fox CS, Franklin C, Franzosi MG, Gallina S, Goel A, Graessler J, Grallert H, Greinacher A, Hadley D, Hall A, Hamsten A, Hayward C, Heath S, Herder C, Homuth G, Hottenga JJ, Hunter-Merrill R, Illig T, Jackson AU, Jula A, Kleber M, Knouff CW, Kong A, Kooner J, Kottgen A, Kovacs P, Krohn K, Kuhnel B, Kuusisto J, Laakso M, Lathrop M, Lecoeur C, Li M, Li M, Loos RJ, Luan J, Lyssenko V, Magi R, Magnusson PK, Malarstig A, Mangino M, Martinez-Larrad MT, Marz W, McArdle WL, McPherson R, Meisinger C, Meitinger T, Melander O, Mohlke KL, Mooser VE, Morken MA, Narisu N, Nathan DM, Nauck M, O'Donnell C, Oexle K, Olla N, Pankow JS, Payne F, Peden JF, Pedersen NL, Peltonen L, Perola M, Polasek O, Porcu E, Rader DJ, Rathmann W, Ripatti S, Rocheleau G, Roden M, Rudan I, Salomaa V, Saxena R, Schlessinger D, Schunkert H, Schwarz P, Seedorf U, Selvin E, Serrano-Rios M, Shrader P, Silveira A, Siscovick D, Song K, Spector TD, Stefansson K, Steinthorsdottir V, Strachan DP, Strawbridge R,

Stumvoll M, Surakka I, Swift AJ, Tanaka T, Teumer A, Thorleifsson G, Thorsteinsdottir U, Tonjes A, Usala G, Vitart V, Volzke H, Wallaschofski H, Waterworth DM, Watkins H, Wichmann HE, Wild SH, Willemsen G, Williams GH, Wilson JF, Winkelmann J, Wright AF, Wtccc, Zabena C, Zhao JH, Epstein SE, Erdmann J, Hakonarson HH, Kathiresan S, Khaw KT, Roberts R, Samani NJ, Fleming MD, Sladek R, Abecasis G, Boehnke M, Froguel P, Groop L, McCarthy MI, Kao WH, Florez JC, Uda M, Wareham NJ, Barroso I, Meigs JB (2010). Common variants at 10 genomic loci influence hemoglobin A(1)(C) levels via glycemic and nonglycemic pathways. *Diabetes*. **59**, 3229-3239.

Speliotes EK, Willer CJ, Berndt SI, Monda KL, Thorleifsson G, Jackson AU, Lango Allen H, Lindgren CM, Luan J, Magi R, Randall JC, Vedantam S, Winkler TW, Qi L, Workalemahu T, Heid IM, Steinthorsdottir V, Stringham HM, Weedon MN, Wheeler E, Wood AR, Ferreira T, Weyant RJ, Segre AV, Estrada K, Liang L, Nemesh J, Park JH, Gustafsson S, Kilpelainen TO, Yang J, Bouatia-Naji N, Esko T, Feitosa MF, Kutalik Z, Mangino M, Raychaudhuri S, Scherag A, Smith AV, Welch R, Zhao JH, Aben KK, Absher DM, Amin N, Dixon AL, Fisher E, Glazer NL, Goddard ME, Heard-Costa NL, Hoesel V, Hottenga JJ, Johansson A, Johnson T, Ketkar S, Lamina C, Li S, Moffatt MF, Myers RH, Narisu N, Perry JR, Peters MJ, Preuss M, Ripatti S, Rivadeneira F, Sandholt C, Scott LJ, Timpson NJ, Tyrer JP, van Wingerden S, Watanabe RM, White CC, Wiklund F, Barlassina C, Chasman DI, Cooper MN, Jansson JO, Lawrence RW, Pellikka N, Prokopenko I, Shi J, Thiering E, Alavere H, Alibrandi MT, Almgren P, Arnold AM, Aspelund T, Atwood LD, Balkau B, Balmforth AJ, Bennett AJ, Ben-Shlomo Y, Bergman RN, Bergmann S, Biebermann H, Blakemore AI, Boes T, Bonnycastle LL, Bornstein SR, Brown MJ, Buchanan TA, Busonero F, Campbell H, Cappuccio FP, Cavalcanti-Proenca C, Chen YD, Chen CM, Chines PS, Clarke R, Coin L, Connell J, Day IN, den Heijer M, Duan J, Ebrahim S, Elliott P, Elosua R, Eiriksdottir G, Erdos MR, Eriksson JG, Facheris MF, Felix SB, Fischer-Posovszky P, Folsom AR, Friedrich N, Freimer NB, Fu M, Gaget S, Gejman PV, Geus EJ, Gieger C, Gjesing AP, Goel A, Goyette P, Grallert H, Grassler J, Greenawalt DM, Groves CJ, Gudnason V, Guiducci C, Hartikainen AL, Hassanali N, Hall AS, Havulinna AS, Hayward C, Heath AC, Hengstenberg C, Hicks AA, Hinney A, Hofman A, Homuth G, Hui J, Igl W, Iribarren C, Isomaa B, Jacobs KB, Jarick I, Jewell E, John U, Jorgensen T, Jousilahti P, Jula A, Kaakinen M, Kajantie E, Kaplan LM, Kathiresan S, Kettunen J, Kinnunen L, Knowles JW, Kolcic I, Konig IR, Koskinen S, Kovacs P, Kuusisto J, Kraft P, Kvaloy K, Laitinen J, Lantieri O, Lanzani C, Launer LJ, Lecoeur C, Lehtimaki T, Lettre G, Liu J, Lokki ML, Lorentzon M, Luben RN, Ludwig B, Magic, Manunta P, Marek D, Marre M, Martin NG, McArdle WL, McCarthy A, McKnight B, Meitinger T, Melander O, Meyre D, Midthjell K, Montgomery GW, Morken MA, Morris AP, Mulic R, Ngwa JS, Nelis M, Neville MJ, Nyholt DR, O'Donnell CJ, O'Rahilly S, Ong KK, Oostra B, Pare G, Parker AN, Perola M, Pichler I, Pietilainen KH, Platou CG, Polasek O, Pouta A, Rafelt S, Raitakari O, Rayner NW, Ridderstrale M, Rief W, Ruokonen A, Robertson NR, Rzehak P, Salomaa V, Sanders AR, Sandhu MS, Sanna S, Saramies J, Savolainen MJ, Scherag S, Schipf S, Schreiber S, Schunkert H, Silander K, Sinisalo J, Siscovick DS, Smit JH, Soranzo N, Sovio U, Stephens J, Surakka I, Swift AJ, Tammesoo ML, Tardif JC, Teder-Laving M, Teslovich TM, Thompson JR, Thomson B, Tonjes A, Tuomi T, van Meurs JB, van Ommen GJ, Vatin V, Viikari J, Visvikis-Siest S, Vitart V, Vogel CI, Voight BF, Waite LL, Wallaschofski H, Walters GB, Widen E, Wiegand S, Wild SH, Willemsen G, Witte DR, Witteman JC, Xu J, Zhang Q, Zgaga L, Ziegler A, Zitting P, Beilby JP, Farooqi IS, Hebebrand J, Huikuri HV, James AL, Kahonen M, Levinson DF, Macciardi F, Nieminen MS, Ohlsson C, Palmer LJ, Ridker PM, Stumvoll M, Beckmann JS, Boeing H, Boerwinkle E, Boomsma DI, Caulfield MJ, Chanock SJ, Collins FS, Cupples LA, Smith GD, Erdmann J, Froguel P, Gronberg H, Gyllensten U, Hall P, Hansen T, Harris TB, Hattersley AT, Hayes RB, Heinrich J, Hu FB, Hveem K, Illig T, Jarvelin MR, Kaprio J, Karpe F, Khaw KT, Kiemeney LA, Krude H, Laakso M, Lawlor DA, Metspalu A, Munroe PB, Ouwehand WH, Pedersen O, Penninx BW, Peters A, Pramstaller PP, Quertermous T, Reinehr T, Rissanen A, Rudan I, Samani NJ, Schwarz PE, Shuldiner AR, Spector TD, Tuomilehto J, Uda M, Uitterlinden A, Valle TT, Wabitsch M, Waeber G, Wareham NJ, Watkins H, Procardis C, Wilson JF,

Wright AF, Zillikens MC, Chatterjee N, McCarroll SA, Purcell S, Schadt EE, Visscher PM, Assimes TL, Borecki IB, Deloukas P, Fox CS, Groop LC, Haritunians T, Hunter DJ, Kaplan RC, Mohlke KL, O'Connell JR, Peltonen L, Schlessinger D, Strachan DP, van Duijn CM, Wichmann HE, Frayling TM, Thorsteinsdottir U, Abecasis GR, Barroso I, Boehnke M, Stefansson K, North KE, McCarthy MI, Hirschhorn JN, Ingelsson E, Loos RJ (2010). Association analyses of 249,796 individuals reveal 18 new loci associated with body mass index. *Nature genetics*. **42**, 937-948.

Suhre K, Shin SY, Petersen AK, Mohney RP, Meredith D, Wagele B, Altmaier E, CardioGram, Deloukas P, Erdmann J, Grundberg E, Hammond CJ, de Angelis MH, Kastenmuller G, Kottgen A, Kronenberg F, Mangino M, Meisinger C, Meitinger T, Mewes HW, Milburn MV, Prehn C, Raffler J, Ried JS, Romisch-Margl W, Samani NJ, Small KS, Wichmann HE, Zhai G, Illig T, Spector TD, Adamski J, Soranzo N, Gieger C (2011). Human metabolic individuality in biomedical and pharmaceutical research. *Nature*. **477**, 54-60.

Voight BF, Scott LJ, Steinthorsdottir V, Morris AP, Dina C, Welch RP, Zeggini E, Huth C, Aulchenko YS, Thorleifsson G, McCulloch LJ, Ferreira T, Grallert H, Amin N, Wu G, Willer CJ, Raychaudhuri S, McCarroll SA, Langenberg C, Hofmann OM, Dupuis J, Qi L, Segre AV, van Hoek M, Navarro P, Ardlie K, Balkau B, Benediktsson R, Bennett AJ, Blagieva R, Boerwinkle E, Bonnycastle LL, Bengtsson Bostrom K, Bravenboer B, Bumpstead S, Burtt NP, Charpentier G, Chines PS, Cornelis M, Couper DJ, Crawford G, Doney AS, Elliott KS, Elliott AL, Erdos MR, Fox CS, Franklin CS, Ganser M, Gieger C, Grarup N, Green T, Griffin S, Groves CJ, Guiducci C, Hadjadj S, Hassanali N, Herder C, Isomaa B, Jackson AU, Johnson PR, Jorgensen T, Kao WH, Klopp N, Kong A, Kraft P, Kuusisto J, Lauritzen T, Li M, Lieverse A, Lindgren CM, Lyssenko V, Marre M, Meitinger T, Midthjell K, Morken MA, Narisu N, Nilsson P, Owen KR, Payne F, Perry JR, Petersen AK, Platou C, Proenca C, Prokopenko I, Rathmann W, Rayner NW, Robertson NR, Rocheleau G, Roden M, Sampson MJ, Saxena R, Shields BM, Shrader P, Sigurdsson G, Sparso T, Strassburger K, Stringham HM, Sun Q, Swift AJ, Thorand B, Tichet J, Tuomi T, van Dam RM, van Haeften TW, van Herpt T, van Vliet-Ostaptchouk JV, Walters GB, Weedon MN, Wijmenga C, Witteman J, Bergman RN, Cauchi S, Collins FS, Gloyn AL, Gyllensten U, Hansen T, Hide WA, Hitman GA, Hofman A, Hunter DJ, Hveem K, Laakso M, Mohlke KL, Morris AD, Palmer CN, Pramstaller PP, Rudan I, Sijbrands E, Stein LD, Tuomilehto J, Uitterlinden A, Walker M, Wareham NJ, Watanabe RM, Abecasis GR, Boehm BO, Campbell H, Daly MJ, Hattersley AT, Hu FB, Meigs JB, Pankow JS, Pedersen O, Wichmann HE, Barroso I, Florez JC, Frayling TM, Groop L, Sladek R, Thorsteinsdottir U, Wilson JF, Illig T, Froguel P, van Duijn CM, Stefansson K, Altshuler D, Boehnke M, McCarthy MI, investigators M, Consortium G (2010). Twelve type 2 diabetes susceptibility loci identified through large-scale association analysis. *Nature genetics*. **42**, 579-589.

Westra HJ, Peters MJ, Esko T, Yaghootkar H, Schurmann C, Kettunen J, Christiansen MW, Fairfax BP, Schramm K, Powell JE, Zhernakova A, Zhernakova DV, Veldink JH, Van den Berg LH, Karjalainen J, Withoff S, Uitterlinden AG, Hofman A, Rivadeneira F, t Hoen PA, Reinmaa E, Fischer K, Nelis M, Milani L, Melzer D, Ferrucci L, Singleton AB, Hernandez DG, Nalls MA, Homuth G, Nauck M, Radke D, Volker U, Perola M, Salomaa V, Brody J, Suchy-Dicey A, Gharib SA, Enquobahrie DA, Lumley T, Montgomery GW, Makino S, Prokisch H, Herder C, Roden M, Grallert H, Meitinger T, Strauch K, Li Y, Jansen RC, Visscher PM, Knight JC, Psaty BM, Ripatti S, Teumer A, Frayling TM, Metspalu A, van Meurs JB, Franke L (2013). Systematic identification of trans eQTLs as putative drivers of known disease associations. *Nature genetics*. **45**, 1238-1243.

Yang J, Lee SH, Goddard ME, Visscher PM (2011). GCTA: a tool for genome-wide complex trait analysis. *American journal of human genetics*. **88**, 76-82.
