# Supplementary material for: Genomewide meta‐analysis identifies loci associated with IGF‐I and IGFBP‐3 levels with impact on age‐related traits
Source: Aging Cell. 2016 Jun 21;15(5):811–24. doi: 10.1111/acel.12490 (PMC5013013; doi:10.1111/acel.12490)
Supplement: Supplementary file 2 — Data S1 Design and funding of participating cohort studies. [file ACEL-15-811-s002.doc]

**Genome-wide meta-analysis reveals loci associated with circulating levels of IGF-I and IGFBP-3 with implications for metabolic and age related traits**

**Study design and recruitment**

The Baltimore Longitudinal Study of Aging (BLSA):

The Baltimore Longitudinal Study of Aging (BLSA) is an ongoing longitudinal cohort study of community-dwelling healthy adult volunteers aged 21 years and older. The cohort was initiated in 1958 to examine physiological and psychological aspects of the aging process through biennial medical examinations, neuropsychological battery and physiological testing. Although the BLSA visits occur every 2 years, the sampling times were modified in 2003 due to nonlinear changes at the oldest ages and individuals aged 80 years and older are examined annually. At all visits, fasting blood samples are collected and stored at −80 °C from participants who were not allowed to smoke, take medications or engage in physical activity prior to blood draw .

The Cardiovascular Health Study (CHS):

CHS is a prospective population-based cohort study of CVD, mortality, and other outcomes in 65+ year old adults. Subjects were recruited at four Field Centers: Forsyth County, NC; Sacramento County, CA; Washington County, MD; and Pittsburgh, PA. Planning, coordination, and data archiving is conducted at the CHS Coordinating Center (University of Washington). The "original” CHS cohort of 5201 elderly adults was recruited in 1989-1990. In 1992-1993 a "new” CHS cohort of 687 additional participants was recruited, most of whom were African-American individuals, yielding a total of 5888. Those eligible to participate included persons identified from Medicare eligibility lists who were: 1) 65 or older; 2) non-institutionalized; 3) expected to remain in the area for 3 years; and 4) able to give informed consent. In CHS, health-related variables were collected annually in standardized fashion via examinations and questionnaires. Blood samples were drawn on all participants early in the examination after an 8-12 hour fast.

Of 5888 individuals enrolled in CHS, 3908 who were free of clinical cardiovascular disease at study baseline, had DNA available and provided appropriate consent were included in the CHS genome-wide association (GWA) study. Only individuals of Caucasian or European descent as ascertained by self-report of race/ethnicity were included in the present study, leaving 3291 subjects of European heritage who had GWA data. IGF-I and IGFBP-3 levels were obtained on a subset of CHS participants for a set of ancillary studies.

Framingham Heart Study (FHS):

FHS was first established in 1948 with the recruitment of a large cohort of adults from the town on Framingham, Massachusetts [5]. Since then, three generations of participants have been studied in this ongoing community-based cohort study of cardiovascular risk. The current study sample comprised the most contemporary generation of participants (Generation 3, recruited in 2002-2004). After excluding individuals with prevalent cardiovascular disease, renal impairment (defined as serum creatinine > 2mg/dl), diabetes, reduced renal function (estimated glomerular filtration rate < 50 ml/min/1.73m), missing IGF-I/IGFBP-3 measurements, or call rate less than 97%, a total of 3507 participants remained in the final sample. In FHS, fasting blood samples were collected according to strict protocol, centrifuged, and aliquotted immediately for storage at -70 C.

Framingham Heart Study (FHS) G2E7:

Participants were recruited from 2 generations in the Framingham community. Recruitment and characterization of the Framingham Study original cohort (generation 1) have been described in prior publications.11 A total of 5,209 participants were enrolled, and these participants were assessed in the Heart Study research clinic once every 2 years, where a detailed medical history was obtained and all traditional cardiovascular risk factors were measured. In 1971, offspring of persons in the original cohort and the spouses of these offspring were enrolled in the offspring cohort (generation 2) and assessed once every 4 years as described elsewhere.12 IGF-1 levels were measured in 789 participants from generation 1 (mean age 79 6 4 years, 64% women) of a total of 1,005 persons free of dementia who attended the 22nd examination cycle (1990–1994); the remaining persons, most assessed at home visits, did not have sufficient serum drawn to permit IGF-1 assay. IGF-1 levels were also measured in 2,793 participants from generation 2 (mean age 61 6 9 years, 55% women) of a total of 3,257 persons who attended examination 7 (1998–2001) and were free of dementia.

The Gothenburg Osteoporosis and Obesity Determinants (GOOD) study:

The GOOD study was initiated to determine both environmental and genetic factors involved in the regulation of bone and fat mass. Male study subjects were randomly identified in the greater Gothenburg area in Sweden using national population registers, contacted by telephone, and invited to participate. To be enrolled in the GOOD study, subjects had to be between 18 and 20 years of age. There were no other exclusion criteria, and 49% of the study candidates agreed to participate (n = 1,068). The study was approved by the ethics committee at the University of Gothenburg. Written and oral informed consent was obtained from all study participants .

Health2006 Study:

The Health2006 study is an ongoing cohort study of adults aged 18-69 years who live in the greater Copenhagen area. The aim of the study was to identify lifestyle related risk factors for chronic diseases such as diabetes, heart disease, asthma, musculoskeletal disorders, chronic lung disease and mental disorders. Baseline examinations were conducted between 2006 and 2008 and five-year follow-up examinations were finalized in 2011-2012. Data is collected through two questionnaires pertaining to lifestyle factors and mental health, and through medical exams assessing e.g. anthropometry, lung and cardiopulmonary function, and muscle strength. Blood samples were also collected from each participant for genetic and/or biomarker studies. The study was approved by the Ethical Committee of Copenhagen County and the Danish Data Protection Agency. A profile of the Health2006 cohort has been published elsewhere .

Health Aging and Body Composition (Health ABC) Study:

The Health Aging and Body Composition (Health ABC) Study is a NIA-sponsored ongoing cohort study aimed at assessing factors that contribute to incident disability and the decline in function of healthier older persons. In 1997/98, Health ABC enrolled well-functioning, community-dwelling black (n=1281) and white (n=1794) men and women aged 70-79 years. Participants were recruited from a random sample of Medicare eligible residents in the Pittsburgh, PA, and Memphis, TN, metropolitan areas. The key components of Health ABC include a baseline exam, annual follow-up clinical exams, and phone contacts every 6 months to identify major health events and document functional status between clinic visits.

GWAS data are available from 1663 white participants. Genomic DNA was extracted from buffy coat collected using PUREGENE® DNA Purification Kit during the baseline exam. Genotyping was performed by the Center for Inherited Disease Research (CIDR) using the Illumina Human1M-Duo BeadChip system. Genotyping was successful for 1,151,215 SNPs in 2,802 unrelated individuals (1663 Caucasians and 1139 African Americans).

The Invecchiare nel Chianti (InCHIANTI) study:

The Invecchiare nel Chianti (InCHIANTI) study is a population-based epidemiological study of adults aged 20 to 102 years and older (75% were aged 65 and over) that reside in Tscani, Italy. The InCHIANTI study was initiated in 1998 to evaluate factors associated with mobility disability in older adults who were randomly selected from the population registries of Greve in Chianti and Bagno a Ripoli using multi-level stratified sampling. Participants underwent a combination of assessments including blood tests, self-reported diagnoses and medication use as well as medical assessments by a trained geriatrician. InCHIANTI Study participants were evaluated for 3 subsequent follow-up visits every 3 years from baseline .

The Cooperative Research in the Region of Augsburg (KORA) study:

The Cooperative Research in the Region of Augsburg (KORA) study is a series of independent population-based epidemiological surveys and follow-up studies of participants living in the region of Augsburg, Southern Germany. All participants are residents of German nationality identified through the registration and informed consent has been given by all participants. The studies have been approved by the local ethics committee. The present study includes data of the follow-up study KORA F4 (2006-2008) of the KORA S4 survey (1999/2000). For genotyping, we included 1,814 randomly selected participants of KORA F4. Individuals with missing genotype or phenotype data were excluded, as were those having diabetes or reduced renal function (eGFR (Cockcroft Gault) <50 [ml/min/1,73m²]). Valid data were available in 1607 individuals.

Leiden Longevity Study (LLS):

The Leiden Longevity Study (LLS) is a family based study which consists of 1671 offspring of 421 nonagenarians sibling pairs of Dutch descent, and their 744 partners. 1910 individuals with available genotypic data and IGF1 and IGF1BP3 levels (mean age = 59.1 years) were included in the current analysis. The LLS participants were genotyped using Illumina Infinium HD Human660W-Quad BeadChips (Illumina, San Diego, CA, USA) and Illumina OmniExpress. Imputation was performed using IMPUTE with reference HapMap Phase I + II CEU release 21.

Microisolates in South Tyrol Study (MICROS):

The MICROS study is a population-based survey on adult volunteer participants who reside in three isolated villages in South Tyrol, Italy. These villages were selected because they had a small number of founders with old settlement, high rates of endogamy as well as slow/null population expansion. Extensive data was collected in 2002-03 regarding genealogy, and clinical measurements as well as collection of blood and urine samples and DNA isolation. An extensive standardized questionnaire was administered by interviewers to collect data on family history of disease and lifestyle exposures such as smoking and alcohol consumption. A serum sample was collected, prepared and stored at -80°C for subsequent analysis. The study was approved by the Ethics Committee of the Autonomous Province of Bolzano .

The Osteoporotic Fractures in Men (MrOS) Study Group:

The Osteoporotic Fractures in Men (MrOS) study is a multicenter, prospective study including older men in Sweden, Hong Kong, and the United States. The Gothenburg part (n = 1,010) of the Swedish MrOS cohort (n = 3,014) was included in the present study. The study subjects (men aged 69 to 80 years) were randomly identified using national population registers. A total of 45% of the subjects who were contacted participated in the study. To be eligible for the study, the subjects had to be able to walk without aids. There were no other exclusion criteria. The study was approved by the ethics committee at the University of Gothenburg. Informed consent was obtained from all study participants .

The Nurses’ Health Study (NHS):

The Nurses' Health Study is a prospective cohort study initiated in 1976 to study diseases in 121700 registered adult married female nurses who were 30 to 55 years old. Participants were enrolled through responding to a questionnaire inquiring about medical history, lifestyle practices, and demographic information. Follow-up questionnaires have been administered biennially since baseline to collect and update information regarding disease occurrence, medication, lifestyle, diet, and other risk factors of chronic diseases. In 1989-1990, a total of 32826 participants provided blood samples upon request. Whole blood samples were separated into plasma, buffy coat (white blood cells) and erythrocytes. All specimens are stored in the vapor phase of liquid nitrogen freezers. Among women who provided blood samples, several prospective nested case-control studies of major chronic diseases were conducted using the same study design. Briefly, among women who are free of the diseases of interest, we prospectively identify and confirm incident cases and randomly select 1-2 controls for each new case from those who remain to be free of the disease at the time of diagnosis of the new case. Data involved in this research were from three case-control studies of breast cancer, coronary heart disease, and type 2 diabetes

Prostate, Lung, Colorectal and Ovarian Cancer Screening Trial (PLCO):

The Prostate, Lung, Colorectal and Ovarian Cancer Screening Trial is a multicenter, two-armed, randomized trial in men and women aged 55-74 years. The goal of this study was to determine whether screening using chest X-ray, flexible sigmoidoscopy, prostate-specific antigen screening, digital rectal examination, cancer antigen 125 screening, and transvaginal ultrasound reduced mortality from lung, colorectal, prostate, and ovarian cancers. Other medical assessments included blood draw, pelvic exams and tissue biopsies. During follow-up, in the intervention arm, assessment using screening tools was repeated every 3 - 5 years while no assessments were completed for participants in the control arm. For all participants, cancer status (prostate, lung, colorectum, and ovary) as well as mortality data will be documented for a total of 13 years. The institutional review board at the National Cancer Institute approved this study .

The Rotterdam Study (RS):

The Rotterdam Study is an ongoing prospective cohort study of residents of the city of Rotterdam in the Netherlands who were 55 years and older. The study was started in 1990 to study age-related diseases such as cardiovascular, neurological, ophthalmological and endocrine diseases. Since its inception, additional participants have been added to the cohort to include those who moved into the study district, and those who lived in the district but had not previously participated. Participants were interviewed at home while assessments such as imaging, collection of bodily fluids and other clinical examinations were completed in a research facility. Follow-up examinations were repeated every 3–4 years to update health records with special attention to age-related diseases and repeat relevant clinical examinations. The institutional review boards of the Erasmus Medical Center and the Netherlands Ministry of Health, Welfare and Sports approved this study .

Study of Health in Pomerania (SHIP):

The Study of Health in Pomerania (SHIP) is a cohort study of community-dwelling adults aged 20 to 79 years old who reside in the north-east region of Germany. The cohort was recruited between 1997 and 2001 through a two-stage stratified cluster-sampling scheme. Extensive data was collected through oral health and medical examinations and self-administered questionnaires that assessed risk factors and health status. Bioelectric impedance, body plethysmography, cardiopulmonary exercise testing, sleep monitoring, and imaging such ultrasound and whole-body MRI were also conducted. Follow-up examinations have been conducted every 5-6 years since the baseline examination .

Study of Health in Pomerania (SHIP) TREND:

The Study of Health in Pomerania (SHIP) TREND is a second SHIP cohort that was recruited between 2008 and 2012 through stratified random sampling of adults age 20-79 years old. The primary goals of this additional cohort were to study the prevalence of subclinical diseases identified using non-invasive methods and to analyze determinants of subclinical and clinical diseases. Similar to SHIP, SHIP-TREND collected data through extensive questionnaires and medical examinations .

SORBS:

All subjects are part of a sample from an extensively phenotyped population from Eastern Germany, the Sorbs. At present, about 1000 Sorbian individuals are enrolled in the study. Extensive phenotyping included standardised questionnaires for past medical history and family history, collection of anthropometric data and a 75g-glucose-tolerance-test. Genotyping was performed using 500K Affymetrix GeneChip (Affymetrix, Inc) and Affymetrix Genome-Wide Human SNP Array 6.0. The study was approved by the ethics committee of the University of Leipzig and all subjects gave written informed consent before taking part in the study.

**Funding acknowledgment**

BLSA: Funded by the intramural Research Program of the NIH, National Institute on Aging.

CHS: This CHS research was supported by NHLBI contracts HHSN268201200036C, HHSN268200800007C, N01HC55222, N01HC85079, N01HC85080, N01HC85081, N01HC85082, N01HC85083, N01HC85086; and NHLBI grants U01HL080295, R01HL087652, R01HL105756, R01HL103612, and R01HL120393 with additional contribution from the National Institute of Neurological Disorders and Stroke (NINDS). Additional support was provided by AG023629 and R01AG027058 and 1R01AG031890 from the National Institute on Aging (NIA). A full list of principal CHS investigators and institutions can be found at [CHS-NHLBI.org](https://owa.yu.edu/owa/redir.aspx?C=MGgP2Ss5jEey2JbJGvrge7ZWyDV_M9IIuibiph1vbLk9eNO0tzF_08b34c7nGWLKdOOIvBHPf4c.&URL=http%3A%2F%2Fchs-nhlbi.org%2F). The provision of genotyping data was supported in part by the National Center for Advancing Translational Sciences, CTSI grant UL1TR000124, and the National Institute of Diabetes and Digestive and Kidney Disease Diabetes Research Center (DRC) grant DK063491 to the Southern California Diabetes Endocrinology Research Center. The content is solely the responsibility of the authors and does not necessarily represent the official views of the National Institutes of Health.

FHS: This research was conducted using data and resources from the Framingham

Heart Study of the National Heart Lung and Blood Institute of the National

Institutes of Health and Boston University School of Medicine based on

analyses by Framingham Heart Study investigators participating in the SNP

Health Association Resource (SHARe) project. This work was supported by the

National Heart, Lung and Blood Institute's Framingham Heart Study (Contract

No. N01-HC-25195) and its contract with Affymetrix, Inc for genotyping

services (Contract No.N02-HL-6-4278). A portion of this research utilized

the Linux Cluster for Genetic Analysis (LinGA-II) funded by the Robert

Dawson Evans Endowment of the Department of Medicine at Boston University

School of Medicine and Boston Medical Center. For the FHS.G2E7, funding was provided by NIA R01 AG031287

GOOD: Financial support was received from the Swedish Research Council, the Swedish Foundation for Strategic Research, the ALF/LUA research grant in Gothenburg, the Lundberg Foundation, the Torsten and Ragnar Söderberg’s Foundation, the Novo Nordisk Foundation, and the European Commission grant HEALTH-F2-2008-201865-GEFOS.

Health2006: The Health2006 study was financially supported by grants from the Velux Foundation; the Danish Medical Research Council, Danish Agency for Science, Technology and Innovation; the Aase and Ejner Danielsens Foundation; ALK-Abello´ A/S (Hørsholm, Denmark), Timber Merchant Vilhelm Bangs Foundation, MEKOS Laboratories (Denmark) and Research Centre for Prevention and Health, the Capital Region of Denmark. The Novo Nordisk Foundation Center for Basic Metabolic Research is an independent Research Center at the University of Copenhagen partially funded by an unrestricted donation from the Novo Nordisk Foundation (www.metabol.ku.dk).

HABC: NIA contracts N01AG62101, N01AG62103, and N01AG62106. The genome-wide association study was funded by NIA grant 1R01AG032098-01A1 to Wake Forest University Health Sciences and genotyping services were provided by the Center for Inherited Disease Research (CIDR). CIDR is fully funded through a federal contract from the National Institutes of Health to The Johns Hopkins University, contract number HHSN268200782096C. This research was supported in part by the Intramural Research Program of the NIH, National Institute on Aging. The Health, Aging, and Body Composition (HABC) Study is supported by NIA contracts N01AG62101, N01AG62103, and N01AG62106. The genome-wide association study was funded by NIA grant 1R01AG032098-01A1 to Wake Forest University Health Sciences.

InCHIANTI: The InCHIANTI study baseline (1998-2000) was supported as a "targeted project" (ICS110.1/RF97.71) by the Italian Ministry of Health and in part by the U.S. National Institute on Aging (Contracts: 263 MD 9164 and 263 MD 821336).

KORA: Helmholtz Center Munich, German Research Center for Environmental Health, which is funded by the German Federal Ministry of Education and Research (BMBF) and by the State of Bavaria. Part of this work was financed by the German National Genome Research Network (NGFN-2 and NGFNPlus: 01GS0823). Our research was supported within the Munich Center of Health Sciences (MC Health) as part of LMUinnovativ. This study was in part supported by a grant from the German Federal Ministry of Education and Research (BMBF) to the German Center for Diabetes Research (DZD e.V.)

LLS: Funding received from the European Union’s Seventh Framework Programme (FP7/2007-2011) under grant agreement number 259679. This study was financially supported by the Innovation-Oriented Research Program on Genomics (SenterNovem IGE05007), the Centre for Medical Systems Biology and the Netherlands Consortium for Healthy Ageing (grant 050-060-810), all in the framework of the Netherlands Genomics Initiative, Netherlands Organization for Scientific Research (NWO), by Unilever Colworth and by BBMRI-NL, a Research Infrastructure financed by the Dutch government (NWO 184.021.007).

MICROS: We thank the primary care practitioners Raffaela Stocker, Stefan Waldner, Toni Pizzecco, Josef Plangger, Ugo Marcadent, and the personnel of the Hospital of Silandro (Department of Laboratory Medicine) for their participation and collaboration in the research project. The MICROS study was supported by the Ministry of Health and Department of Educational Assistance, University and Research of the Autonomous Province of Bolzano, the South Tyrolean Sparkasse Foundation, and the European Union framework program 6 EUROSPAN project (contract no. LSHG-CT-2006-018947)

MrOS Sweden: Financial support was received from the Swedish Research Council, the Swedish Foundation for Strategic Research, the ALF/LUA research grant in Gothenburg, the Lundberg Foundation, the Torsten and Ragnar Söderberg’s Foundation, the Novo Nordisk Foundation, and the European Commission grant HEALTH-F2-2008-201865-GEFOS.

Nurses' Health Study (NHS): NIH grants CA186107 and CA49449; NHS.CGEM: NIH grants CA87969, CA40356, and U01CA98233; NHS.CHD: Supported by NIH grants HL35464 and HL034594; the genotyping of the NHS.CHD was supported by an unrestricted grant from Merck Research Laboratories, North Wales, PA; NHS.T2D: Supported by NIH grants DK080792, DK58845, DK58785, U01HG004446, and U01HG004399. Qi Sun was supported by a career development grant R00HL098459 from the NHLBI. We would like to thank the participants and staff of the NHS and HPFS for their valuable contributions as well as the following state cancer registries for their help: AL, AZ, AR, CA, CO, CT, DE, FL, GA, ID, IL, IN, IA, KY, LA, ME, MD, MA, MI, NE, NH, NJ, NY, NC, ND, OH, OK, OR, PA, RI, SC, TN, TX, VA, WA, WY. The authors assume full responsibility for analyses and interpretation of these data.

PLCO: Supported by contracts from the National Cancer Institute to 10 PLCO screening centers, a coordinating center and a central analytic laboratory; grants from the National Cancer Institute (P50 CA083636 to NU, P50 CA083639 to R.C. Bast, 5U01 CA86381 to DWC, R01 CA127913 and U01 CA084986 to G. Mor) <<RK checking with Sonja)

Rotterdam Study: We thank Pascal Arp, Mila Jhamai, Marijn Verkerk, Lizbeth Herrera and Marjolein Peters for their help in creating the GWAS database, and Karol Estrada and Maksim V. Struchalin for their support in creation and analysis of imputed data. The authors are grateful to the study participants, the staff from the Rotterdam Study and the participating general practitioners and pharmacists.The generation and management of GWAS genotype data for the Rotterdam Study is supported by the Netherlands Organization for Scientific Research NWO Investments (nr. 175.010.2005.011, 911-03-012). This study is funded by the Research Institute for Diseases in the Elderly (014-93-015; RIDE2), the Netherlands Genomics Initiative (NGI)/Netherlands Organization for Scientific Research (NWO) project nr. 050-060-810, CHANCES (nr 242244). The Rotterdam Study is funded by Erasmus Medical Center and Erasmus University, Rotterdam, Netherlands Organization for the Health Research and Development (ZonMw), the Research Institute for Diseases in the Elderly (RIDE), the Ministry of Education, Culture and Science, the Ministry for Health, Welfare and Sports, the European Commission (DG XII), and the Municipality of Rotterdam.

SHIP: SHIP is part of the Community Medicine Research net of the University of Greifswald, Germany, which is funded by the Federal Ministry of Education and Research (grants no. 01ZZ9603, 01ZZ0103, and 01ZZ0403), the Ministry of Cultural Affairs as well as the Social Ministry of the Federal State of Mecklenburg-West Pomerania, and the network ‘Greifswald Approach to Individualized Medicine (GANI_MED)’ funded by the Federal Ministry of Education and Research (grant 03IS2061A). Genome-wide data have been supported by the Federal Ministry of Education and Research (grant no. 03ZIK012) and a joint grant from Siemens Healthcare, Erlangen, Germany and the Federal State of Mecklenburg- West Pomerania. The University of Greifswald is a member of the ‘Center of Knowledge Interchange’ program of the Siemens AG and the Caché Campus program of the InterSystems GmbH. The SHIP authors are grateful to Holger Prokisch and Thomas Meitinger (Helmholtz Zentrum München) for the genotyping of the SHIP-TREND cohort.

SORBS: We thank Ayman Arafat for the measurement of IGF-I. This work was supported by grants from the German Research Council (SFB- 1052 “Obesity mechanisms”, SPP 1629 TO 718/2-1), from the German Diabetes Association and from the DHFD (Diabetes Hilfs- und Forschungsfonds Deutschland). Peter Kovacs is funded by the Boehringer Ingelheim Foundation. IFB AdiposityDiseases is supported by the Federal Ministry of Education and Research (BMBF), Germany, FKZ: 01EO1001. Inga Prokopenko was funded in part through the European Community's Seventh Framework Programme (FP7/2007-2013), ENGAGE project, grant agreement HEALTH-F4-2007-201413.

**References**

1 Haydar ZR, Blackman MR, Tobin JD, Wright JG and Fleg JL. (2000) The relationship between aerobic exercise capacity and circulating IGF-1 levels in healthy men and women. *Journal of the American Geriatrics Society*, **48**, 139-145.

2 Lorentzon M, Swanson C, Andersson N, Mellstrom D and Ohlsson C. (2005) Free testosterone is a positive, whereas free estradiol is a negative, predictor of cortical bone size in young Swedish men: the GOOD study. *Journal of bone and mineral research : the official journal of the American Society for Bone and Mineral Research*, **20**, 1334-1341.

3 Thuesen BH, Cerqueira C, Aadahl M, Ebstrup JF, Toft U *et al.* (2014) Cohort Profile: the Health2006 cohort, research centre for prevention and health. *International journal of epidemiology*, **43**, 568-575.

4 Ferrucci L, Bandinelli S, Benvenuti E, Di Iorio A, Macchi C *et al.* (2000) Subsystems contributing to the decline in ability to walk: bridging the gap between epidemiology and geriatric practice in the InCHIANTI study. *J Am Geriatr Soc*, **48**, 1618-1625.

5 Pattaro C, Marroni F, Riegler A, Mascalzoni D, Pichler I *et al.* (2007) The genetic study of three population microisolates in South Tyrol (MICROS): study design and epidemiological perspectives. *BMC medical genetics*, **8**, 29.

6 Mellstrom D, Johnell O, Ljunggren O, Eriksson AL, Lorentzon M *et al.* (2006) Free testosterone is an independent predictor of BMD and prevalent fractures in elderly men: MrOS Sweden. *Journal of bone and mineral research : the official journal of the American Society for Bone and Mineral Research*, **21**, 529-535.

7 Sun Q, Jimenez MC, Townsend MK, Rimm EB, Manson JE *et al.* (2014) Plasma levels of fetuin-A and risk of coronary heart disease in US women: the Nurses' Health Study. *Journal of the American Heart Association*, **3**, e000939.

8 Rajpathak SN, He M, Sun Q, Kaplan RC, Muzumdar R *et al.* (2012) Insulin-like growth factor axis and risk of type 2 diabetes in women. *Diabetes*, **61**, 2248-2254.

9 Hankinson SE, Willett WC, Colditz GA, Hunter DJ, Michaud DS *et al.* (1998) Circulating concentrations of insulin-like growth factor-I and risk of breast cancer. *Lancet*, **351**, 1393-1396.

10 Prorok PC, Andriole GL, Bresalier RS, Buys SS, Chia D *et al.* (2000) Design of the Prostate, Lung, Colorectal and Ovarian (PLCO) Cancer Screening Trial. *Controlled clinical trials*, **21**, 273S-309S.

11 Hofman A, Breteler MM, van Duijn CM, Janssen HL, Krestin GP *et al.* (2009) The Rotterdam Study: 2010 objectives and design update. *European journal of epidemiology*, **24**, 553-572.

12 John U, Greiner B, Hensel E, Ludemann J, Piek M *et al.* (2001) Study of Health In Pomerania (SHIP): a health examination survey in an east German region: objectives and design. *Sozial- und Praventivmedizin*, **46**, 186-194.

13 Volzke H, Alte D, Schmidt CO, Radke D, Lorbeer R *et al.* (2011) Cohort profile: the study of health in Pomerania. *International journal of epidemiology*, **40**, 294-307.
